# Profiling the neurovascular unit unveils detrimental effects of osteopontin on the blood–brain barrier in acute ischemic stroke

**DOI:** 10.1007/s00401-022-02452-1

**Published:** 2022-06-25

**Authors:** Daniel Spitzer, Sylvaine Guérit, Tim Puetz, Maryam I. Khel, Moritz Armbrust, Maika Dunst, Jadranka Macas, Jenny Zinke, Gayatri Devraj, Xiaoxiong Jia, Florian Croll, Kathleen Sommer, Katharina Filipski, Thomas M. Freiman, Mario Looso, Stefan Günther, Mariangela Di Tacchio, Karl-Heinz Plate, Yvonne Reiss, Stefan Liebner, Patrick N. Harter, Kavi Devraj

**Affiliations:** 1 Edinger Institute (Institute of Neurology), University Hospital, Goethe University, 60528 Frankfurt, Germany; 2 Department of Neurology, University Hospital, Goethe University, 60528 Frankfurt, Germany; 3 Institute for Medical Microbiology and Infection Control, University Hospital, Goethe University, 60528 Frankfurt, Germany; 4 Department of Neurosurgery, University Medical Center Rostock, 18057 Rostock, Germany; 5 Max Planck Institute for Heart and Lung Research, 61231 Bad Nauheim, Germany; 6 German Cancer Consortium (DKTK) Partner site Frankfurt/Mainz, 60528 Frankfurt, Germany; 7 German Cancer Research Center (DKFZ), 69120 Heidelberg, Germany; 8 Frankfurt Cancer Institute (FCI), 60528 Frankfurt, Germany; 9 German Center for Cardiovascular Research (DZHK), Partner Site Frankfurt/Mainz, 60528 Frankfurt, Germany; 10 Excellence Cluster Cardio Pulmonary System (CPI), Partner Site Frankfurt, 60528 Frankfurt, Germany; 11 LOEWE - Center for Personalized Translational Epilepsy Research (CePTER), Goethe University, 60528 Frankfurt, Germany

**Keywords:** Neurovascular unit (NVU), Blood–brain barrier (BBB), Stroke, RNA-sequencing, Osteopontin, EPAM-ia

## Abstract

Blood–brain barrier (BBB) dysfunction, characterized by degradation of BBB junctional proteins and increased permeability, is a crucial pathophysiological feature of acute ischemic stroke. Dysregulation of multiple neurovascular unit (NVU) cell types is involved in BBB breakdown in ischemic stroke that may be further aggravated by reperfusion therapy. Therefore, therapeutic co-targeting of dysregulated NVU cell types in acute ischemic stroke constitutes a promising strategy to preserve BBB function and improve clinical outcome. However, methods for simultaneous isolation of multiple NVU cell types from the same diseased central nervous system (CNS) tissue, crucial for the identification of therapeutic targets in dysregulated NVU cells, are lacking. Here, we present the EPAM-ia method, that facilitates simultaneous isolation and analysis of the major NVU cell types (endothelial cells, pericytes, astrocytes and microglia) for the identification of therapeutic targets in dysregulated NVU cells to improve the BBB function. Applying this method, we obtained a high yield of pure NVU cells from murine ischemic brain tissue, and generated a valuable NVU transcriptome database (https://bioinformatics.mpi-bn.mpg.de/SGD_Stroke). Dissection of the NVU transcriptome revealed *Spp1*, encoding for osteopontin, to be highly upregulated in all NVU cells 24 h after ischemic stroke. Upregulation of osteopontin was confirmed in stroke patients by immunostaining, which was comparable with that in mice. Therapeutic targeting by subcutaneous injection of an anti-osteopontin antibody post-ischemic stroke in mice resulted in neutralization of osteopontin expression in the NVU cell types investigated. Apart from attenuated glial activation, osteopontin neutralization was associated with BBB preservation along with decreased brain edema and reduced risk for hemorrhagic transformation, resulting in improved neurological outcome and survival. This was supported by BBB-impairing effects of osteopontin in vitro. The clinical significance of these findings is that anti-osteopontin antibody therapy might augment current approved reperfusion therapies in acute ischemic stroke by minimizing deleterious effects of ischemia-induced BBB disruption.

**Supplementary Information:**

The online version contains supplementary material available at 10.1007/s00401-022-02452-1.

## Introduction

Stroke is the second leading cause of death and ranks among the main causes of disability and invalidity, imposing great health and economic burden on society [39, 134]. This applies in particular for ischemic stroke that accounts for 87% of all strokes [88]. One of the major pathophysiological features is dysfunction of the blood–brain barrier (BBB) contributing to major clinical complications. Therefore, the search for new effective therapies to reduce the burden of stroke has led to a paradigm shift in stroke research and treatment from a neuroprotective approach to improvement of neurovascular function [76, 82]. The neurovascular unit (NVU) comprises endothelial cells [51, 75, 132], pericytes [4, 23, 46, 106, 111], astrocytes [1, 55, 129, 133], and microglia [35, 44, 95, 108], which, together with neurons [84], critically regulate and maintain BBB integrity through a continuous and dynamic neurovascular interaction. The NVU crosstalk maintains BBB integrity by preserving endothelial cell–cell junctions and regulating transendothelial transport, making the BBB endothelium a highly selective barrier [51, 132], thus maintaining homeostasis within the central nervous system (CNS) and brain health [97]. Dysregulation of NVU cells plays a key role in pathophysiological processes of ischemic stroke that is characterized by BBB breakdown [112]. A crucial feature underlying the latter is the degradation of BBB junctional proteins that occurs early after ischemic stroke [2, 56, 67, 82]. This substantially contributes to the development of acute severe complications including vasogenic brain edema [118, 122] and hemorrhagic transformation [64, 122], further exacerbating cerebral injury [96] and increasing the risk of poor clinical outcome in patients [67, 115]. Moreover, ultrastructural alterations of BBB components and its associated complications may be aggravated by reperfusion therapy [56, 63, 67, 113]. Therefore, it might be a promising accessory strategy to co-target multiple dysregulated NVU cell types to preserve BBB integrity and further prevent acute complications in stroke patients.

Restoration of a functional NVU is the prerequisite for BBB recovery in ischemic stroke [112, 127]. However, this can be challenging as it requires understanding of the specific contributions of dysregulated NVU cell interactions to barrier dysfunction [127]. At present, there are no therapeutic approaches co-targeting multiple NVU cell types to minimize BBB perturbations in ischemic stroke [99]. Moreover, there are limited methods to identify and validate specific mechanisms of BBB damage caused by NVU dysregulation, thus leaving important questions on this subject unanswered [93, 127].

Current approaches investigating NVU dysfunction and BBB impairment utilize isolation of microvessels, cultured endothelial cells and more recently FACS sorted cells. These strategies have major limitations, as isolated microvessels contain several NVU cells, leading to difficulties in assessing the cellular source of BBB dysfunction. Cultured cells, in particular endothelial cells, tend to dedifferentiate in vitro, making their use limited to permeability studies [81]. More recently, FACS-based procedures have been applied for isolating different cell types of the NVU, such as endothelial cells, pericytes, astrocytes and microglia [3, 19, 22, 46, 111, 128]. However, at best two cell types of the NVU have been isolated simultaneously from the same sample [19]. A major drawback of these studies is that they employ transgenic mice for each cell type with genetically labeled cells for isolation and sorting. Moreover, some of the above studies are based on single-cell sorting, which is tedious, expensive and not easily applicable to disease states.

Despite increasing evidence that NVU cells interact with each other in regulating BBB permeability [112, 127, 132], a highly translational method for the simultaneous isolation of multiple NVU cell types and analysis of their interactions in CNS diseases associated with BBB impairment is lacking. A key challenge in stroke research and treatment is to identify crucial mechanisms leading to BBB breakdown, define new therapeutic targets involved in NVU dysfunction, and develop robust therapies preserving BBB integrity by restoring normal NVU function. Here we present a method that we call for simplicity EPAM-ia method that allows simultaneous isolation and analysis of four major NVU cell types—endothelial cells, pericytes, astrocytes and microglia—from the same specimen. This method incorporates a combination of mechanical homogenization, filtration and enzymatic digestion adjusted to each cell type. The resulting single cell suspension of brain tissue is sorted by flow cytometry utilizing established markers for each NVU cell type while incorporating exclusion markers for contaminating cells.

Application of the EPAM-ia method to healthy and ischemic brain tissue 24 h post-insult of wild type mice revealed several genes differentially expressed and/or regulated in multiple NVU cell types, resulting in a new transcriptome database applicable for the investigation of NVU dysfunction in acute ischemic stroke. Bioinformatic dissection of this NVU transcriptome database revealed osteopontin (OPN), encoded by the *Spp1* gene, to be dramatically upregulated in all the NVU cell types during the acute phase of ischemic stroke that was further confirmed in human brain specimens obtained from subjects with cerebral infarction. Assessing the early effects of OPN, we confirm that this protein impairs BBB function in vitro and demonstrate in an in vivo model of ischemic stroke that therapeutic neutralization using an anti-OPN antibody preserves the integrity of the BBB. Furthermore, the observed protection of the BBB results in decreased brain edema and a reduced risk for hemorrhagic transformation, as well as improved neurological outcome and survival, demonstrating that the anti-OPN antibody therapy might be a new potential therapeutic approach in acute ischemic stroke treatment.

Our strategy thus involves identification of a specific target dysregulated in multiple NVU cell types based on the EPAM-ia method, followed by its therapeutic co-targeting at the NVU to minimize BBB dysfunction and the neurological sequalae in CNS diseases associated with BBB impairment.

## Materials and methods

### Animal care and handling

Adult (12–15 weeks) wild-type (WT) C57BL/6 mice of both sexes were used in the study unless otherwise specified. Animals were housed in groups of three to five per cage under standard specific-pathogen-free (SPF) conditions in a temperature-, humidity- and light cycle-controlled facility (20 ± 2 °C; 50 ± 10%; 12 h light/dark cycle) with free access to food and water. All animals were sacrificed by cervical dislocation under deep isoflurane anesthesia and their number was kept to a minimum based on extracted tissue/cell amount and statistically appropriate sample size. All the in vivo experiments were in compliance with the ARRIVE guidelines. All experiments using animals were strictly conducted in accordance to the German Protection of Animals Act and in compliance with the recommendations in the Guide for Care and Use of Laboratory Animals of the National Institutes of Health, and were approved by the local governmental authorities (Regierungspraesidium Darmstadt, Germany; approval number FK/1052).

### Isolation of multiple NVU cell types by the EPAM-ia method

#### Mouse brain tissue collection and preparation of single cell suspension

After animals were sacrificed, brains were extracted from the skull under the semi-sterile hood and stored in ice cold DPBS. Cerebrum was separated from olfactory lobes, cerebellum and hindbrain, and the leptomeninges peeled off by rolling on sterile Whatman paper. Cerebrum was then transferred to a petri dish and minced finely followed by incubation for 45 min at 37 °C with mild shaking in Digestion mix 1 (0.025% papain, Worthington #LS003126, and 0.001% DNase1, Worthington #LS006333, in DPBS). After centrifugation (400*g*, 5 min, 4 °C, unless otherwise indicated) and a DPBS wash to remove the enzymes, the remaining pellet was resuspended in DPBS and filtered through a 10 µm mesh (Pluriselect, #43-50010-50). The flow through was stored on ice until the myelin removal step. The mesh was then flipped, placed on a new falcon tube and vessel fragments were collected by flushing with Buffer A (153 mM NaCl, 5.6 mM KCl, 1.7 mM CaCl_2_, 1.2 mM MgCl_2_, 15 mM HEPES 15 mM, 1% BSA; pH 7.4). The vessel fragments suspension was then homogenized by 15–20 strokes (1 stroke = 1 up + 1 down) using a tight homogenizer (clearance 0.025 mm, Wheaton, #358013) connected to an overhead electric stirrer (2000 rpm, 45 W, VWR, #VOS14). Post-centrifugation, the vessel fragments were incubated for 45 min at 37 °C, 600 rpm (Eppendorf, #compact 5436) in the Digestion Mix 2 (0.25% collagenase II, Biochrom #C2-28, in Buffer A). Both on-mesh vessel fragments and flow through samples were then centrifuged and the cell pellets resuspended thoroughly in 25% BSA in DBPS and centrifuged for 20 min at 2000*g*, 4 °C. The two upper layers containing the myelin debris and the BSA were discarded. The cell pellet derived from the flow through was resuspended in DPBS and stored on ice, whereas the vessel fragments were incubated for 15 min at 37 °C, 600 rpm in the Digestion Mix 3 (0.1% collagenase/dispase, Roche #10269638001, and 0.0001% DNase1 in Buffer A). After centrifugation and removal of the supernatant containing the enzymes, the cell pellets of the flow through and the corresponding digested vessel fragments were combined, washed once and resuspended in FACS buffer (5% serum in PBS, 0.5 mL/brain) to obtain the final single cell suspension for immunolabeling.

#### Immunolabeling of cell suspension

Cells were counted using a hemocytometer (Neubauer's counting chamber, Carl Roth #T729.1) and resuspended in FACS buffer to obtain 5 × 10^6^ cells/mL. From each sample, 5–10% of the cell suspension was collected to generate the unstained and the single stained controls for compensation. Samples were protected from light and incubated in the fridge for 30 min with the following antibodies: APC anti-ACSA2 (clone IH3-18A3, 1/50. Miltenyi Biotech, #130-102-315); BV510 anti-CD11b (clone M1/70, 1/100. BD Bioscience, #562950); PerCp-Cy5.5 anti-CD45 (clone 30-F11, 1/100. eBioscience, #45-0451-82); PE anti-NG2/AN2 (clone 1E6.4, 1/20. Miltenyi Biotech, #130-097-458); APC-Vio-770 anti-PDGFRβ/CD140b (clone REA634, 1/25. Miltenyi Biotech, #130-109-870); and PE-Cy7 anti-VECAD/CD144 (clone BV13, 1/50. Bio Legend, #138016). Post-incubation, samples were washed twice with FACS buffer and finally resuspended in 300 µL of FACS buffer per brain and stored on ice until sorting.

#### Sorting of NVU cells by flow cytometry

Prior to cell sorting, samples were gently resuspended and filtered through a 50 µm cell strainer (Bio-Rad, #12012582) to remove cell clumps. DAPI (1/100 of 10 µM stock solution) was added to the cell suspension and incubated for 3–5 min followed by flow cytometry and sorting. After exclusion of debris (FSC/SSC gating) and dead cells (DAPI^pos^), live cells (DAPI^neg^) were plotted for CD45 and microglia population was collected as CD45^low^/CD11b^pos^ cells. CD45^neg^ cells were selected for the isolation of astrocytes, endothelial cells and pericytes to remove non-specifically stained immune cells. After plotting for ACSA2, CD45^neg^/ACSA2^high^ cells were selected, and astrocytes were then collected as CD45^neg^/ACSA2^high^/NG2^neg^/PDGFRβ^neg^/VECAD^neg^ population. For the isolation of endothelial cells and pericytes, CD45^neg^/ACSA2^neg^ cells were first selected and plotted for VECAD. ACSA2^neg^/VECAD^pos^ cells were further selected, and endothelial cells were collected as NG2^neg^/PDGFRβ^neg^ population. Finally, CD45^neg^/ACSA2^neg^/VECAD^neg^ cells were selected and pericytes were collected as NG2/PDGFRβ double positive cells. All the cells were collected directly in RLT + lysis buffer (RNeasyPlus microkit, Qiagen, #74034) using a FACS Aria II flow cytometer (BD Bioscience) and stored at − 80 °C until RNA isolation.

#### NVU cells yield analysis

To obtain the yield of sorted cells, FACS data was analyzed using FlowJo software (v10.0.8, FlowJo, LLC). Dead cells were expressed as a percentage of the all-cell population (FSC/SSC gate). Endothelial cells, pericytes, astrocytes and microglia numbers were normalized to 1 × 10^6^ live cells (i.e., DAPI^neg^).

### RNA preparation and purity analysis by qRT–PCR

NVU cells in RLT + buffer were thawed on ice and vortexed for 1 min to ensure efficient lysis. RNA was isolated according to manufacturer’s recommendations (RNeasyPlus microkit, Qiagen, #74034) and eluted with 20µL of RNAse free water. A 2µL aliquot was used for quantity and quality analysis with the Experion RNA HighSens analysis kit (Bio-Rad, # 7007105). For purity analysis of the sorted populations, 11 µL of RNA solution were used to generate cDNA following the recommendation of the RevertAidTM H minus First strand cDNA Synthesis kit (Thermo Fisher, #K1632) and residual RNA digested using RNase H (NewEngland BioLabs, #M0297S). Quantitative RT–PCR (qRT–PCR) was performed using the Absolute qPCR SYBR Green Fluorescein Mix (Thermo Scientific, #AB-1219) according to the manufacturer's protocol with an annealing temperature of 61 °C (Biorad, CFX96). Detailed information on primer pairs used for the amplification of cell-specific genes is shown in the supplementary material (Supplementary Table 1, online resource). Quantitative RT–PCR data was analyzed (CFX 3.1 software, Biorad) and threshold cycles (Ct) were exported to a spreadsheet and expression obtained by Delta (∆∆Ct) method relative to the housekeeping gene.

### Mouse model of transient middle cerebral artery occlusion (tMCAO) and downstream processing

MCAO surgeries were performed as described [41]. Briefly, male mice were anesthetized with 1.5% isofluorane followed by right MCA occlusion using standardized monofilament (Doccol Corporation, #602256PK10, #602056PK10). The filament was withdrawn after 60 min to allow reperfusion of the ischemic hemisphere. Animals were sacrificed 24 h post-occlusion right after assessment of their global neurological functions with a 14-points modified Neurological Severity Score [16] (mNSS; Supplementary Table 2, online resource). As previously performed analysis indicated no major changes between tMCAO-operated contralateral and sham-operated ipsilateral brain microvessels [65], we, therefore, utilized contralateral hemispheres from ischemic animals as control instead of sham-operated animals. Pooled cerebra (*n* = 3–4 per experiment) from ipsilateral (ischemic) and contralateral (control) hemispheres were finally subjected to isolation of NVU cells by flow cytometry according to the procedures mentioned above. Animals subjected to tMCAO for the purpose of NVU cell isolation were excluded if mNSS was less than 8 (11 mice) to include animals with moderate to severe ischemia [7]. The mNSS was averaged to represent each biological replicate, which is a pooled sample. Animals that died before the end of the 24 h observation period (mortality ratio: 32%) were also excluded from the transcriptomic study.

### Bulk RNA-Sequencing and bioinformatic analysis of NVU cells isolated from ischemic brain tissue

For bulk RNA-Sequencing, RNA was isolated from the NVU cells sorted using the EPAM-ia method from both ipsilateral and contralateral murine stroke samples (males only) using the RNeasy Plus micro kit (Qiagen, #74034) combined with on-column DNase digestion (DNase-Free DNase Set, Qiagen, #79254) to avoid contamination by genomic DNA. RNA and library preparation integrity were verified with LabChip Gx Touch 24 (Perkin Elmer). Approx. 250 pg of total RNA was used as input for SMART^®^-Seq HT kit (Takara Clontech). Sequencing was performed on the NextSeq500 instrument (Illumina) using v2 chemistry, resulting in average of 20 M reads per library with 1 × 75 bp single end setup. The raw reads were assessed for quality, adapter content and duplication rates with FastQC (Andrews S. 2010, FastQC: a quality control tool for high throughput sequence data: www.bioinformatics.babraham.ac.uk/projects/fastqc). Trimmomatic version 0.38 was employed to trim reads after a quality drop below a mean of Q20 in a window of 10 nucleotides [9]. Only reads between 30 and 150 nucleotides were cleared for further analyses. Trimmed and filtered reads were aligned versus the Ensembl mouse genome version mm10 (GRCm38) using STAR 2.6.1d with the parameter “-outFilterMismatchNoverLmax 0.1” to increase the maximum ratio of mismatches to mapped length to 10% [29]. The number of reads aligning to genes was counted with featureCounts 1.6.3 tool from the Subread package [73]. Only reads mapping at least partially inside exons were admitted and aggregated per gene. Reads overlapping multiple genes or aligning to multiple regions were excluded. Differentially expressed genes were identified using DESeq2 version 1.18.1 [78]. Only genes with minimum reads > 10 in all biological replicates of a particular sample were considered as expressed in that sample. Furthermore, only genes with a minimum fold change of ± 1.5 (log2FC ± 0.585) and a maximum Benjamini–Hochberg corrected *P* value of 0.05 were deemed to be differentially regulated. The Ensemble annotation was enriched with UniProt data (release 06.06.2014) based on Ensembl gene identifiers [Activities at the Universal Protein Resource (UniProt)]. For bioinformatic pathway analysis, differentially expressed genes were submitted to gene set enrichment analyses with KOBAS [121]. The resulting plots showing pathways with *P* value < 0.05 (represented by dashed line) were obtained for each cell type.

### Upset analysis for commonly regulated genes in NVU cells

Our aim was to analyze the commonly regulated genes from all four NVU cell types in the ischemic stroke hemisphere compared to contralateral hemisphere. To analyze intersections of these multiple sets, we utilized the recently developed UpSet analysis [72], as visualization of intersections of multiple sets is not possible by classic Euler or Venn diagrams. To this end, expression and regulation data for each cell type were transformed to binary data as described below. For expression, we assigned 1 for a gene in a particular sample if its reads were > 10 in all of the biological replicates of that particular sample otherwise it was assigned 0. We assigned 1 for regulation if significant from the initial bulk RNA-Sequencing data AND if the expression values for the gene in all biological replicates of the contralateral and/or the ischemic hemisphere were > 10 reads, otherwise it was assigned 0. The above logical functions were performed using a spreadsheet software (MS Office). This binary transformation resulted in a total of 12 sets, i.e., for the four cell types with three sets for each cell type for a particular gene—expression in contralateral, expression in ischemic stroke and differential regulation between the contralateral and ischemic stroke hemispheres for that gene in that cell type. With UpSet, we could analyze the 2^12^, i.e., 4096 intersections. However, empty intersections were ignored. The.CSV text file with the above binary data including mean expression reads and Log2FC for all the cell types for both contralateral and ischemic hemispheres were uploaded to the GitHub repository (https://github.com/SGD2020/mcao/blob/master/12csvnew.csv, can be downloaded by a right-click) and a JSON file was created within the GitHub site (included at the url: https://raw.githubusercontent.com/SGD2020/mcao/master/mcao.json). The UpSet analysis can be visualized by going to the upset site: http://vcg.github.io/upset/ followed by submitting the above JSON file link in the load data address bar.

### Human stroke specimen

All studies on human stroke subjects (Supplementary Table 3, online resource) were approved by an ethics statement (ethics approval number for autopsy material GS-249/11 and for resection material GS-04/09, Edinger Institute). Stage I tissues were obtained 24–48 h post-vessel occlusion and present acute necrosis. Stage II is defined by macrophage resorption and stage III by the observation of pseudocystic cavity as previously described [32]. Each stage comprises 6 subjects, including male and females except for Stage III for which only males could be obtained. Tissue was formalin-fixed, paraffin-embedded and utilized for immunohistochemistry analysis.

### Immunohistochemical staining and quantification of human and mouse stroke samples

Formalin-fixed and paraffin-embedded (FFPE) autopsy samples from human stroke cases and mouse stroke samples were collected to investigate the overall expression level of OPN by immunohistochemistry (IHC) in human infarct core, peri-infarct region and normal appearing tissue as well as mouse infarct core, peri-infarct region and contralateral hemisphere. Briefly, paraffin-embedded tissues from both species were cut into 3 μm thin sections on a Leica microtome (Leica Microsystems, SM2000R) and were deparaffinized and rehydrated in decreasing ethanol concentration prior to stainings. Hematoxylin and Eosin (H&E) staining was performed according to standard protocol to identify stroke core and peri-infarct region in mouse samples, and to determine the histopathological grade [stages I (acute necrosis), II (macrophage resorption) and III (pseudocystic cavity)] [32] and localization of infarct core, peri-infarct region and normal appearing tissue in human specimen. Single or double staining for OPN or albumin and Podocalyxin antibodies, respectively, (Supplementary Table 4, online resource) was performed by standard protocol [26] on an automated immunohistochemistry system (Leica, Germany). For OPN staining light microscopy images were acquired using a wide-field microscope (Nikon 80i) with 20 × objective for human and 40 × objective for mouse samples, keeping exposure settings constant between specimen/samples. Albumin (pink) and Podocalyxin (brown) double stained slides were scanned using a 20 × objective at 0.22 µm/pixel (Axio Scan Z1, Zeiss) and analyzed using QuPath open source software (version 0.3.2) with equal area ROIs from different regions exported to Image J software in TIFF format (version 1.5).

OPN expression intensity in the stroke area and normal appearing tissue (NAT) in human samples was evaluated using a scoring system that includes 4 expression levels (0, no expression; 1, mild; 2, moderate; 3, severe). In addition, two images from the infarct core, peri-infarct region and normal appearing tissue per patient were acquired as nd2 files. Using the NIS-Elements software (v5, Nikon), raw.nd2 files were subjected to binary thresholding using whole image as the region of interest (ROI). Measurement was performed for binary area and mean intensity of the OPN staining. Values from each of the two images were exported to a spreadsheet (MS Office Excel). IHC expression intensity of OPN was obtained in arbitrary units (a.u.) as the product of the binary area and the mean intensity of the OPN staining within this area. The same strategy was applied for quantification of OPN expression intensity in mouse samples, with the difference that three coronal sections per sample were used and one image from infarct core, peri-infarct region and contralateral hemisphere per section was acquired as an.nd2 file. Generated data was then imported to Prism software (v6, GraphPad) for graphing and statistical analysis. For representative images, light microscopy (Nikon 80i) was performed using 4×, 20× and 40× objectives, and images were exported as high-resolution TIFF files. Acquisition of images and subsequent OPN expression analysis in human and murine stroke samples was performed by blinded neuropathologist and neurologist.

### Therapeutic rescue experiments by administration of neutralizing anti-OPN antibody

Female and male adult wild-type mice were treated randomly 4 h after tMCAO by subcutaneous (s.c.) injection 100 µL PBS containing either polyclonal goat anti-mouse OPN Ab (R&D Systems, #AF808) or non-immunized goat immunoglobulin G (R&D Systems, #AB-108-C) at a dose of 0.4 mg/kg body weight. Assessment of global neurological functions with the 14-points mNSS [16] (Supplementary Table 2, online resource) was performed by a blinded investigator 24 h post-ischemic stroke. After neurological scoring, all surviving animals were sacrificed, and brains were sectioned into 2 mm coronal sections. For each animal, three brain slices were incubated in 2% 2,3,5-triphenyltetrazolium chloride (TTC, Merck, #108380) in saline at 37 °C for 10 min in the dark and images from both anterior and posterior sides acquired with an optical scanner. TTC positive brain sections were then fixed in 4% paraformaldehyde (PFA) overnight at 4 °C, followed by paraffin embedding using standard protocol and the blocks stored at room temperature for further use in IHC. Survival benefit analysis was performed based on the ratio of surviving and dead animals in both groups 24 h post-tMCAO. Hemorrhagic transformation of ischemic lesions observed in TTC stained slices were characterized as hemorrhagic infarction (petechial infarct) or parenchymal hematoma (hemorrhage with mass effect) using adapted criteria [87]. Intraluminal filament occlusion in C57Bl/6J mice suffering from dysplasia of posterior communicating arteries leads to an occlusion duration-dependent increase in severity of cerebral hypoperfusion and extension of ischemic pathology beyond MCA territory, such as in the thalamus and the hippocampus [83]. We observed such infarctions beyond MCA territory in 6 mice form the control IgG group and 5 in the anti-OPN antibody treated group. Animals subjected to tMCAO were excluded for any further experiments if one of the following preset exclusion criteria was met: (1) no symptoms of stroke and/or no ischemic lesion visible in TTC staining (1 control and 2 anti-OPN treated animals were excluded); (2) intracranial and/or intracerebral hemorrhage due to endoperforation by the monofilament (no animals were excluded); (3) death during anaesthesia and/or surgery but not related to large infarction and/or edema (3 animals were excluded).

### Infarct and edema volume quantification

Infarct and edema volumes were assessed by a blinded neurologist using TTC stained coronal slices. For both anterior and posterior sides, contralateral and ipsilateral hemispheres and infarct areas were traced and measured using ImageJ software 1.52a. As each cerebrum was sectioned into 2 mm coronal sections, measured areas were multiplied with 1 mm thickness each, obtaining anterior and posterior contralateral and ipsilateral hemisphere and infarct volumes. Total contralateral and ipsilateral hemisphere and infarct volumes per cerebrum were finally obtained by calculating the sum of volumes across all slices. Edema-adjusted infarct volume [named as stroke volume (SV) in the current manuscript], was calculated as followed: SV (mm^3^) = infarct volume × (1 − [ipsilateral hemisphere volume − contralateral hemisphere volume)/contralateral hemisphere volume]) [90]. Edema volume was obtained by subtracting the contralateral from the ipsilateral hemisphere volume.

### Immunofluorescence staining and quantification of paraffin-embedded human and mouse stroke samples

Human stroke tissue sections were deparaffinized and rehydrated in decreasing ethanol concentration prior to immunofluorescence staining as described above and then incubated in a blocking solution (0.2 M PBS with 10% horse serum, 0.5% Triton X-100, 0.2% BSA). For immunofluorescence staining tissue sections were incubated in a carrier solution (0.2 M PBS with 1% horse serum, 0.5% Triton X-100, 0.2% BSA) containing primary or secondary antibodies (Supplementary Table 4, online resource). Immunofluorescence staining of paraffin-embedded mouse brain coronal sections was performed as described previously [25] except for lymphocyte infiltration, with modifications only for the primary and secondary antibodies used for staining (Supplementary Table 4, online resource). Images were acquired using a wide-field microscope (Nikon 80i) with 40 × objective applying identical exposure and gain settings across samples for a particular antibody combination. For lymphocyte infiltration multiplex staining, FFPE sections of mouse brains were stained using Opal Polaris 7 colour kit (NEL861001KT, Akoya Biosciences, Inc.) based on thyramide signal amplification immunostaining method. Antigen retrieval was performed with the AR9 buffer (pH9, Akoya Biosciences, Inc., AR900250). Multiplex stainings were performed on LabSat™ Research Automated Staining Instrument (Lunaphore Technologies SA). Images were acquired on Vectra Polaris (Akoya Biosciences, Inc.) using MOTiF™ technology at 0.5 µm/pixel.

For quantification of expression intensity in mice, three coronal sections per sample were used and one image from stroke core, peri-infarct region and contralateral hemisphere per section were acquired as nd2 files. For human stroke samples, two images from the infarct core, peri-infarct region and normal appearing tissue per patient were acquired. Immunofluorescence cell number and expression intensities were obtained as described above with minor modifications: measurement was performed for binary area and mean fluorescence intensity applying 12 × smooth function, 2 × separation. Cell type-specific and astrocyte endfeet OPN expression intensity analysis was performed from overlapping binary area between corresponding channels using intersection function in the software. Values from each image were exported to a spreadsheet (MS Office). Immunofluorescence expression intensity was obtained in arbitrary units (a.u.) as the product of binary area and the mean fluorescence intensity within this binary area. Immunofluorescence positive cell number was counted manually. Cell type-specific marker or OPN expression intensity was obtained as the product of overlapping binary area between channels with the mean intensity of channel of interest. For vessel-associated microglia/macrophages analysis, regions of interest (ROI) were drawn around microglia/macrophages associated with the vessels followed by quantification as above for expression intensity within these ROI followed by summation of all ROI for each image. Results were imported to Prism software (v6, GraphPad) for graphing and statistical analysis. For representative images, immunofluorescence microscopy (wide-field—Nikon 80i and confocal—Nikon A1) was performed using 20 × and 40 ×, and 100 × objectives, and images were exported as high-resolution Nd2 files and as TIFF files. Acquisition of images and subsequent expression analyses were performed by a blinded neurologist.

### Isolation and culture of primary brain endothelial cells

Primary brain microvascular endothelial cells were isolated from murine brains exactly as described previously [41], whereas with minor modifications for porcine brain endothelial cells. Briefly, the isolated pig brains were immersed in 10 mM penicillin–streptomycin solution (Gibco, #15140148) in buffer A on ice for 30 min followed by removal of cerebellum and meninges. The cortices were homogenized and further digested with 0.75% collagenase II (Worthington, 3:1 homogenate to enzyme) for 1 h at 37 °C with rigorous shaking followed by a centrifugation in 25% BSA/PBS at 2000*g*, 4 °C to remove myelin. The microvessel pellet obtained was further digested in 0.5 mg/mL collagenase/dispase (Roche) and 0.5 µg/mL DNAse1 (Worthington) for 15 min at 37 °C followed by resuspending and plating the cells in MCDB-131 complete medium. Cells from one adult porcine brain were plated onto four T75 flasks coated with rat tail collagen I (Corning, #354236) and were cultured for 3–5 days in vitro until 80% confluency as described previously for mouse brain endothelial cells [25]. Cells were trypsinized and frozen down at this P0 primary passage in FBS containing 10% DMSO.

### Transendothelial electrical resistance (TEER) measurements

Frozen porcine brain microvascular endothelial cells (PBMEC, P0) were thawed or mouse brain microvascular endothelial cells (MBMEC) were plated onto 24-well transwell inserts (Greiner Bio-One, #662610) coated with fibronectin (5 µg/cm^2^, Sigma, #F1141) at 100,000 cells/cm^2^ density in MCDB-131 complete medium [25]. Transwell inserts were transferred to CellZscope device (nanoAnalytics) within 2–3 h after endothelial plating and impedance measurements were initiated as described previously [20]. After the cells reached a plateau in transendothelial electrical resistance (TEER) values indicating a mature BBB in vitro, medium was changed to serum-free medium (MCDB-131, 2 mM penicillin–streptomycin, 2 mM l-glutamine) comprising the treatment compounds. For osteopontin treatment, PBMECs were treated in both apical and basal chambers either with 0.5 µg/mL murine recombinant protein (R&D Systems, #441-OP-050) or with the vehicle 0.0005% BSA/PBS using quadruplicate inserts for each condition. Measurements were recorded for 2–3 days. Values before the treatment were set to 100% within the cellZscope software to account for electrode disturbances within replicate inserts. For the 12, 24 and 48 h timepoints’ analysis, recombinant OPN condition data were expressed as a percentage of their respective control at each timepoint. To obtain the consolidated TEER data, three different experiments (corresponding to three independent PBMEC preparations) were combined.

### Immunostaining of primary brain endothelial cells

Primary PBMEC or MBMEC seeded on transwell inserts were used for immunostaining of adherens and tight junction molecules as well as for OPN staining. For Claudin5 and VE-Cadherin cells were fixed in ice-cold methanol (3 min), whereas in 4% PFA (10 min) for CD31 and OPN staining, all at room temperature followed by washes in cold PBS. Cells on the insert were then permeabilized in PBS containing 0.5% BSA, 0.2% Triton X-100 (used in all steps unless otherwise indicated) for 45 min, followed by 1 h at room temperature with primary antibodies (Supplementary Table 4). Washes were performed followed by 1 h incubation at room temperature in the dark with fluorescent conjugated secondary antibodies and DAPI (1/500). After washes in PBS, inserts membranes were cut and mounted with Aqua Polymount. Imaging was performed using Nikon 80i widefield microscope (40 × objective) and images exported in TIFF format.

### OPN and anti-OPN IgG treatment of MBMEC

Mouse brain microvascular endothelial cells (MBMEC) at 3 day post-isolation were seeded in 24-well plates at passage 1. These cells were treated with murine recombinant OPN (R&D Systems) with or without anti-OPN IgG (R&D systems) at the indicated concentrations at 48 h post-plating when the cells reached confluency. The treatment period was for 24 h followed by harvesting the cells to obtain RNA and cDNA exactly as described above using RNeasy micro kit (Qiagen) followed by cDNA synthesis (Revertaid kit, Thermofisher). qRT–PCR was performed using Absolute qPCR SYBR Green Fluorescein Mix in IQ5 instrument (Biorad) using a previously established PCR protocol [25, 41]. *Rplp0* was used as a house-keeping gene and qRT–PCR was performed for *Cd44* and *Mmp12* using 2^−ΔΔCt^ method. Primers are listed in Supplementary Table 1, online resource.

### Oxygen–glucose deprivation (OGD) in vitro model

For oxygen–glucose deprivation (OGD) model, MBMECs were seeded on inserts as described above and cultured in complete medium until they reach the plateau phase. OGD model was induced by changing the MBMEC medium to serum and glucose free DMEM basal medium (Gibco, #A14430) followed by transferring the inserts to the CellZscope device placed an hypoxic incubator (1% O_2_) for 24 h. Control cultures were performed in serum-free DMEM basal medium containing 5.6 mM glucose and the inserts transferred to a CellZscope device placed in a normoxic incubator (19.5% O_2_). Cells were treated either with control isotype or anti-OPN antibody (3 µg/mL) for 24 h of the OGD condition or just with control isotype for the normoxic condition. At the endpoint, cells on the inserts were further used for RT–qPCR, immunofluorescence or permeability assays.

### In vitro permeability assay

Fluorescently labeled tracers 0.45kD Lucifer yellow (LY, Sigma, #L0144), 3kD Texas Red^®^ Dextran (TxR 3kD, Thermofisher, #D3328), 20kD tetramethylrhodamine isothiocyanate dextran (TMR 20kD, Sigma, #73766) in a final concentration of 10 μM, whereas alexa 647 conjugated-albumin (al647-albumin, Thermofisher, #A34785) and 70kD Fluorescein isothiocyanate dextran (FITC 70kD, Sigma, #FD70S) in a final concentration of 5 μM were added to the top chamber of the filter inserts previously used for impedance measurement. Samples were taken from the bottom chamber after 2 h of incubation. Fluorescence was analyzed using the following order of excitation/emission (nm): al647-albumin 645/675, TxR 3kD 595/625, TMR 20kD 550/580, FITC 70kD 490/520, LY 425/525. Permeability flux was calculated as a ratio of bottom to top chamber fluorescence (pe index = *B*/*T*) once corrected for autofluorescence background [40, 43].

### Statistical analysis

Data are represented as histogram bars or dot plots with underlying bar graphs showing mean ± SEM (standard error of the mean). The statistical details for each experiment can be found in the figure legend (sample size, testing and *P* values). A *P* value < 0.05 was considered statistically significant and indicated by the following symbols: */§/† for *P* < 0.05; **/§§/†† for *P* < 0.01; ***/§§§/†††/$$$ for *P* < 0.001 and ****/§§§§/††††/$$$$ for *P* < 0.0001. Statistical analyses were performed using GraphPad Prism software (v6, GraphPad).

### Data availability

Raw data were generated at the Edinger Institute (Institute of Neurology) Frankfurt/Main. Derived data supporting the findings of this study are available from the corresponding author on reasonable request. The complete RNAseq data set (NCBI GEO ID GSE163752) is accessible freely at https://bioinformatics.mpi-bn.mpg.de/SGD_Stroke in an interactive manner. The related UpSet analysis can be visualized at: http://vcg.github.io/upset/ followed by submitting the JSON file link (https://raw.githubusercontent.com/SGD2020/mcao/master/mcao.json) in the load data address bar. The.CSV text file containing binary data including mean expression reads and Log2FC for all the cell types for both contralateral and ischemic hemispheres can be downloaded (right click) from the GitHub repository (https://github.com/SGD2020/mcao/blob/master/12csvnew.csv).

## Results

### EPAM-ia method generates a high yield of substantially pure NVU cells from murine ischemic brain tissue

For simultaneous analysis of the response of multiple NVU cell types to cerebral ischemia and evaluation of its impact on BBB function we developed the EPAM-ia method. This method is based on differential but simultaneous processing of the major NVU cell types (endothelial cells, EC; pericytes, PC; astrocytes, AC; and microglia, MG) from the same tissue by employing mechanical homogenization, filtration and enzymes specific for each cell type followed by fluorescence-activated cell sorting of NVU cells (Supplementary Fig. 1a, online resource). The success of this advanced method was first evaluated with a proof of concept in healthy adult WT mice (Supplementary Fig. 1a, online resource). It is well known that astrocytes and microglia require mild enzymatic and mechanical dissociation, whereas vascular cells (endothelial cells and pericytes) need harsh homogenization and enzymatic digestion to detach them from the basal lamina. For detachment of astrocytes and microglia from the surrounding tissue, the initial step is based on dissociation using fine mincing and mild enzymatic digestion using papain and DNase-I according to previous methods described for isolation of neural cells [36, 129]. To further isolate endothelial cells and pericytes without affecting astrocytes and microglia, the sample was filtered through a 10 µm mesh, a critical step to separate the vessel fragments from glial and other neural cells. While the flow-through containing glial cells was stored on ice, the vessel fragments from the top of the mesh were subjected to tight-fitting dounce homogenization and harsh enzymatic digestion steps with collagenase and dispase, used for the primary brain endothelial cultures [41, 74]. After myelin removal and the last vessel fragments digestion step, both the glial and vascular cell suspensions were combined back together for subsequent immunolabeling. Endothelial cells, pericytes, astrocytes and microglia were sorted by flow cytometry utilizing established markers for each cell type (Supplementary Fig. 1b, online resource). Astrocyte Cell Surface Antigen 2 (ACSA2), encoded by the *Atp1b2* gene, was used to isolate astrocytes, in which it is highly expressed [62]. A combination of two established markers, platelet derived growth factor receptor beta (PDGFRβ) and chondroitin sulfate proteoglycan-4 (NG2/CSPG4), was chosen to detect pericytes [46, 111], and vascular endothelial cadherin (VECAD)[24] was included for endothelial cells selection. To remove the cells of interest that are clumped with blood cells, the cluster of differentiation-45 (CD45) was additionally introduced into our gating strategy as an exclusion marker [120]. This was also used in combination with CD11b for isolation of microglia as CD45^low^/CD11b^pos^ cells [6]. Moreover, these markers were not only used to select cells of interest but also included as negative markers in the gating strategy to ensure that each targeted cell type is not contaminated by the others (e.g., for astrocytes: CD45^neg^/ACSA2^high^/NG2^neg^/PDGFRβ^neg^/VECAD^neg^; Supplementary Fig. 1b. online resource). Applying the EPAM-ia method, which includes the tissue dissociation procedure (Supplementary Fig. 1a. online resource) and FACS strategy (Supplementary Fig. 1b. online resource), allowed us to obtain high yields of astrocytes, microglia, endothelial cells and more importantly pericytes from WT adults animals (Supplementary Fig. 1c, online resource). The overall cell numbers per brain (1.5 × 10^6^ per brain) determined using a hemocytometer were similar to those obtained by FACS and were consistent across preparations. The amount and the quality of RNA isolated from these cells was suitable for RNA sequencing (RIN values > 7; Supplementary Fig. 1d, online resource). Moreover, a significant enrichment of NVU cells was confirmed by qRT–PCR with cadherin 5 (*Cdh5*) and solute carrier family 1 member 1 (*Slc1a1*) for enodothelial cells (Supplementary Fig. 1e, online resource), potassium voltage-gated channel subfamily member 8 (*Kcnj8*) for pericytes (Supplementary Fig. 1f, online resource), aquaporin 4 (*Aqp4*) and solute carrier family 1 member 3 (*Slc1a3*) for astrocytes (Supplementary Fig. 1g, online resource) and transmembrane protein 119 (*Tmem119*) for microglia (Supplementary Fig. 1 h, online resource).

**Fig. 1:**
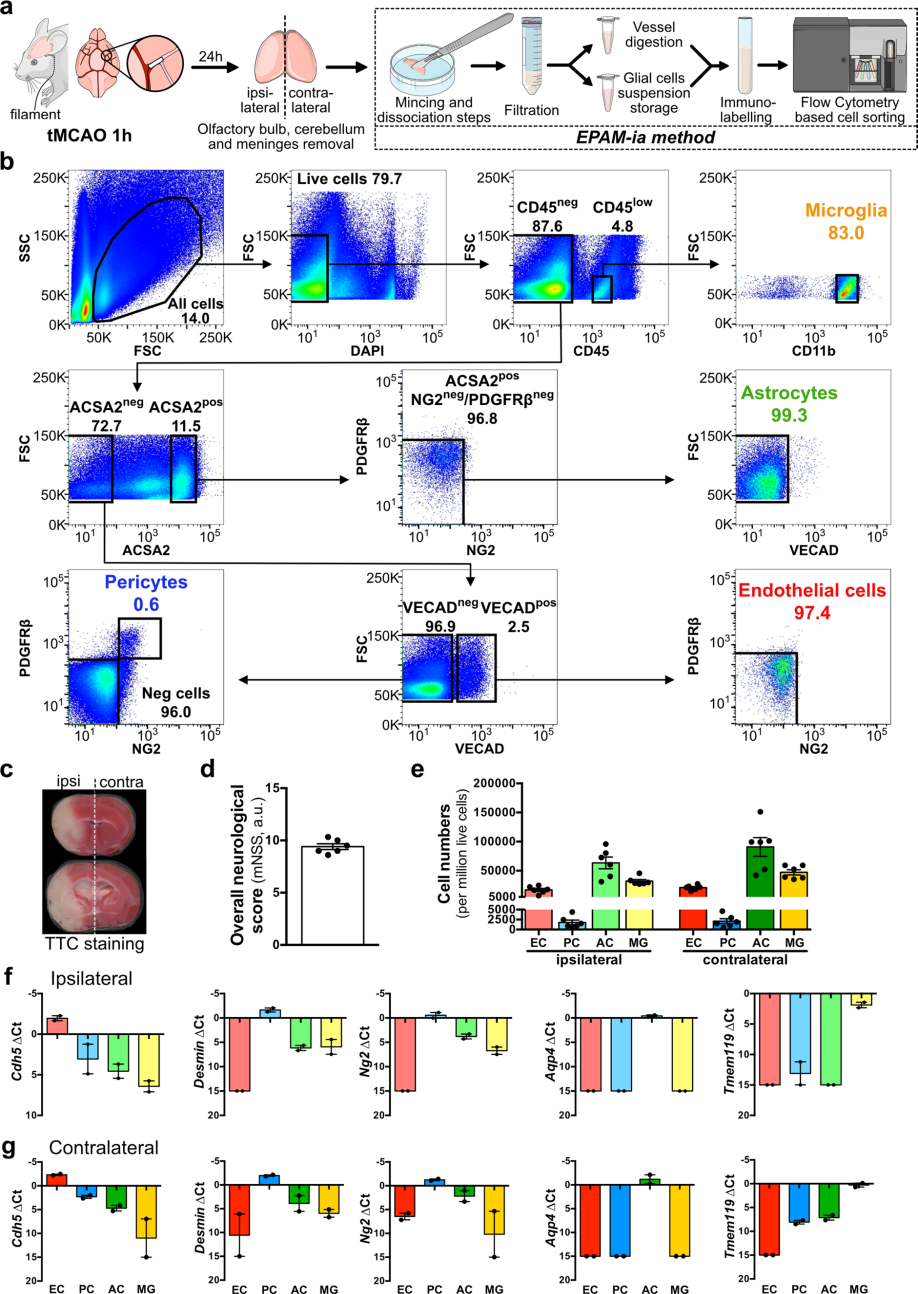
Application of the EPAM-ia method to a murine model of ischemic stroke. **a** Schematic illustration of the transient middle cerebral artery occlusion (tMCAO) procedure followed by isolation of NVU cells applying the EPAM-ia method 24 h post-stroke. **b** Gating strategy allowing the simultaneous separation and collection of endothelial cells, pericytes, astrocytes and microglia from the ischemic stroke hemisphere. First FSC/SSC plot shows 1,000,000 events. **c** Visualization of damaged brain areas after tMCAO using 2,3,5-triphenyltetrazolium chloride (TTC) staining. **d** Average neurological scores of biological replicates used for NVU transcriptomic study 24 h post-ischemic stroke using a 14-points modified Neurological Severity Score (mNSS) (*n* = 6, 3–4 mice pooled per biological replicate). **e** Normalized cell numbers of endothelial cells (EC), pericytes (PC), astrocytes (AC) and microglia (MG) isolated from ipsilateral (ischemic) and contralateral (control) hemispheres with the EPAM-ia method (*n* = 6, 3–4 mice/preparation). **f**, **g** Purity of sorted NVU cells obtained from ipsilateral (**f**) and contralateral hemispheres (**g**) was assessed by qRT-PCR targeting cell type-specific markers for endothelial cells (*Cdh5*), pericytes (*Desmin* and *Ng2*), astrocytes (*Aqp4*) and microglia (*Tmem119*), *n* = 2. If no amplification was detected, the ΔCt value was set at 15 by default

For application of the EPAM-ia method to ischemic stroke, adult WT male mice were subjected to tMCAO and their global neurological functions were assessed 24 h post-stroke before sacrificing the mice (Fig. 1a). Ipsilateral (ischemic) and contralateral (control) hemispheres were subsequently used for simultaneous flow cytometry-based isolation of endothelial cells, pericytes, astrocytes and microglia from the same tissue applying the EPAM-ia method (ipsilateral hemisphere, Fig. 1b; contralateral hemisphere and unstained control, Supplementary Fig. 2, online resource). Neurological scoring was used to confirm the presence of stroke for all animals, whereas TTC staining was performed for few selected animals from the same cohort but not included in the transcriptomic study (Fig. 1c, d). This was done to exclude interference of TTC staining with the subsequent isolation and analysis of the stroke tissue. Only the mice showing the classic neurological symptoms of stroke and having an mNSS score of at least 8 were used for subsequent NVU cell isolation (Fig. 1d). Using the EPAM-ia method, high yields of viable endothelial cells, pericytes, astrocytes and microglia were obtained from both ipsilateral and contralateral hemispheres pooling tissues from 3 to 4 animals for each isolation (Fig. 1e) and were almost comparable with those obtained from healthy brain tissue (Supplementary Fig. 1c, online resource). The purity of isolated NVU cells from ipsilateral and contralateral samples was confirmed by qRT–PCR analysis using established markers for each cell type (Fig. 1f, g). Six biological replicates (3–4 animals pooled per replicate) were collected and further subjected to bulk RNA-Sequencing.

### EPAM-ia method enables comprehensive profiling of the NVU transcriptome in murine ischemic stroke

**Fig. 2:**
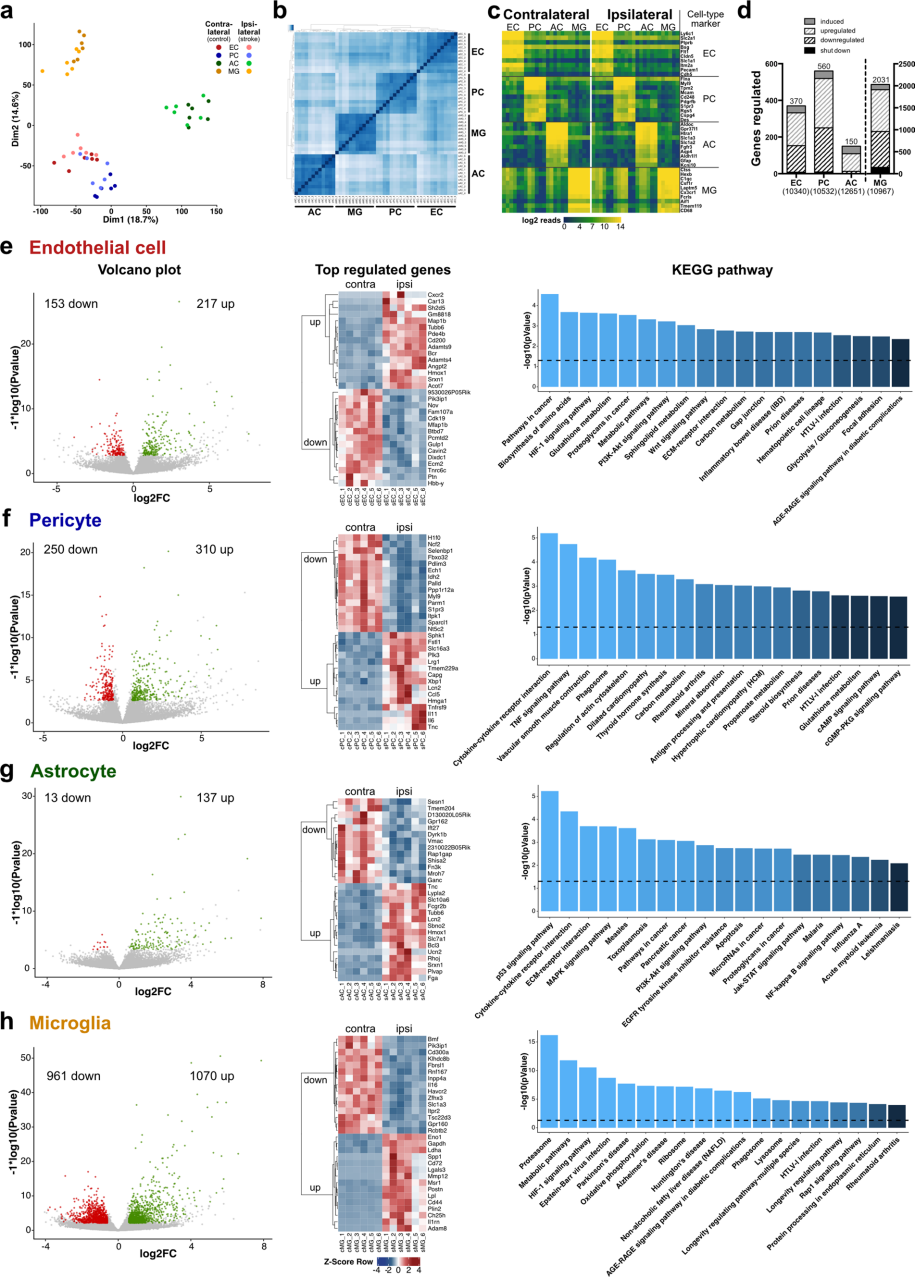
Bulk RNA-sequencing analysis of NVU cells isolated from the murine ischemic brain. **a** RNA-sequencing principal component analysis (PCA) plot of isolated NVU cells including endothelial cells (EC, red), pericytes (PC, blue), astrocytes (AC, green) and microglia (MG, yellow) from ischemic ipsilateral (light dots) and control contralateral (dark dots) hemispheres (*n* = 6, 3–4 mice/preparation). **b** Hierarchical clustering of endothelial cells, pericytes, astrocytes and microglia isolated from murine ipsilateral and contralateral hemispheres. **c** Heatmap representing log2 expression data of selected cell-specific marker genes for endothelial cells, pericytes, astrocytes and microglia from ipsilateral and contralateral hemispheres. **d** Overall number of genes expressed and shut down, downregulated, upregulated and induced in NVU cells obtained from cerebra of animals subjected to tMCAO. The total number of genes regulated is indicated on the top of histograms. The total number of genes expressed in each of the individual cell types is indicated below the histogram. **e**–**h**, Volcano plots (left) showing significantly downregulated (red) and upregulated (green) genes with corresponding heatmaps (middle) representing top 15 downregulated and 15 upregulated genes in endothelial cells (**e**), pericytes (**f**), astrocytes (**g**) and microglia (**h**), respectively. Regulated genes were further analysed to reveal significantly regulated KEGG pathways (right, dotted line indicates the cut-off value for significance). The interactive transcriptomic dataset of NVU cells in murine stroke is available at https://bioinformatics.mpi-bn.mpg.de/SGD_Stroke

Currently, simultaneous isolation and analysis of NVU cells from the same CNS tissue in health and disease is limited to a maximum of two NVU cell types (references summarized in Supplementary Table 5). The EPAM-ia method presented here facilitates the syngenic isolation of multiple and substantially pure NVU cell populations, paving the way for comprehensive profiling of the NVU transcriptome in ischemic stroke (NCBI GEO ID GSE163752; https://bioinformatics.mpi-bn.mpg.de/SGD_Stroke). Post-stroke, gene expression PCA plot (Fig. 2a and Supplementary Fig. 3, online resource) and hierarchical clustering analysis (Fig. 2b) revealed well separated endothelial cells, pericytes, astrocytes and microglia gene clusters indicative of highly efficient cell separation and low contamination. Moreover, well-known cell type-specific markers exhibited high expression levels in their corresponding cell types and were undetectable or with extremely low expression levels in the other cell populations from both ipsilateral and contralateral brain samples (Fig. 2c). These markers include, among others, *Cldn5*, *Pecam1*, *Ptprb* and *Cdh5* for endothelial cells; *Pdgfrb*, *Des*, *Cd248* and *Cspg4* for pericytes: *Gfap*, *Aldh1l1*, *Slc1a3* and *Aqp4* for astrocytes; and *Aif1*, *Tmem119*, *Cd68* and *Csf1r* for microglia [6, 111]. Moreover, neuronal markers were not detectable in most of the biological replicates and cell types (Supplementary Fig. 3e, online resource) These data confirmed the purity of the isolated NVU cell types and established the feasibility of constructing a high-quality transcriptome database of NVU cells in acute ischemic stroke.

We first considered each cell type independently and compared differentially expressed genes between ipsilateral and contralateral hemisphere using gene set enrichment analysis (GSEA) [105]. In total, 370 genes were regulated during ischemic stroke in endothelial cells, 560 in pericytes, 150 in astrocytes and 2031 in microglia (Fig. 2d). In detail, 153 genes were downregulated and 217 genes upregulated in endothelial cells, 250 genes downregulated and 310 genes upregulated in pericytes, 13 genes downregulated and 137 genes upregulated in astrocytes, and 961 genes downregulated and 1070 genes upregulated in microglia as depicted in the respective volcano plots (left panels; Fig. 2e–h). The top 15 downregulated and 15 upregulated genes of all four NVU cell types (based on significance) are shown in the corresponding heatmaps (middle panels; Fig. 2e–h). Among these genes, there were several novel candidates, that have not been reported in stroke, and few that have been described to be regulated in in vivo models of ischemic stroke. The latter include genes encoding modulators of the extracellular matrix, such as extracellular proteases *Adamts4* and *Adamts9* in endothelial cells [89] and *Mmp12 *[15] in microglia or the regulator of CNS angiogenesis *Angpt2* in endothelial cells [41]. Also, many genes encoding chemokines, cytokines and chemokine receptors, which are involved in neuroinflammatory processes in ischemic stroke, were regulated, including *Cxcr2* [47] in endothelial cells, *Ccl5* [34], *Il11* and *Il6* [117] in pericytes, *Lcn2* [57] in astrocytes [124] and pericytes, and *Il1rn* [126] in microglia. Bioinformatic pathway analyses using KOBAS [121] revealed the involvement of several pathways in NVU cells, of which some have been reported to be involved in stroke pathophysiology: HIF-1 signaling pathway in microglia [8] and in endothelial cells, Wnt signaling pathway and PI3K–Akt signaling pathway in endothelial cells [65, 101], ECM receptor interaction in endothelial cells [65] and astrocytes [124], cytokine–cytokine receptor interaction [66] in astrocytes and pericytes, TNF signaling pathway [66] in pericytes, and Jak-STAT, NF-κB and p53 signaling pathway in astrocytes [60, 66, 107, 124] (right panels; Fig. 2e–h).

### UpSet analysis reveals osteopontin as a potential therapeutic target in acute ischemic stroke

**Fig. 3:**
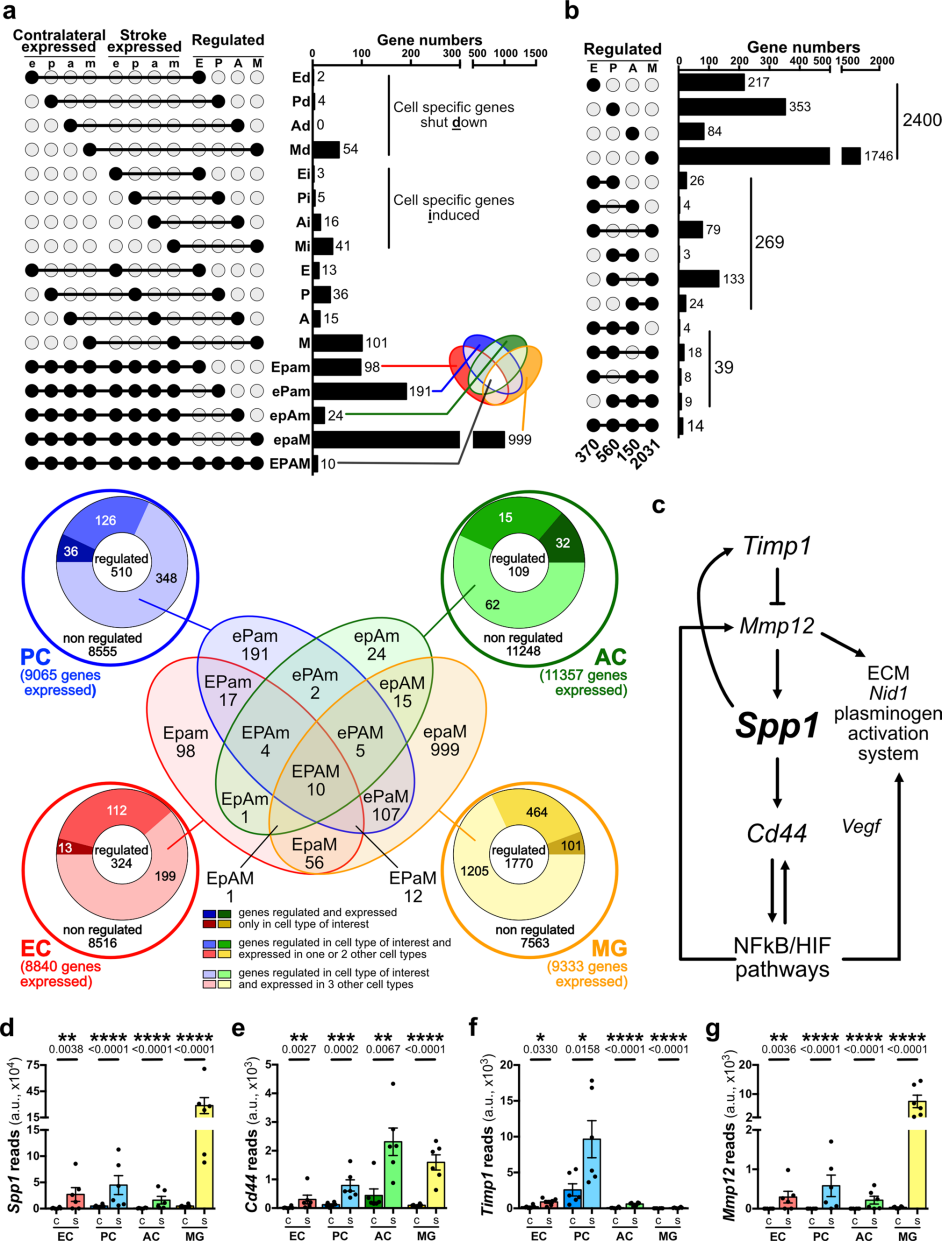
UpSet analysis of the NVU transcriptome reveals osteopontin as a potential therapeutic target in stroke. (**a**, top) UpSet plots demonstrating selected gene sets and their intersections (upper left) as well as corresponding genes numbers (upper right) revealing genes that are cell-specific shut down (expressed in contralateral hemisphere and regulated in one cell type only: Ed, Pd, Ad, Md), or induced (expressed only in ipsilateral hemisphere and regulated in only one cell type: Ei, Pi, Ai, Mi), or expressed in both hemispheres and regulated in only one cell type (E, P, A, M), or expressed in all four cell types of both hemispheres and either regulated in only one (Epam, ePam, epAm, epaM) or genes expressed and regulated in all four NVU cell types (EPAM). Lowercase letters (e, p, a, m) indicate expression gene sets and uppercase letters indicate regulated gene sets (E, P, A, M). (**a**, bottom) Venn diagram showing overlaps of differently regulated genes (expressed in all four cell types) and surrounded by pie charts giving the overall cell-specific number of expressed genes including non-regulated and regulated genes. **b** UpSet plots of gene sets (left) and corresponding genes numbers (right) regulated in one, two, three or four NVU cell types without considering the expression in the contralateral and/or ipsilateral hemispheres. **c** Interaction pathway of secreted phosphoprotein 1 (*Spp1*, encoding for osteopontin) and its signaling pathway members *Cd44* (EiPiAM), *Timp1* (EPAMi) and *Mmp12*^‡^ (EiPiAiM). These *Spp1* pathway members were revealed by the UpSet analysis to be differentially regulated in all the NVU cell types after acute ischemic stroke in mice. **d**, **g** Visualization of actual base reads of gene expression by NVU transcriptome profiling shows upregulation of *Spp1* (**d**) *Cd44* (**e**), *Timp1* (**f**) and *Mmp12* (**g**). ^‡^It is of note that even though the Log2FC and the *P* value of *Mmp12* fulfils the criteria to be defined as a gene regulated in all NVU cell types in our original transcriptomic analysis (**g**), *Mmp12* will appear in the Mi intersection in the stricter UpSet representation. This is because the number of reads for *Mmp12* was less than 10 in one out of six biological replicates for pericytes and astrocytes, and less than 10 in two out of six biological replicates for endothelial cells in the ipsilateral hemisphere, and also less than 10 in one out of six biological replicates for microglia in the contralateral hemisphere. *n* = 6, 3–4 mice/preparation, *P < 0.05, **P < 0.01, ***P < 0.001 and ****P < 0.0001 determined by DESeq2 with Benjamini–Hochberg correction

As BBB function is regulated by all the major NVU cell types, we performed the UpSet analysis [72] to visualize commonly regulated genes in the NVU cell types. Therefore, the gene expression and regulation data of each cell type post-stroke (Fig. 2) were transformed into a binary format and uploaded to the UpSet server for public exploration ((http://vcg.github.io/upset/) followed by pasting the JSON file link (https://raw.githubusercontent.com/SGD2020/mcao/master/mcao.json) in the load data address bar) as described in the methods. This led to 12 binary data sets reflecting expression in contralateral hemisphere, expression in ipsilateral ischemic hemisphere, and gene regulation between the two hemispheres for each cell type (e, p, a, m in contralateral expressed; e, p, a, m in stroke expressed; E, P, A, M in regulated, respectively; Fig. 3a). Prominent intersections for NVU cells in acute ischemic stroke have been exemplarily displayed as an UpSet plot with intersections indicated by a dark line and intersecting sets as dark circles, and with the number of genes in a particular intersection in the corresponding bar plots (Fig. 3a, upper panel). In this, we show genes shutdown (Ed, Pd, Ad, Md), genes induced (Ei, Pi, Ai. Mi), and genes expressed and regulated (E, P, A, M) in single cell types. Moreover, genes expressed in all cell types but regulated in only one cell type (Epam, ePam, epAm, epaM) are represented along with genes expressed and regulated in all four cell types (EPAM). Some of these intersections are also indicated in the classic Venn diagrams displaying intersections of genes expressed in all four cell types in both hemispheres, and additionally regulated in at least one cell type (Fig. 3a, lower panel). However, we concentrated on UpSet plots highlighting only the regulated genes from each cell type (370 in endothelial cells, 560 in pericytes, 150 in astrocytes, and 2031 in microglia) independently of their expression in contralateral or ipsilateral hemisphere to identify the intersecting sets relevant in stroke (Fig. 3b). Upset analysis revealed genes that are regulated in cell specific or multicellular fashion. Interestingly, we detected an intersection of 14 genes (Fig. 3b) that were commonly regulated post-stroke in all four cell types, of which 10 genes were expressed in both hemispheres and in all the cell types (EPAM; Fig. 3a). Detailed analysis of these genes and their pathways (string database https://string-db.org, reactome database https://reactome.org/PathwayBrowser/) led to *Spp1*, which encodes for osteopontin [98], as a potential target that was upregulated in all four NVU cell types (EPAM) post-stroke (Fig. 3c, d). Moreover, several genes critically associated with the *Spp1* signaling pathway are also regulated in all NVU cell types (Fig. 3c). This includes *Cd44* (EiPiAM, Fig. 3e) [116], *Timp1* (EPAMi, Fig. 3f) and *Mmp12* (EiPiAiM, Fig. 3g). Concomitant upregulation of all these genes in the osteopontin signaling pathway and in all the NVU cell types may have critical implications for BBB function in ischemic stroke suggesting osteopontin as a therapeutic target in this disease.

### Osteopontin is highly expressed in brain tissue of ischemic stroke patients

**Fig. 4:**
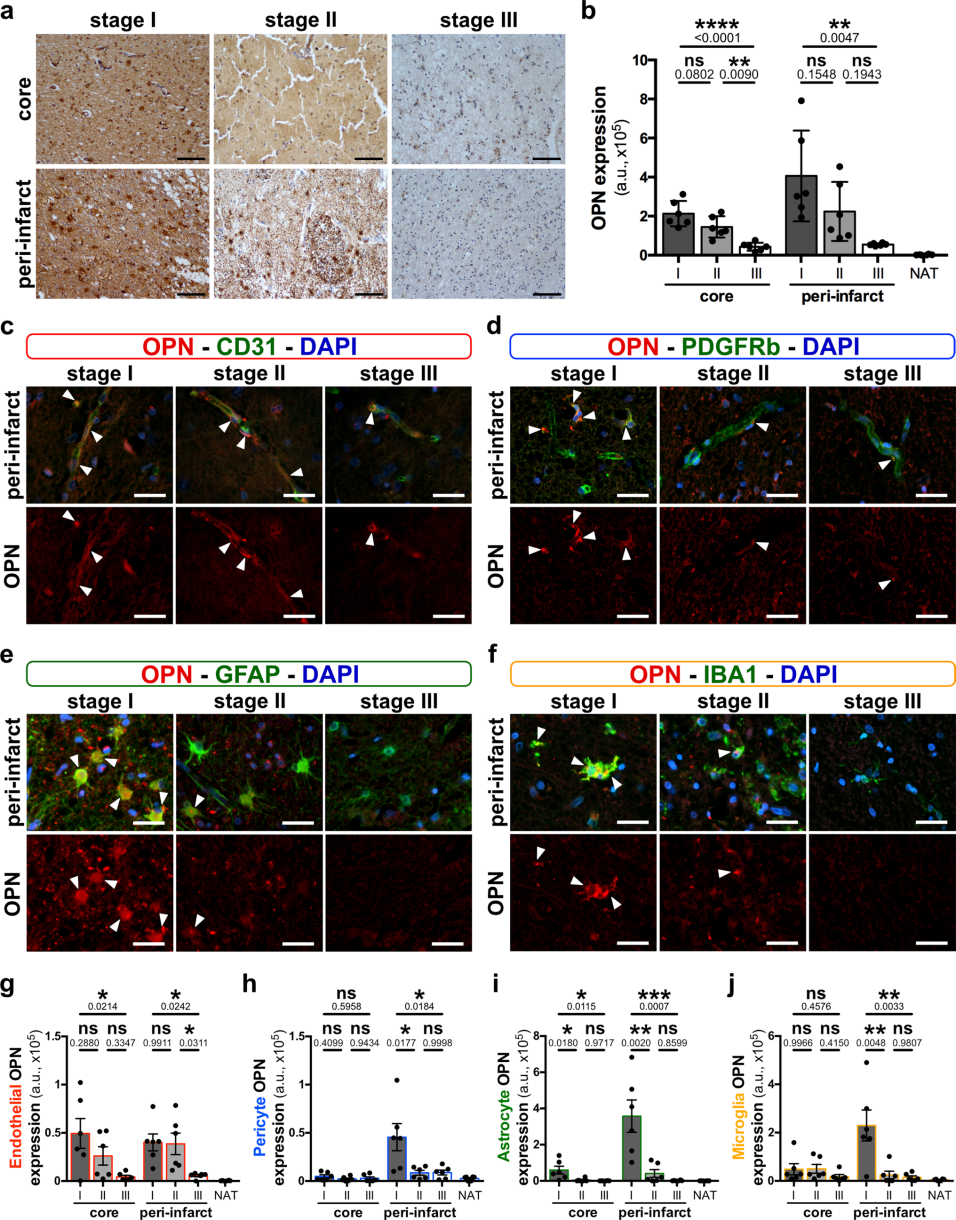
Osteopontin expression in normal and ischemic brain tissue of stroke patients. **a** Representative immunohistochemistry staining for osteopontin (OPN, brown) on human stroke samples at different stages (stage I–III) in the peri-infarct region and infarct core tissue. Stage I tissues were obtained 24–48 h post-vessel occlusion and present acute necrosis. Stage II is defined by macrophage resorption and stage III by the observation of pseudocystic cavity. **b** Quantification of OPN expression intensity (arbitrary unit, a.u.) in the infarct core, peri-infarct region and normal appearing tissue (NAT) at stages I–III; *n* = 6 individual specimens for each stage, **P < 0.01, ****P < 0.0001 and not significant (ns) *P* > 0.05 by one-way analysis of variance and Tukey’s multiple comparison test. **c**–**f** Representative images of immunofluorescence staining for OPN (red) and cell-specific markers (green) including CD31 for endothelial cells (**c**), PDGFRβ for pericytes (**d**), GFAP for astrocytes (**e**) and IBA1 for microglia/macrophages (**f**) in the peri-infarct regions. (**g**–**j**) Quantification of OPN expression in core, peri-infarct and normal appearing tissue endothelial cells (**g**), pericytes (**h**), astrocytes (**i**) and microglia/macrophages (**j**) at stages I–III, *n* = 6 individual specimens for each stage, *P < 0.05, **P < 0.01, ***P < 0.001, ****P < 0.0001 and ns *P* > 0.05 by one-way analysis of variance and Tukey’s multiple comparison test. Scale bars: 100 µm (**a**) and, 20 μm (**c**–**f**)

We next examined osteopontin translational relevance in human samples of stroke (Supplementary Table 3, online resource). In detail, the two major zones within the ischemic cerebrovascular bed including the peri-infarct region and infarct core were histologically differentiated from normal appearing tissue (NAT) using H&E staining (Supplementary Fig. 4a upper panel, online resource). OPN expression was assessed by IHC (Fig. 4a and Supplementary Fig. 4a lower panel, online resource) at different stages post-stroke both by neuropathological scoring (Supplementary Fig. 4b, online resource) and expression intensity analysis (Fig. 4b). While OPN was only slightly detectable in normal appearing tissue, its expression was significantly increased in peri-infarct region and infarct core in all patients (Fig. 4b and Supplementary Fig. 4b, online resource). Interestingly, the intensity correlated with the stages that are frequently used in neuropathological stroke diagnostics [32]. OPN expression in the peri-infarct region and infarct core was higher in stage I (acute necrosis) and II (macrophage resorption) that are both characterized by BBB leakage, and returned to low expression levels in stage III (pseudocystic cavity) (Fig. 4b and Supplementary Fig. 4b, online resource). Immunofluorescence co-staining confirm at the cell type level the increase of OPN expression during stage I and II in the endothelial cells (Fig. 4c, g and Supplementary Fig. 4c, online resource), and a peak in the peri-infarct at the stage I for pericytes (Fig. 4d, h), astrocytes (Fig. 4e, i) and microglia/macrophages (Fig. 4f, j). It is of note that the expression of vascular markers (CD31 and PDGFRβ) remains stable with stroke stage within the same region (Supplementary Fig. 4h, i, online resource). Glial activation is observed in peri-infarct at stage I for astrocytes (Supplementary Fig. 4j, online resource) and in core and peri-infarct for microglia/macrophages at stages I and II (Supplementary Fig. 4k, online resource). However, OPN is not detected in normal appearing tissue (Supplementary Fig. 4g, online resource). Glial activation and OPN expression was also observed specifically in astrocytes and microglia/macrophages associated with vessels (Supplementary Fig. 5, online resource). The specificity of the primary antibody was verified in stage II samples (Supplementary Fig. 4l, online resource). We further performed analysis of OPN expression with age and gender of the stroke patients. We observed a negative correlation of OPN expression with age in stage II stroke that changed to a trend towards positive correlation in stage III in the ischemic core (Supplementary Fig. 6, online resource). Based on this correlation, we speculate a role of OPN in age related stroke recovery; however, the precise mechanisms are unclear and need further study. The gender repartitioning suggested no effect on the OPN analysis in ischemic stroke (Supplementary Fig. 7, online resource); however, these data are inconclusive due to low sample sizes**.**

Overall, the above observations suggest a potential role of OPN at the NVU/BBB.

### Anti-OPN antibody treatment after acute ischemic stroke neutralizes OPN and improves outcome in mice

**Fig. 5:**
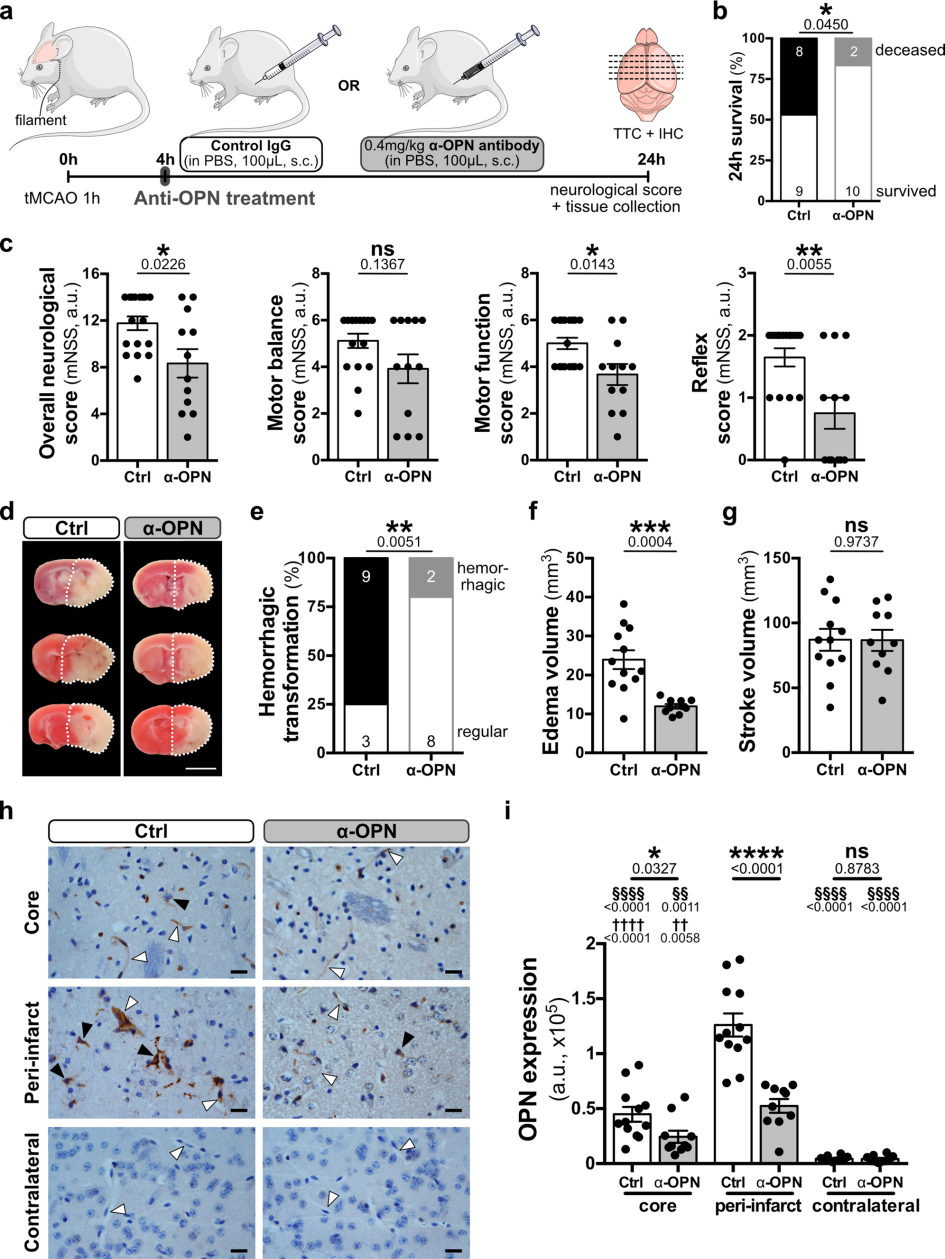
Neutralization of OPN improves clinical outcome in mice after acute ischemic stroke. **a** Schematic illustration depicting antibody treatment of mice 4 h after tMCAO with either 0.4 mg/kg control IgG or anti-OPN antibody (α-OPN) injected subcutaneously (s.c.). **b**–**i** Outcome parameters assessed 24 h after tMCAO in mice treated with Ctrl IgG or α-OPN antibody. All animals that passed the exclusion criteria were included for the survival and neurological score analysis. For analysis of hemorrhagic transformation, edema and stroke volumes, and OPN expression only mice that survived 24 h were included. **b** 24 h survival proportion with numbers in histograms indicating animals that died or survived in each group; *n* = 17 (Ctrl; 11 males and 6 females) and *n* = 12 (α-OPN; 8 males and 4 females), *P < 0.05 by one-tailed Chi-square test. **c** Total mNSS including motor balance, motor function and reflexes scores; *n* = 17 (Ctrl) and *n* = 12 (α-OPN), *P < 0.05, **P < 0.01, and not significant (ns) *P* > 0.05 by Mann Whitney test. **d** Representative TTC stained coronal brain slices of three Ctrl IgG and α-OPN antibody-treated mice demonstrating damaged brain tissue area (white) and hemorrhagic transformation of stroke lesions. Expansion of ischemic hemisphere is indicated by a white dotted line. **e** Hemorrhagic transformation frequency of stroke lesions. **f** Edema volume. **g** Stroke volume; *n* = 12 (6 for both males and females) and *n* = 10 (6 males and 4 females) for Ctrl IgG and α-OPN antibody treatment group, respectively; **P < 0.01 and ***P < 0.001, and ns *P* > 0.05 by one-tailed Chi-square test for (**e**) and by two-tailed, unpaired *t*-test, with Welch’s correction when variances were significantly different based on *F*-test, for (**f**) and (**g**). **h** Representative IHC staining demonstrating OPN expression 24 h after tMCAO in the infarct core, peri-infarct region and contralateral hemisphere of animals treated with either control IgG or α-OPN antibody. White and black arrowheads identify vessels and OPN expressing parenchymal cells, respectively. **i** Quantification of OPN expression intensity (arbitrary unit, a.u.) in Ctrl and α-OPN antibody-treated mice 24 h post-ischemic stroke utilizing three images/region/animal, *n* = 12 and *n* = 10 for Ctrl IgG and α-OPN antibody treatment group, respectively; *P < 0.05, §§/††*P* < 0.01, ****/§§§§/††††*P* < 0.001 and ns *P* > 0.05. *Two-tailed, unpaired *t* test comparing the two treatments groups for the same region, ^§^two-tailed, paired *t* test comparison of peri-infarct to infarct core or contralateral hemisphere within the same treatment group/animal, and ^†^two-tailed paired *t* test comparison of the infarct core and contralateral hemisphere within the same treatment group/animal. Scale bars: 5 mm (**d**) 20 μm (**h**)

We focused on targeting OPN with the rationale of robustly improving NVU and BBB function in stroke as multiple NVU cell types have dysregulated osteopontin signaling both in murine and human stroke. The drastically increased expression of OPN in the NVU cells of stroke patients, particularly during the acute phase of ischemic stroke, prompted us to target OPN in mice with acute ischemic stroke. Therefore, adult WT mice (female and male) were subcutaneously injected 4 h post-stroke either with a polyclonal anti-OPN antibody, that has been previously shown to attenuate renal injury after ischemia reperfusion [13], or a control IgG antibody (Fig. 5a). Treatment was followed by assessment of outcome and anti-OPN antibody neutralizing effects in ischemic stroke lesions 24 h post-stroke. Compared to control mice, anti-OPN antibody-treated animals showed a significantly improved outcome for both overall survival (Fig. 5b) and neurological functions, including motor functions and reflexes (Fig. 5c). In addition, based on TTC staining, anti-OPN antibody-treated animals showed a reduced risk for haemorrhagic transformation as well as significantly decreased edema volume, but without changes in stroke volume (Fig. 5d–g). Importantly, improved outcome in mice treated with the anti-OPN antibody, was associated with significantly reduced overall OPN expression both in the peri-infarct region (a region where cells are functionally altered but still vital) and infarct core (a region where cells are mostly dead and necrotic) compared to control animals (Fig. 5h). As observed in normal appearing tissue of human stroke specimen, OPN expression in the contralateral hemisphere in mice was almost absent and did not differ between the control and the treatment group (Fig. 5i). Moreover, for both female and male mice treated with anti-OPN antibody, we observed a significant reduction of OPN expression in the peri-infarct region that was associated with a reduced risk for haemorrhagic transformation and significant decrease in edema volume compared to IgG control animals (Supplementary Fig. 8, online resource). In addition, in trend, the 24 h survival proportions and reflexes in females and males were improved with anti-OPN antibody, thus suggesting a detrimental role of OPN and beneficial effects of OPN neutralization overall for both females and males in acute ischemic stroke.

**Fig. 6:**
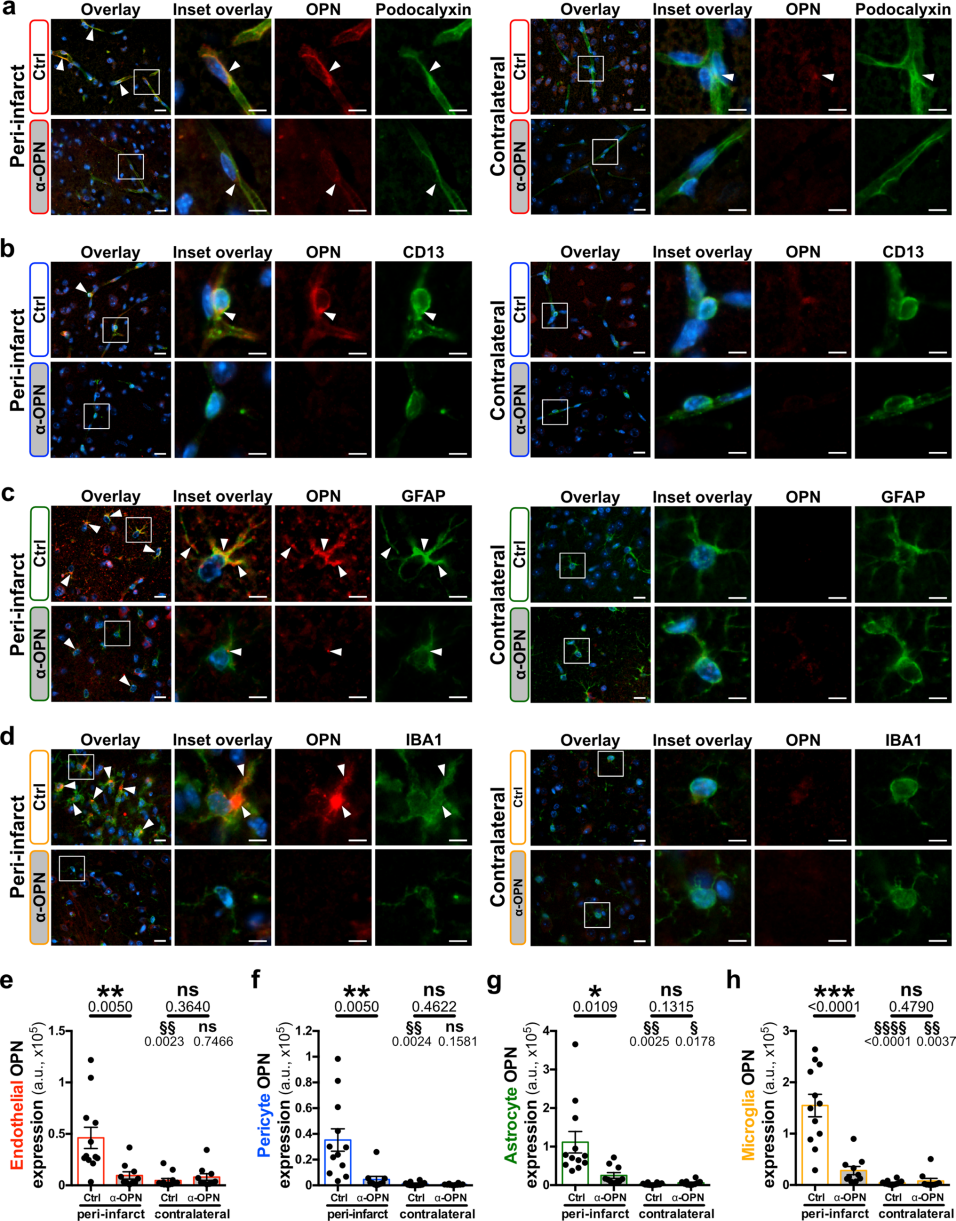
Anti-OPN antibody treatment neutralizes OPN in peri-infarct NVU cells in mice post-acute ischemic stroke. **a**–**d** Representative images of immunofluorescence staining for OPN (red) and cell-specific markers (green) including podocalyxin for endothelial cells (**a**), CD13 for pericytes (**b**), GFAP for astrocytes (**c**) and IBA1 for microglia/macrophages (**d**) in the peri-infarct region (left) and contralateral hemisphere (right) of Ctrl IgG and α-OPN antibody-treated mice 24 h post-stroke. White arrowheads indicate OPN expressing NVU cells. **e**–**h** Quantification of OPN expression in peri-infarct and contralateral endothelial cells (**e**), pericytes (**f**), astrocytes (**g**) and microglia/macrophages (**h**) utilizing 3 images/region/animal, *n* = 12 and 10 for Ctrl and α-OPN, respectively. */§*P* < 0.05, **/§§*P* < 0.01, ***P < 0.001, §§§§*P* < 0.0001 and ns *P* > 0.05. *Two-tailed, unpaired *t* test, with Welch’s correction when variances were significantly different based on *F* test, comparing the two treatments groups for the same region, and ^§^two-tailed, paired t-test comparison of the peri-infarct and contralateral hemisphere within the same treatment group/animal. Scale bars: 10 µm and 5 µm in insets (**a**–**d**)

Reduction of overall OPN expression by the anti-OPN antibody therapy in ischemic stroke lesions was further confirmed for endothelial cells, pericytes, astrocytes and microglia/macrophages independently by immunofluorescence (Fig. 6a–d). Interestingly, compared to controls, in anti-OPN antibody-treated mice a significant decrease in OPN expression was detected in all the individual NVU cell types in the peri-infarct region (Fig. 6e–h), whereas except for microglia/macrophages, no such difference was found in the infarct core (Supplementary Fig. 9a–h, online resource). Comparable results were observed for OPN positive endothelial cells, pericytes, astrocytes and microglia/macrophages ratios, which were significantly decreased in the peri-infarct region of anti-OPN antibody treated animals (Supplementary Fig. 9i–l, online resource). In the infarct core, OPN positive endothelial cells, pericytes, astrocytes ratios did not differ between the treatment and the control group except for microglia/macrophages. For these cells, the significant decrease of OPN expression and OPN positive ratio in the core might be explained by the extensive infiltration of activated microglia/macrophages into the ischemic lesion 24 h post-stroke [48] and, therefore, its efficient targeting by the OPN neutralizing antibody. OPN expression in NVU cells in the contralateral hemisphere was low and did not differ among groups. Reduction of OPN expression in peri-infarct endothelial cells and pericytes of anti-OPN antibody-treated animals was not associated with a decrease of Podocalyxin (Supplementary Fig. 9m, online resource) and CD13 (Supplementary Fig. 9n, online resource), although it has been reported to be involved in proinflammatory processes for the latter [79]. However, the anti-OPN antibody treatment led to a significant decrease of expression levels of the glial activation markers GFAP [33] in peri-infarct astrocytes (Supplementary Fig. 9o, online resource) and IBA1 [53, 104] in microglia/macrophages (Supplementary Fig. 9p, online resource). Interestingly, OPN expression in the astrocyte endfeet is reduced by the treatment like in the rest of the cell body simultaneously to an increase of the marker α-dystroglycan (Supplementary Fig. 11, online resource). Vessel-associated microglia/macrophages also show a reduced OPN expression upon treatment and decreased reactivity illustrated by diminished IBA1 expression in peri-infarct region (Supplementary Fig. 12, online resource). There was no difference between the treatments in the ischemic core in the cell types markers with the exception of podocalyxin in endothelial cells and IBA1 in vessel-associated microglia/macrophages that suggests a potential restoration of vasculature in the core with the anti-OPN therapy (Supplementary Figs. 9 and 12, online resource). As for neurological scoring, male and female mice treated with anti-OPN antibody presented similar response to the treatment with regards to OPN expression (Supplementary Fig. 10, online resource). This was also confirmed qualitatively by microglial TMEM119 staining for both human and mouse specimen (Supplementary Fig. 13, online resource). In addition, we detected only rare lymphocyte infiltration in core, peri-infarct or contralateral regions 24 h post-ischemia in both control and anti-OPN treated animals (Supplementary Figs. 14, 15 and 16, online resource).

Importantly, the specificity of the therapeutic anti-OPN antibody is indicated by its co-localization with endogenous OPN in all 3 regions post-ischemic stroke and in healthy animals (Supplementary Fig. 17a–c, online resource). The antibody’s ability to cross the BBB is also indicated by its co-localization with endogenous OPN in parenchymal cells. In addition, in presence of the blocking therapeutic antibody, there was no change in OPN staining in untreated mice post-stroke (Supplementary Fig. 17d, online resource). Based on these results, we suggest a transcriptional/translational regulation of OPN in the presence of the therapeutic antibody. Taken together, these results suggest that the degree of OPN expression in stroke lesions, particularly in the peri-infarct region, correlates with the severity of disease and indicates that neutralization of OPN leads to improved outcome post-stroke.

### Improved stroke outcome upon therapeutic targeting of NVU cells by anti-OPN antibody is associated with enhanced BBB function

**Fig. 7:**
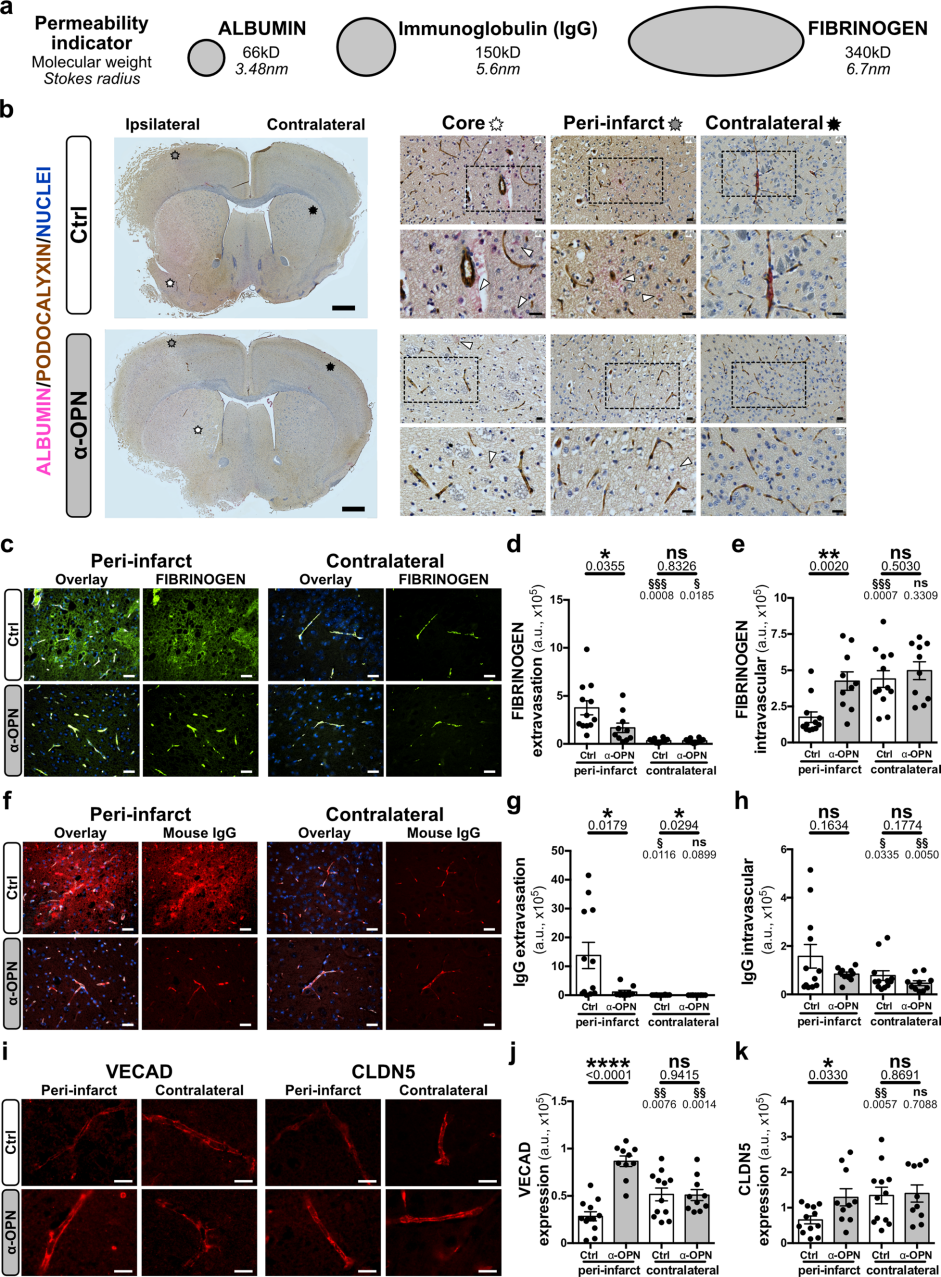
Anti-OPN antibody treatment improves BBB function and NVU cell response in acute ischemic stroke. **a** Molecular weight and Stokes radius of endogenous markers of permeability. **b** Representative images of immunohistochemical co-staining for permeability marker albumin (pink) and vessel marker Podocalyxin (brown) in the core, peri-infarct and contralateral regions of Ctrl IgG and α-OPN antibody-treated mice. Arrows point to regions of increased albumin extravasation that are prominent in vehicle treated group compared to α-OPN antibody-treated mice in the core and peri-infarct regions. In the contralateral hemisphere albumin staining is restricted to the vessels. **c**–**h** Representative images of immunofluorescence staining and quantification for fibrinogen (green, **c**–**e**) and mouse immunoglobulin (IgG, red, **f**–**h**) extravasation in the peri-infarct region and contralateral hemisphere of Ctrl IgG and α-OPN antibody-treated mice. **i**–**k** Representative images of endothelial adherens and tight junctions VECAD and CLDN5 (**i**) in the peri-infarct and contralateral hemisphere of Ctrl IgG and α-OPN antibody-treated mice with the corresponding quantification (**j**,** k**). Quantifications were done utilizing three images/region/animal, *n* = 12 and *n* = 10 for Ctrl and α-OPN, respectively; */§*P* < 0.05, **/§§*P* < 0.01, §§§*P* < 0.001, ****P < 0.0001 and not significant (ns) *P* > 0.05. *Two-tailed, unpaired *t* test, with Welch’s correction when variances were significantly different based on *F* test, comparing the two treatment groups for the same region, and ^§^Two-tailed, paired *t* test comparison of the peri-infarct and equivalent contralateral region within the same treatment group/animal. Scale bars: 20 µm for all except for (**g**): 10 µm and 5 µm in inset. Podocalyxin (white) was used as vessel marker as shown in overlay pictures (**a**, **d**, **j**, **m**)

Based on the significantly decreased edema volume and reduced risk for haemorrhagic transformation upon anti-OPN antibody treatment after ischemic stroke in mice, we evaluated the effects of the OPN neutralizing therapy on NVU cells and in particular on BBB function. Compared to controls, reduction of OPN expression in endothelial cells in the peri-infarct region (Fig. 6a, e) of anti-OPN antibody-treated animals was associated with reduced vascular permeability to albumin (Fig. 7a, b, Supplementary Fig. 18, online resource), a marker for protein leakage assessed commonly by Evans blue dye permeability due to its albumin binding ability [45]. Decreased permeability was also observed with both fibrinogen and IgG, as indicated by their significantly reduced extravascular and increased intravascular signals (Fig. 7c–h). Furthermore, increased expression levels of the adherens and tight junction proteins VECAD and CLDN5 (Fig. 7i–k) were observed, suggesting that detrimental effects of OPN on endothelial cells and BBB function are attenuated by the therapeutic antibody. With exception of mouse IgG extravasation, the protective effect of OPN neutralization on BBB was not observed in the infarct core, where cells are severally damaged and, therefore, most probably do not benefit from the therapy anymore (Supplementary Fig. 19, online resource). It is of note that disassembly of the BBB junctional proteins observed in acute ischemic stroke in mice is not due to vessel loss as podocalyxin expression remained unchanged between treatment groups and regions (Supplementary Fig. 9m, online resource). Furthermore, the permeability and BBB expression analysis were similar between males and females (Supplementary Fig. 20, online resource).

**Fig. 8:**
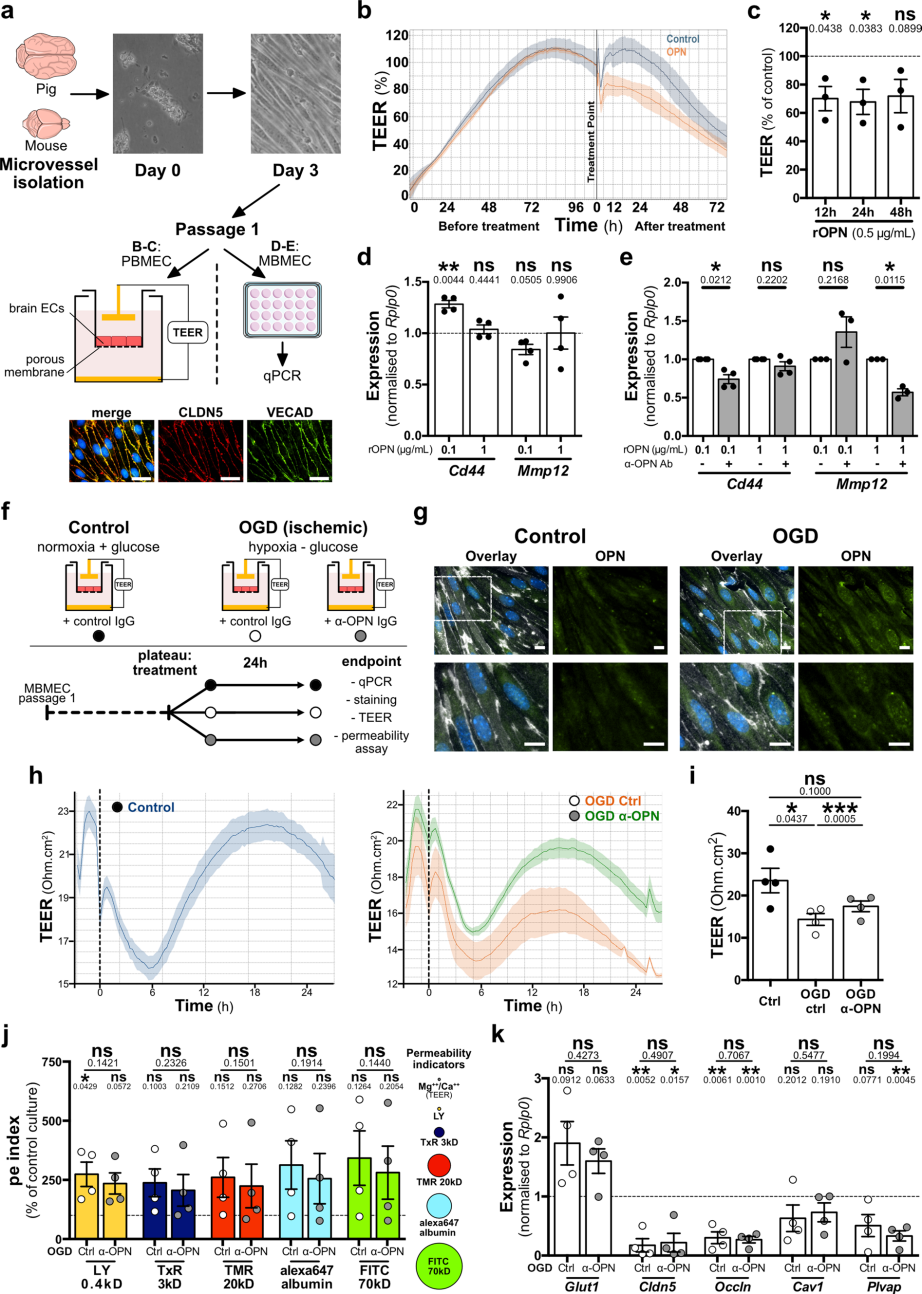
Impairment of BBB by OPN and its response to anti-OPN therapy in vitro. **a** Schematic depicting the isolation and culture of primary porcine or mouse brain microvascular endothelial cells (PBMEC or MBMEC) followed by transendothelial electrical resistance (TEER) measurement across the endothelial monolayer or qRT-PCR experiment. Immunostaining of transwell inserts representing well-developed tight and adherens junctions of the PBMEC monolayer including CLDN5 (red) and VECAD (green). **b** Representative graph for continuous TEER values of the PBMEC monolayer treated with recombinant murine OPN (0.5 µg/mL) or vehicle (Control). **c** 12, 24 and 48 h TEER values of OPN-treated PBMEC normalised to their respective time-point control; *n* = 3 independent experiments, **d** Activation of OPN signaling was assessed by qRT-PCR on MBMEC treated 24 h with recombinant murine OPN and normalised to their untreated control. *n* = 4 independent experiments. **e** Neutralisation effect of the anti-OPN antibody (3 µg/mL) was assessed by qRT-PCR on MBMECs treated with recombinant murine OPN for 24 h *n* = 3–4 independent experiments. **f** Schematic depicting the experimental paradigm for oxygen-glucose deprivation (OGD, 1% O_2_, glucose-free basal medium) performed on MBMEC followed by qRT-PCR, staining of the cells, TEER measurement or permeability assay across the endothelial monolayer. Control condition cells were cultured in 19.5% O_2_ and 5.6 mM glucose containing basal medium. **g** Representative images of osteopontin (OPN, green) in endothelial monolayer 24 h post-OGD treatment. CD31 (white) was used as endothelial marker and DAPI (blue) to reveal nuclei. **h** Representative graph for continuous TEER values of the MBMEC monolayer in control normoxic conditions (treated with isotype control) and for OGD conditions—isotype control or anti-OPN antibody treated. **i** Quantification of 24 h TEER values of MBMEC treated with isotype control or anti-OPN antibody in control or OGD conditions; *n* = 4 independent experiment. **j** Permeability (pe) index values of fluorescent tracers of different molecular weight through MBMEC monolayer subjected to OGD and treated with isotype control or anti-OPN antibody. Results were normalized to pe index values obtained with inserts from each respective control condition (dashed line); *n* = 4 independent experiments. **k** BBB disruption by 24 h OGD and effect of the anti-OPN antibody treatment were assessed by qRT–PCR on MBMECs. Results were normalized to each respective control culture condition (dashed line); *n* = 4 independent experiments, *P < 0.05; **P < 0.01, ***P < 0.001 and ns *P* > 0.05 by two-tailed, paired *t* test (**c**–**e**, **i**–**k**). Scale bars: 10 µm (**a**, **g**)

To more directly investigate the effect of OPN on BBB permeability, we isolated and cultured primary porcine or mouse brain microvascular endothelial cells (PBMEC or MBMEC) for subsequent transendothelial electrical resistance (TEER) or qRT–PCR experiments as these cells maintain decent BBB characteristics in vitro with well-developed BBB junctional proteins including CLDN5 and VECAD [91] (Fig. 8a). The TEER of the PBMEC monolayer was continuously recorded and cells were treated at the plateau phase indicating the formation of mature BBB in vitro (Fig. 8b). Compared to control, recombinant OPN (0.5 µg/mL) treated PBMEC exhibited lower TEER values at 12 and 24 h post-treatment that returned to normal levels by 72 h, indicative of an acute and transient barrier opening effect of OPN (Fig. 8b, c). The upregulation of *Cd44*, a crucial OPN pathway member, upon treatment with recombinant OPN in MBMEC, prove that OPN signalling is indeed affected in brain endothelial cells (Fig. 8d). In addition, both *Cd44* and *Mmp12*, another target of OPN signalling, were downregulated in the presence of the neutralisation antibody used in vivo (Fig. 8e). These effects were OPN dose dependent with *Cd44* regulatio*n* prominent at lower physiological doses, whereas *Mmp12* at higher doses, potentially reflecting the expression level of these genes with *Cd44* being highly expressed in brain endothelial cells compared to low levels of *Mmp12 *(50-fold less than *Cd44*).

To reproduce the ischemic conditions observed in vivo with the MCAO model, we used the in vitro oxygen–glucose deprivation (OGD) model (Fig. 8f) [31, 109]. Compared to control condition, OPN protein expression is increased in endothelial monolayer 24 h post-OGD treatment (Fig. 8g, Supplementary Fig. 21a, online resource) and, therefore, reproduce the in vivo observations (Figs. 5 and 6). At the functional level, OGD weakens the barrier properties of the endothelial monolayer as TEER is reduced by about 60% compared to control culture (Fig. 8h–i, Supplementary Fig. 21c, d, online resource). Interestingly, OPN neutralisation by the antibody partially restores the BBB disruption to about 75% of the control culture. The increased permeability to ions (Ca^++^/Mg^++^) reflected in the reduced TEER values during OGD was also confirmed with permeability assay using fluorescent tracers of different sizes (Fig. 8j, Supplementary Fig. 21b, e, online resource). Even though only significant for Lucifer Yellow, permeability index of all tracers in trend increased in OGD cultures. Interestingly, OPN neutralisation only improved the permeability to lower size tracers—ion permeability (reflected in the TEER values) and in trend for lucifer yellow, suggesting a size dependent BBB tightening with anti-OPN IgG in vitro (Fig. 8j, Supplementary Fig. 21e, online resource). At the molecular level, the BBB dysregulation observed in OGD could be explained by reduced expression of the junction markers *Cldn5* and *Occln* (Fig. 8k, white dots). Increased *Glut1* expression confirmed induction of OGD, whereas vesicle markers *Cav1* and *Plvap* were reduced at the RNA level upon OGD, potentially a compensatory regulation to the increased protein levels as observed previously in vivo in stroke. [68] While there were some trends, the OPN neutralisation did not, however, restore RNA expression levels back to the control normoxic culture conditions (Fig. 8k, grey dots).

**Fig. 9:**
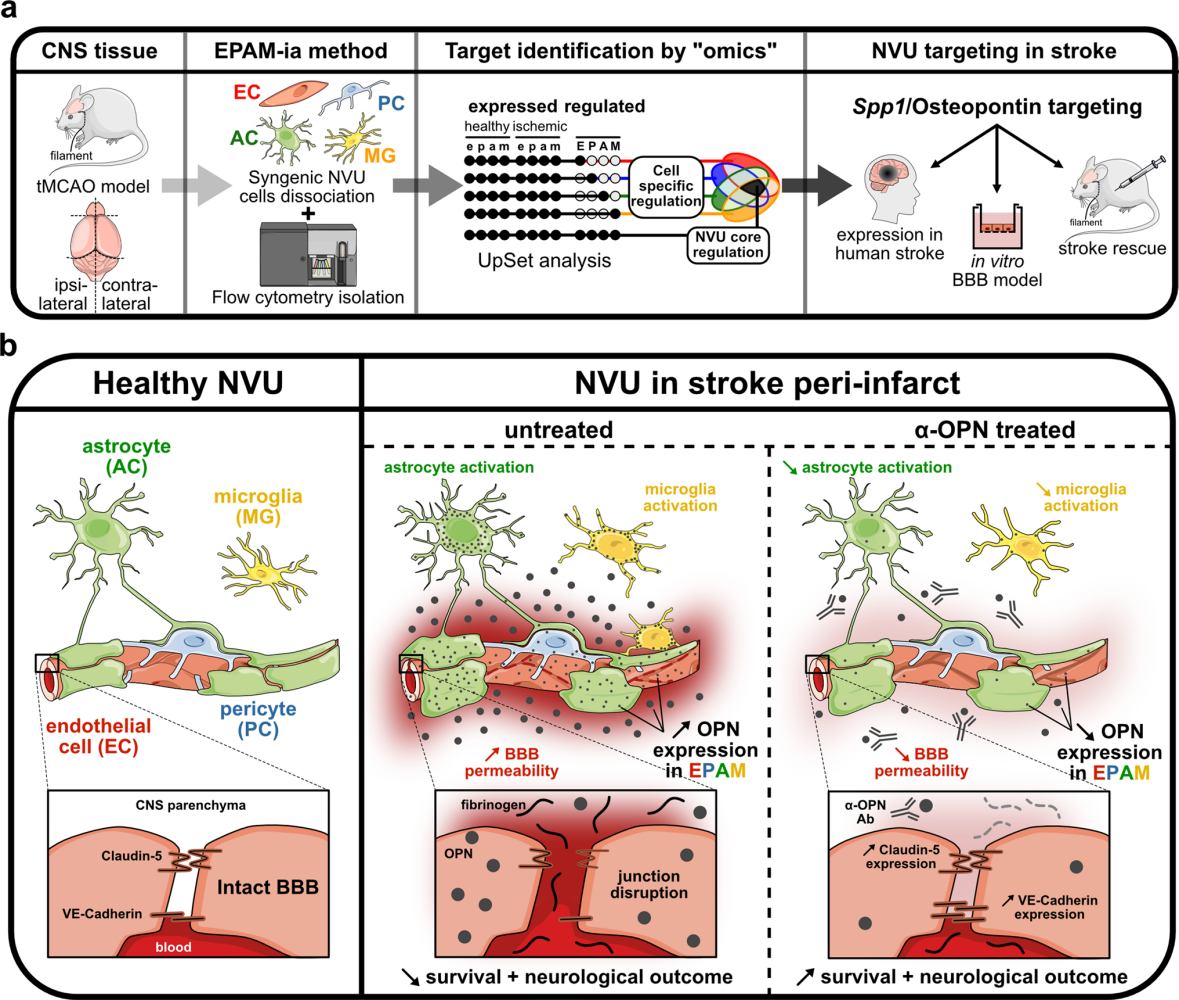
Overview of EPAM-ia method and OPN targeting as potential therapeutic approach in acute ischemic stroke. **a** Overview of the strategy applied for identification of novel candidates in multiple NVU cell types and their co-targeting in stroke. **b** Summary schematic showing dysregulation of OPN at the NVU and BBB impairment in ischemic stroke and their response to anti-OPN therapy

Overall, our approach in identification of therapeutic candidates at the NVU regulating BBB function and their co-targeting in multiple NVU cell types was successful in ischemic stroke (Fig. 9a). Using which, we identified osteopontin as a therapeutic target at the NVU in ischemic stroke and could demonstrate improvement of NVU cell function and BBB recovery upon OPN targeting, that was associated with improved survival and neurological outcome post-ischemic stroke (Fig. 9b).

## Discussion

There is emerging evidence that dysregulation of NVU cells is critically involved in the pathophysiology of acute ischemic stroke, particularly by promoting early impairment of the BBB [112, 127]. An essential feature of NVU dysfunction and BBB breakdown is the degradation of BBB junctional proteins that can result in vasogenic brain edema and haemorrhagic transformation [5, 67, 100, 103, 125], both of which are major complications of ischemic stroke. A therapeutic approach targeting multiple NVU cell types simultaneously and restoring the corresponding NVU cell functions to preserve BBB integrity and further reduce the risk of acute ischemic stroke complications is not available yet. In this regard, there is need for a method enabling the identification and validation of specific therapeutic targets in all the dysregulated NVU cells in ischemic stroke that could pave the way for the development of new therapeutic strategies aiming at stabilizing the BBB. The present study adds to this body of work by demonstrating an advanced method for simultaneous isolation of multiple NVU cell types from ischemic brain tissue that allows for in-depth analysis of the NVU transcriptome (Fig. 9a). Bioinformatic dissection of the NVU transcriptome identified OPN to be a potential therapeutic target that was dysregulated in multiple NVU cell types in acute ischemic stroke. Neutralization of this protein using an anti-OPN antibody improved the function of NVU cells overall as demonstrated by decreased glial activation and preservation of BBB integrity along with improved survival and neurological outcome (Fig. 9b). The potential clinical significance of these findings is that the anti-OPN antibody therapy might augment the current approved reperfusion therapies in acute ischemic stroke treatment by minimizing deleterious effects of ischemia-induced BBB disruption.

### EPAM-ia method: an advanced approach for the investigation of NVU and BBB dysfunction in ischemic stroke

Comprehensive understanding of the function of the NVU is important for all aspects of normal CNS vascular physiology and pathogenesis of many CNS diseases, including ischemic stroke. It is well appreciated that several NVU cell types regulate BBB function [61]. Therefore, it is crucial to isolate all of them simultaneously to investigate their crosstalk in pathology. Furthermore, as the NVU components are anatomically close, their syngenic isolation is critical to obtain pure populations of each cell type. Hence, we developed the EPAM-ia method for syngenic isolation and analysis of the major NVU cell types, with the objective to identify robust therapeutic targets in dysregulated NVU cells in ischemic stroke and other CNS diseases associated with BBB dysfunction.

WT and transgenic animals have been already used successfully in the field to isolate endothelial cells, pericytes, astrocytes and microglia [3, 6, 19, 23, 36, 46, 111]. However, the number of NVU cell types isolated simultaneously was limited to one or two (Supplementary Table 5, online resource). Furthermore, the application of transgenic mice to pathology models is challenging. More recently, multiple neural cell types were simultaneously isolated by FACS, without subjecting them to higher temperatures during enzymatic steps [102]. However, this protocol includes fixation followed by flow cytometry for intracellular antigens, which may lead to changes in gene expression [94]. In such cases, in particular for endothelial cells and pericytes, it might be more informative to perform the expression analysis on vessel fragments that can be quickly isolated without the use of enzymes [10, 27, 41, 71]. Many of these aspects are addressed with the EPAM-ia method presented here, allowing for the simultaneous isolation of endothelial cells, pericytes, astrocytes and microglia (Supplementary Fig. 1a, b, online resource; Fig. 1a, b). The novelty of the EPAM-ia method is the application of a multi-step cell-specific dissociation procedure followed by utilization of established cell-specific markers for FACS to isolate all the major NVU cell types from the same starting healthy and ischemic mouse brain tissue. The viability, yield, and purity of sorted cells were high with the method (Supplementary Fig. 1, online resource and Fig. 1), making it suitable for several applications, such as RNA-Sequencing, proteomics, and cell culture. This method is also suitable for NVU cell isolation from one mouse (data not shown) making it extremely valuable for transgenic animals and for single-cell sequencing approaches downstream.

One of the major criteria of the EPAM-ia method was to apply enzymes and mechanical dissociation specific for each cell type. A key step in the method was to filter the cells after the mild papain digestion to separate the vascular cells from glial cells. We further designed our FACS strategy to include specific markers for each cell type and simultaneously exclude other NVU cell types (Supplementary Figs. 1b and 2a, online resource; Fig. 1b). With this, we obtained pure NVU populations from healthy and ischemic brain tissue as indicated by qRT–PCR analyses on the whole sorted sample (Supplementary Fig. 1e–h, online resource; Fig. 1f, g), which is unbiased and more reliable compared to immunostaining of small subsets of sorted cells [19].

Application of the EPAM-ia method to a murine stroke model followed by bulk RNA-Sequencing and bioinformatic analyses resulted in a new transcriptome database for the investigation of NVU dysfunction and BBB impairment in acute ischemic stroke. Moreover, the transcriptome database presented here is the first one for brain mural cells in ischemic stroke and for multiple NVU cell types from the same source in any CNS disease. Bioinformatic analyses of the sequencing data revealed significant regulation of several genes and pathways in each of the cell types collected, including several that have not been reported yet, and several that have been described to play a potential role in stroke pathophysiology (Fig. 2) [8, 15, 34, 41, 47, 57, 60, 65, 66, 89, 101, 107, 117, 124, 126]. For the latter, however, the cellular source has been described for only a few genes in ischemic stroke [8, 41, 60, 65, 66, 89, 101, 107, 124]. Here, we report to the best of our knowledge for the first time the cellular source of significantly regulated genes and pathways in acute ischemic stroke, including *Cxcr2* in endothelial cells, *Ccl5*, *Il11*, *Il6*, *Lcn2*, and TNF signalling pathway and cytokine–cytokine receptor interaction in pericytes, cytokine–cytokine receptor interaction in astrocytes, and *Ilrn* and *Mmp12* in microglia (Fig. 2e–h). Furthermore, using the EPAM-ia method we can identify genes regulated in a cell specific manner and that are commonly regulated in multiple cell types, which cannot be obtained with single cell type isolation and analyses described previously.

It was recently reported that CNS endothelial cells from multiple neurological diseases, including stroke, share a common gene signature [89], suggesting a common BBB dysfunction module. However, as the BBB is co-regulated by several NVU cell types and not just cell autonomously by endothelial cells, targeting all the NVU cells in concert might lead to more effective therapies in ischemic stroke affecting the BBB function. Therefore, using our method, we investigated genes commonly regulated in the major NVU cell types in ischemic stroke to identify robust therapeutic targets in dysregulated NVU cells affecting BBB function. For visualization of multiple intersections, we applied the UpSet analysis [72] to our binary transformed RNA-Sequencing data (Fig. 3a, b). We obtained several novel target genes in stroke using the EPAM-ia method in combination with the downstream RNA-sequencing and the UpSet analysis, which could not be obtained from a single cell type analysis. Using the NVU transcriptome database, we obtained genes that were up-/downregulated, induced or shut down in only a single cell type or in a combination of vascular and glial cells, making them specific targets due to their expression pattern (Fig. 3a). Furthermore, we could identify few genes from the UpSet analysis that were regulated in opposite directions in NVU cell types suggesting a crosstalk of these cell types in the specific signaling pathway. Even for genes regulated in individual NVU cell types (as in Fig. 3a, b), a database comprising data of all isolated NVU cell types from the same material is crucial for confirming the cell type-specific regulation and targeting of the candidate genes.

We have utilized established markers to isolate all populations of astrocytes and microglia. However, we speculate that when vessel-associated astrocytes or microglia specific cell-surface markers are established [123], they can be incorporated in to our FACS strategy to isolate them and thus obtain more specific gene regulation in these cell types corresponding to their function at the NVU.

While several promising therapeutic targets in stroke were obtained from our NVU transcriptome database, in the current study we focused on genes commonly regulated in all the NVU cell types (EPAM in Fig. 3b). Our analysis revealed 14 genes differentially expressed in all the four NVU cell types 24 h post-ischemic stroke. Analysis of these 14 genes revealed *Spp1* and its signaling pathway members, namely, *Cd44*, *Timp1* and *Mmp12* upregulated in all the NVU cells, suggesting *Spp1* as a therapeutic target in stroke (Fig. 3c–g).

### Deleterious effects of OPN on BBB in acute ischemic stroke are ameliorated by anti-OPN antibody therapy

*Spp1* encodes for the highly phosphorylated glycoprotein osteopontin (OPN), which functions as an adhesive extracellular matrix protein and proinflammatory cytokine [80]. OPN is critically involved in proinflammatory response and extracellular matrix remodelling by binding to its receptor CD44 [116]. In the CNS, OPN expression is weak under physiological conditions, whereas it is significantly upregulated under pathological conditions [77]. OPN appears to act as a double-edged sword triggering detrimental effects in some CNS diseases [11, 14, 49] and functioning as a neuroprotectant in others [52, 119], as reviewed by Zhou et al. [130]. In ischemic stroke, the effect of OPN is still unclear. It was previously shown that OPN reduces infarct size in a murine model of MCAO, where recombinant OPN was administered intraventricularly before and immediately after the occlusion [85]. Along similar lines, benefit of OPN was shown in neo-natal rats post-hypoxia–ischemia, using recombinant OPN therapy [28]. These studies show beneficial effects of OPN in stroke; however, it is unclear which cell types are affected by the recombinant OPN therapy. Thus, the mechanism of recombinant OPN action is unclear from these initial studies. In addition, in another study macrophage induced OPN was shown to contribute to BBB repair and improve ischemic recovery in a murine model of photothrombic stroke [38]. In this model the stroke lesion is a small defined region and the mechanism of OPN appears to be via activation of astrocytes by OPN leading to reduced scar size, thus affecting stroke recovery in the long term and not affecting BBB function in the acute stages. The beneficial effects of OPN in long-term stroke were also reported in a rat neo-natal ischemia model [58]. Importantly our study among others already demonstrates a dramatic upregulation of OPN, in early acute stages of stroke and reduced levels in the long-term recovery from stroke patients. Furthermore, our study shows beneficial effects of OPN neutralization in acute ischemic stroke with an anti-OPN antibody in a pre-clinical murine model.

Previous transcriptomic studies utilizing infarct and peri-infarct tissues reported the upregulation of *Spp1*.[3, 18] However, the cellular source of *Spp1* and its signaling pathway members (*Cd44*, *Timp1* and *Mmp12*) at the NVU in acute ischemic stroke have not been investigated yet. In the current study, these 4 genes were significantly upregulated in all the major cell types of the NVU in acute ischemic stroke based on our RNA-Sequencing data set (Fig. 3c–g). This has important implications as these genes and their encoded proteins have been reported to be critically involved in ischemic stroke pathophysiology. TIMP1 unfolds protective neurovascular effects in ischemic stroke [37], whereas both MMP12 and osteopontin receptor CD44 may critically contribute to ischemic brain damage [15, 114], suggesting that these proteins/genes may all be important therapeutic targets in ischemic stroke. However, as these targets may be critically regulated by *Spp1*, which shows the strongest upregulation in all the major NVU cell types in our data set, we focused on evaluating the role of OPN in NVU and BBB function and its value as a therapeutic target in acute ischemic stroke treatment.

We first confirmed upregulation of OPN in human brain tissue sections of all three stages of ischemic stroke compared to normal appearing tissue. We observed the highest expression in stage I, which represents the acute phase of ischemic stroke, that was further reduced in stage II, with the lowest OPN expression being in stage III (Fig. 4). This temporal pattern of OPN induction in the ischemic brain was observed in all the NVU cells and in some parenchymal cells. These effects parallel previously reported serum OPN levels in patients suffering ischemic stroke. In that study, peak values of serum OPN observed in acute phase were directly associated with increased lesion size and worse post-stroke neurological scores, suggesting a detrimental role of OPN [12]. The higher blood OPN levels in the acute phase of ischemic stroke might be explained by ischemia-induced BBB disruption, promoting cerebral OPN to leak into circulation. On the other hand, increased blood OPN levels reported in the above study [12] might be a result of BBB breakdown induced by upregulated cerebral OPN during the acute phase of ischemic stroke as detected in stroke patients of our study. Along this line, OPN was found to be highly expressed in BBB-damaged vessels of stroke prone hypertensive rats suggesting a crucial role for OPN in BBB dysfunction [54]. OPN-induced BBB damage is also supported by our in vitro permeability data using primary porcine brain endothelial cells that indicated significant BBB disruptive effects of recombinant OPN that was prominent merely 24 h post-treatment (Fig. 8b, c). This was also recapitulated in an in vitro model of stroke by oxygen–glucose deprivation (OGD), where we observed increased level of OPN in brain endothelial cells (Fig. 8g). A potential explanation for this effect might be the BBB permeability effects of OPN based on its induction of VEGF, a vascular permeability factor [21], which is known to induce BBB impairment and vessel leakage [70].

The acute BBB impairing effects of OPN suggested by our NVU database and confirmed in our in vitro studies and stage I stroke patients were underpinned in an in vivo model of acute ischemic stroke. Therapeutic neutralization of OPN in the clinically significant therapeutic window (4 h post-occlusion in the tMCAO model) using an anti-OPN antibody in mice led to significant BBB improvement in the peri-infarct region (Fig. 5a). This was reflected by higher expression of adherens and tight junction molecules in capillary endothelial cells, and reduced vascular leakage of albumin, fibrinogen and endogenous IgG (Fig. 7a–h). Importantly, these preserving effects of the anti-OPN antibody therapy on BBB also resulted in decreased edema volume and reduced risk for haemorrhagic transformation (Fig. 5d–f). These results support our conclusion that OPN may have detrimental effects on BBB function in the early phase of ischemic stroke that are minimized by the anti-OPN antibody therapy primarily in the peri-infarct region. Despite the strong BBB preserving effects of the anti-OPN treatment in the peri-infarct region of treated animals, we did not detect a decrease in ischemic lesion volume (Fig. 5d–g). This is in contrast to other studies, which either reported neuroprotective or neurodetrimental effects of OPN and/or its receptor [85, 114]. It is known from previous studies that OPN could have cell survival effects in neurons both in vitro and in vivo.[17, 85, 131] On the other hand, osteopontin receptor CD44 deficiency has been shown to have beneficial effects in cerebral ischemia [114] and in glioma [92]. These opposing effects can be explained by different downstream signalling pathways of OPN with OPN binding to integrin receptors leading to cell survival (via PI3 and MAPK), whereas via CD44 leading to MMP and VEGF mediated BBB breakdown [131]. While the cell survival pathways are activated in neurons, pathways leading to BBB breakdown are activated in the NVU cells as indicated by upregulation of CD44 in all the NVU cells in the current study from the transcriptomics analysis (Fig. 3). In this regard, we speculate that OPN is detrimental in the initial stages of ischemic stroke when BBB breakdown is prominent, whereas in the late stages of stroke recovery, OPN is beneficial for neuronal cell survival and recovery. Along this line, in the current study of anti-OPN treatment in the early stages, we only observed an improvement of BBB function including edema reduction, whereas no beneficial effects were observed in infarct volumes, that is potentially dependent on the dosage of anti-OPN IgG (Figs. 5, 7). We also speculate that the anti-OPN IgG action is rather by neutralizing the OPN in the extracellular space and parenchymal cells under leaky BBB conditions along with its action also at the NVU cells. In this regard, our in vitro data of BBB disruption with recombinant OPN and in OGD conditions with increased OPN levels supports our in vivo stroke data. Based on the rescue experiments with anti-OPN antibody (Fig. 8e, i–k) we suggest that the OPN reducing action in the NVU cells contribute to the reduced BBB permeability. This potentially occurs by suppressing the OPN/CD44 signaling in the NVU cells primarily endothelial cells, affecting its downstream signaling mediated by MMP12, and VEGF that influences BBB integrity.

Induction of OPN was also shown to be associated with increased astrocytes and microglia reactivity in an animal model of Multiple Sclerosis, and correlated with the severity of the disease [14]. Glial activation, which can have detrimental effects in ischemic stroke [30, 69, 86], was attenuated in the peri-infarct region by the anti-OPN antibody therapy as indicated by decreased expression of the glial activation markers GFAP [33] and IBA1 [53, 104] in astrocytes and microglia in the peri-infarct region, respectively (Fig. 6c, d; Supplementary Fig. 9o, p, online resource). However, as Iba1 is not a specific marker for microglia, we cannot distinguish the effects of anti-OPN therapy between microglia and macrophages. OPN expression was also decreased in astrocyte endfeet and vessel-associated microglia/macrophages (Supplementary Figs. 11 and 12, online resource) and not just overall by anti-OPN therapy post-stroke (Fig. 6). These data suggest that astrocytes and microglia/macrophages directly associated with the vessels are involved in pathogenesis of stroke via osteopontin as they potentially have a greater impact on BBB function compared to non-vessel-associated counter parts [59].

We thus demonstrate that the anti-OPN antibody treatment results in the rescue of BBB function in ischemic stroke, leading to a decrease in cerebral edema and to a reduced risk for haemorrhagic transformation. The improved BBB function explains the reduced post-stroke neurological severity scores and increased survival in treated animals compared to controls (Fig. 5). The specificity of the anti-OPN antibody in neutralizing OPN was confirmed in vitro in brain endothelial cells at the mRNA level by a reduction of *Cd44* and *Mmp12* transcripts at different doses of recombinant OPN (Fig. 8d, e). Furthermore, co-localization of the therapeutic anti-OPN antibody with endogenous OPN in both parenchymal and NVU cells demonstrated target engagement and BBB permeability of the therapeutic antibody (Supplementary Fig. 17, online resource). These preclinical findings have important implications for acute treatment of ischemic stroke in the clinical setting as both brain edema and haemorrhagic transformation are complications that can be aggravated by recombinant tissue plasminogen activator (rt-PA)—a thrombolytic drug, when administered outside the therapeutic time window [42]. Therefore, administration of the anti-OPN antibody might not only minimize the deleterious effects of ischemia-induced BBB disruption but also expand the therapeutic window of rt-PA, thus allowing rt-PA to be administered to a wider spectrum of ischemic stroke patients. Importantly, improved post-stroke outcome was observed both in males and females (Supplementary Fig. 5, online resource). This is of note as differences in stroke outcome and response to therapy have been described between genders in humans and animal models of stroke [50, 110].

In conclusion, the BBB has never been therapeutically targeted considering multiple cell types of the NVU in ischemic stroke, although it is well accepted that they co-regulate the BBB function [67, 112, 132]. Application of the EPAM-ia method for concomitant isolation and analysis of NVU cells, could thus unveil novel therapies addressing BBB dysfunction in neurological diseases by targeting the whole NVU, as demonstrated by osteopontin targeting in ischemic stroke (Fig. 9a). Overall, our data demonstrate a detrimental role of OPN in BBB function particularly at early acute stages and suggest OPN as a novel therapeutic target in acute ischemic stroke based on BBB improvement and rescue in ischemic stroke with anti-OPN antibody therapy (Fig. 9b).

## Supplementary Information

Below is the link to the electronic supplementary material.

Supplementary file1 (PDF 46465 kb)

## Abbreviations

AC: Astrocyte
BBB: Blood–brain barrier
EC: Endothelial cell
EPAM-ia: Endothelial cells pericytes astrocytes microglia-isolation and analysis
FACS: Fluorescence-activated cell sorting
H&E: Hematoxylin and eosin
IHC: Immunohistochemistry
MBMEC: Mouse brain microvascular endothelial cells
MG: Microglia
NAT: Normal appearing tissue
NVU: Neurovascular unit
OPN: Osteopontin
PBMEC: Porcine brain microvascular endothelial cells
PC: Pericyte
rt-PA: Recombinant tissue plasminogen activator
TEER: Transendothelial electrical resistance
tMCAO: Transient middle cerebral artery occlusion
VA: Vessel-associated
