# Supplementary material for: Profiling the neurovascular unit unveils detrimental effects of osteopontin on the blood–brain barrier in acute ischemic stroke
Source: Acta Neuropathol. 2022 Jun 25;144(2):305–37. doi: 10.1007/s00401-022-02452-1 (PMC9288377; doi:10.1007/s00401-022-02452-1)
Supplement: Supplementary file 1 — Supplementary file1 (PDF 46465 kb) [file 401_2022_2452_MOESM1_ESM.pdf]

Profiling the neurovascular unit unveils detrimental effects of osteopontin on the blood-brain barrier in acute ischemic stroke

Daniel Spitzer,[1,2,10,#] Sylvaine Guérit,[1,#] Tim Puetz,[1,2] Maryam I. Khel,[1] Moritz Armbrust[1], Maika Dunst,[1] Jadranka Macas,[1] Jenny Zinke,[1] Gayatri Devraj,[3] Xiaoxiong Jia,[1] Florian Croll,[1] Kathleen Sommer,[1] Katharina Filipski,[1,6,7] Thomas Freiman,[4,10] Mario Looso,[5] Stefan Guenther,[5] Mariangela Di Tacchio,[1] Karl-Heinz Plate,[1,6,7,8,10] Yvonne Reiss,[1,6,7,10] Stefan Liebner,[1,8,9,10] Patrick N. Harter,[1,6,7,10] and Kavi Devraj[1,7,10*]

[1] Edinger Institute (Institute of Neurology), Goethe University Hospital, Frankfurt, 60528, Germany

[2] Department of Neurology, Goethe University Hospital, Frankfurt, 60528, Germany

[3] Department of Microbiology, Goethe University Hospital, Frankfurt, 60528, Germany

[4] Department of Neurosurgery, Neurology and Neurosurgery Center, Goethe University Hospital, Frankfurt, 60528, Germany

[5] Max Planck Institute for Heart and Lung Research, Bad Nauheim, 61231, Germany

[6] German Cancer Consortium (DKTK) Partner site Frankfurt/Mainz, 60528, Frankfurt, Germany and German Cancer Research Center (DKFZ), Heidelberg, 69120, Germany

[7] Frankfurt Cancer Institute, Goethe University Hospital, Frankfurt, 60528, Germany

[8] German Center for Cardiovascular Research (DZHK), Partner Site Frankfurt/Mainz, Frankfurt, 60528, Germany

[9] Excellence Cluster Cardio Pulmonary System (CPI), Partner Site Frankfurt, Frankfurt, 60528, Germany

[10] Center for Personalized Translational Epilepsy Research (CePTER), Frankfurt, 60528, Germany

# These authors contributed equally to this work

*Corresponding Author: Dr Kavi Devraj, BBB Transport Dynamics, Institute of Neurology (Edinger Institute), Goethe University Hospital, Heinrich-Hoffmann Str. 7, Frankfurt, 60528, Germany

E-mail: kavi.devraj@kgu.de Telephone: +49- 69-6301-84167 Fax: +49-69-6301-84150

Supplementary Table 1. Sequences of the primer pairs used for quantitative real-time PCR

**Gene name**	**Forward (5'-3')**	**Reverse (5'-3')**
Rplp0	gtgtttgacaacggcagcatt	tctccacagacaatgccagga
Aqp4	agtgacagagctgcggcaagg	ttccagaaagcctgagtcca
Cav1	gacgcgcacaccaaggagatt	ctgaccgggttggttttgat
Cd44	gctacagcaagaagggcgagt	cctgatctccagtaggctgttc
Cdh5	gcccagccctacgaacctaaa	gggtgaagttgctgtcctcgt
Cldn5	tgtcgtgcgtggtgcagagt	tgctacccgtgccttaactgg
Des	accatcgcggctaagaacatc	atcatctcctgcttggcttgg
Glut1/Slc2a1	tcgtcgttggcatccttatt	gtagcagggctgggatgaaga
Kcnj8	gtccgctgtctgtgtgaccaa	gaagatgcagcccaacatgac
Mmp12	agcacatttcgcctctctgct	gcttccaccagaagaaccagtc
Ng2/Cspg4	gccttcacgatcaccatcctt	gcccgaatcattgtctgttcc
Occln	gtgaatggcaagcgatcatacc	tgcctgaagtcatccacactca
Plvap	cttcatcgccgctatcatcct	ccttggagcacactgccttct
Slc1a1	cattgctgttgactggctcct	catccatctgctccagctcct
Slc1a3	cggtcactgctgtcattgtgg	ccatcttcccggatgccttac
Tmem119	acccggtccttcacccagag	gccgggagtgacacagagtag

Supplementary Table 2. Modified neurological severity score (mNSS) criteria for neurological deficits in mice post ischemic stroke

**14-points modified Neurological Severity Score (mNSS)**
**Motor tests (motor function scores)**
Flexion: raising the mouse by the tail (normal=0; maximum=3)
1 Flexion of forelimb
1 Flexion of hindlimb
1 Head movement more than 10 ° to the vertical axis within 30 seconds
Gait: placing the mouse on the floor (normal=0; maximum=3)
0 Normal walk
1 Inability to walk straight
2 Circling towards the paretic side
3 Falling towards the paretic side
**Beam balance tests (motor balance scores;** normal=0; maximum=6)
0 Balances with steady posture
1 Grasps side of beam
2 Hugs the beam and one limb falls down from the beam
3 Hugs the beam and two limbs fall down from the beam, or spins on beam (>30 seconds)
4 Attempts to balance on the beam but falls off (>20 seconds)
5 Attempts to balance on the beam but falls off (>10 seconds)
6 Falls off; no attempt to balance or hang on to the beam (<10 seconds)
**Sensory function tests** (**reflexes scores;** normal=0; maximum=2)
1 Absence of corneal reflex
1 Absence of Pinna reflex

Supplementary Table 3. Patient characteristics of stroke specimen used for osteopontin expression analysis

	Case ID	Age (years)	Gender	Localization of stroke lesion
**Stroke stage I (acute necrosis)**	1	58	female	Occipital lobe, bilateral
	2	50	female	Basal ganglia, right
	3	61	male	Multilocular
	4	62	male	Parietal/occipital lobe, left
	5	80	male	Frontal/temporal lobe, right
	6	74	female	Occipital lobe, right
**Stroke stage II (macrophage resorption)**	10	53	male	Temporal lobe, left
	12	74	female	Temporal lobe, left
	13	71	male	Parietal lobe, right
	15	87	female	Basal ganglia, right
	16	70	male	Cerebellum, left
	17	79	male	Occipital lobe, right
**Stroke stage III (pseudocystic cavity)**	20	76	male	Cerebellum, left
	21	67	male	Basal ganglia, left
	22	71	male	Basal ganglia, left
	23	70	male	Basal ganglia, left
	25	75	male	Frontal/temporal lobe, left
	26	73	male	Cerebellum, left

Supplementary Table 4. Antibody details for immunohistochemical and immunofluorescence staining

Antibody (species)	Company	Reference	Dilution
Albumin (Rb), poly	Proteintech	16475-1-AP	1/400
CD4 (Rb), mono	Abcam	ab183685	1/450
CD8a (Rb), mono	Cell Signaling	98941S	1/450
CD13 (Gt), poly	R&D systems	AF2335	1/200
CD31 (Ms), mono	Dako	Clone JC70A	1/200
Collagen IV (Rb), poly	BioRad	2150-1410	1/50
Claudin-5 (Ms), mono	ThermoFisher Scientific	352500	1/200
α-dystroglycan (Ms), mono	Novus biologicals	NBP1-49634	1/50
Fibrinogen (Rb), poly	LSBio	LS C150799	1/200
GFAP (Gt), poly	Abcam	ab53554	1/250
Iba1 (Ms), mono	Merck	SAB2702364	1/200
Iba1 (Rb), poly	Fujifilm Wako Chemicals	019-19741	1/100
Osteopontin (Rb), poly	Proteintech	22952-1-AP	1/500
PDGFRβ (Gt), poly	R&D systems	AF385	1/50
Podocalyxin (Rt), mono	R&D systems	MAB1556	1/200
VE-Cadherin (Gt), poly	R&D systems	AF1002	1/100
Dk anti-Gt 488, poly	ThermoFisher Scientific	SA5-10086	1/200
Dk anti-Gt 650, poly	ThermoFisher Scientific	SA5-10089	1/200
Dk anti-Rb 550, poly	ThermoFisher Scientific	SA5-10039	1/200
Dk anti-Rb 488, poly	ThermoFisher Scientific	SA5-10038	1/200
Dk anti-Ms 650, poly	ThermoFisher Scientific	SA5-10169	1/200
Dk anti-Ms 550, poly	ThermoFisher Scientific	SA5-10167	1/200
Dk anti-Rt 488, poly	ThermoFisher Scientific	SA5-10026	1/200
Gt anti-Rt 568, poly	ThermoFisher Scientific	A11077	1/200

Species: Rb, rabbit; Gt, goat; Ms, mouse; Rt, rat; Dk, donkey; mono, monoclonal; poly, polyclonal.

Supplementary Table 5. Overview of methods available for isolation and analysis of NVU cells in health and CNS disease

Cell type(s) isolated (species)	Cell isolation technique	Downstream application	Condition
EC (mouse)	Gradient centrifugation	Single-cell RNA-seq	Cognitive impairment[1]
EC (mouse, human)	LCM	Single-cell RNA-seq	Health[2]
EC (transgenic mouse)	FACS	RNA-Seq	Health and aging[3]
EC (transgenic mouse)	Ribosome IP	RNA-seq	Health[4]
EC (transgenic mouse)	FACS	Single-cell RNA-seq	Pericyte deficiency[5]
EC (transgenic mouse)	FACS	RNA-seq	Stroke, Multiple Sclerosis, Brain injury, seizure[6]
EC (transgenic mouse)	FACS	RNA-seq, MethylC-seq, ATAC-seq	Health[7]
EC and PC (mouse)	FACS	RNA-seq	Health[8]
AC (mouse)	FACS	Single-cell RNA-seq	Health[9]
AC and MG (mouse)	FACS	RNA-seq	Alzheimer's disease[10]
AC (transgenic mouse)	LCM	Single-cell RNA-seq	Health[11]
AC (transgenic mouse)	TRAP	RNA-seq	Cognitive impairment[12]
AC (transgenic mouse)	Ribosome IP	RNA-seq	Cognitive impairment[13]
MG (human)	FACS	Bulk/Single-cell RNA-seq	Alzheimer's disease[14]
MG (human)	FACS	Single-cell RNA-seq	Alzheimer's disease[15]
MG (mouse)	FACS	Single-cell RNA-seq	Cognitive impairment, brain injury[16]
Mural cells (transgenic mouse)	FACS	Single-cell RNA-seq	Health[17]
EC, mural cells, AC and MG*	FACS	Single-cell RNA-seq	Health[18]
EC, PC, MG, AC, OD and N*	FACS	RNA-seq	Health[19]

EC, endothelial cells; PC, pericytes; AC, astrocytes; MG, microglia; OD, oligodendrocytes; N, neurons; LCM, Laser capture microdissection; IP, Immunoprecipitation; FACS, Fluorescence-activated cell sorting; TRAP, Translating ribosome affinity purification; * indicates that cells were isolated from cell-specific reporter mice.

References

1. Kiss T, Nyúl-Tóth Á, Balasubramanian P, et al. Single-cell RNA sequencing identifies senescent cerebromicrovascular endothelial cells in the aged mouse brain. *GeroScience*. 2020;42(2):429-444. doi:10.1007/s11357-020-00177-1

2. Song HW, Foreman KL, Gastfriend BD, Kuo JS, Palecek SP, Shusta E V. Transcriptomic comparison of human and mouse brain microvessels. *Sci Rep*. 2020;10(1):12358. doi:10.1038/s41598-020-69096-7

3. Guérit S, Fidan E, Macas J, et al. Astrocyte-derived Wnt growth factors are required for endothelial blood-brain barrier maintenance. *Prog Neurobiol*. Published online December 2020:101937. doi:10.1016/j.pneurobio.2020.101937

4. Jambusaria A, Hong Z, Zhang L, et al. Endothelial heterogeneity across distinct vascular beds during homeostasis and inflammation. *Elife*. 2020;9. doi:10.7554/eLife.51413

5. Andaloussi Mäe M, He L, Nordling S, et al. Single-Cell Analysis of Blood-Brain Barrier Response to Pericyte Loss. *Circ Res*. Published online December 2020. doi:10.1161/CIRCRESAHA.120.317473

6. Munji RN, Soung AL, Weiner GA, et al. Profiling the mouse brain endothelial transcriptome in health and disease models reveals a core blood-brain barrier dysfunction module. *Nat Neurosci*. 2019;22(11):1892-1902. doi:10.1038/s41593-019-0497-x

7. Sabbagh MF, Heng JS, Luo C, et al. Transcriptional and epigenomic landscapes of CNS and non-CNS vascular endothelial cells. *Elife*. 2018;7. doi:10.7554/eLife.36187

8. Crouch EE, Doetsch F. FACS isolation of endothelial cells and pericytes from mouse brain microregions. *Nat Protoc*. 2018;13(4):738-751. doi:10.1038/nprot.2017.158

9. Batiuk MY, Martirosyan A, Wahis J, et al. Identification of region-specific astrocyte subtypes at single cell resolution. *Nat Commun*. 2020;11(1):1220. doi:10.1038/s41467-019-14198-8

10. Pan J, Ma N, Yu B, Zhang W, Wan J. Transcriptomic profiling of microglia and astrocytes throughout aging. *J Neuroinflammation*. 2020;17(1):97. doi:10.1186/s12974-020-01774-9

11. Mizrak D, Levitin HM, Delgado AC, et al. Single-Cell Analysis of Regional Differences in Adult V-SVZ Neural Stem Cell Lineages. *Cell Rep*. 2019;26(2):394-406.e5. doi:10.1016/j.celrep.2018.12.044

12. Clarke LE, Liddelow SA, Chakraborty C, Münch AE, Heiman M, Barres BA. Normal aging induces A1-like astrocyte reactivity. *Proc Natl Acad Sci U S A*. 2018;115(8):E1896-E1905. doi:10.1073/pnas.1800165115

13. Boisvert MM, Erikson GA, Shokhirev MN, Allen NJ. The Aging Astrocyte Transcriptome from Multiple Regions of the Mouse Brain. *Cell Rep*. 2018;22(1):269-285. doi:10.1016/j.celrep.2017.12.039

14. Alsema AM, Jiang Q, Kracht L, et al. Profiling Microglia From Alzheimer's Disease Donors and Non-demented Elderly in Acute Human Postmortem Cortical Tissue. *Front Mol Neurosci*. 2020;13:134. doi:10.3389/fnmol.2020.00134

15. Olah M, Menon V, Habib N, et al. Single cell RNA sequencing of human microglia uncovers a subset associated with Alzheimer's disease. *Nat Commun*. 2020;11(1):6129. doi:10.1038/s41467-020-19737-2

16. Hammond TR, Dufort C, Dissing-Olesen L, et al. Single-Cell RNA Sequencing of Microglia throughout the Mouse Lifespan and in the Injured Brain Reveals Complex Cell-State Changes. *Immunity*. 2019;50(1):253-271.e6. doi:10.1016/j.immuni.2018.11.004

17. He L, Vanlandewijck M, Raschperger E, et al. Analysis of the brain mural cell transcriptome. *Sci Rep*. 2016;6:35108. doi:10.1038/srep35108

18. Vanlandewijck M, He L, Mäe MA, et al. A molecular atlas of cell types and zonation in the brain vasculature. *Nature*. 2018;554(7693):475-480. doi:10.1038/nature25739

19. Zhang Y, Chen K, Sloan SA, et al. An RNA-sequencing transcriptome and splicing database of glia, neurons, and vascular cells of the cerebral cortex. *J Neurosci*. 2014;34(36):11929-11947. doi:10.1523/JNEUROSCI.1860-14.2014

Suppl Figure 1

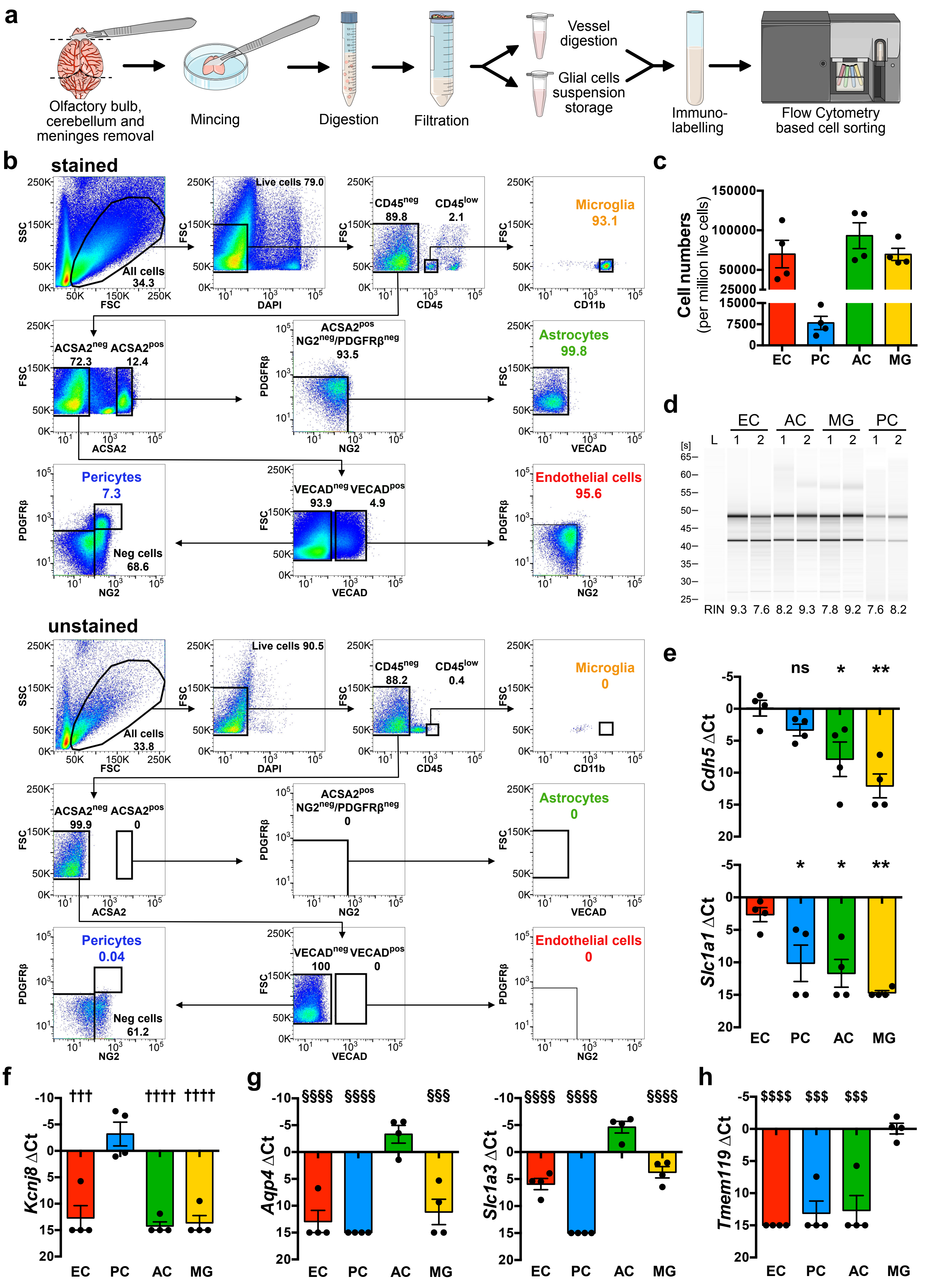

Supplementary Figure 1. Syngenic isolation of NVU cells from healthy adult mouse brain tissue. (**a**) Scheme depicting the sequence of mechanical tissue dissociation, filtration, cell type-specific enzymatic digestions, multiple immunolabeling and fluorescence activated cell sorting (FACS) for syngenic isolation of NVU cells in wild-type healthy animals. (**b**) Gating strategy allowing the simultaneous separation and collection of endothelial cells (EC), pericytes (PC), astrocytes (AC) and microglia (MG) from healthy mouse cerebrum. First FSC/SSC plot shows 1,000,000 events for stained and 100,000 for unstained samples. FSC, forward-scatter area; SSC, side-scatter area. Percentages refer to the proportion of cells in the previous parent gate. (**c**) Normalized cell numbers of endothelial cells, pericytes, astrocytes and microglia isolated by flow cytometry after tissue dissociation (n = 4, 2-3 mice/preparation). (**d**) Qualitative analysis of RNA from isolated endothelial cells, astrocytes, microglia and pericytes, (n = 2 independent isolations). (**e-h**) Purity of sorted cells was assessed by qRT-PCR, targeting cell type-specific markers for endothelial cells (**e**, *Cdh5*, *Slc1a1*), pericytes (**f**, *Kcnj8*), astrocytes (**g**, *Aqp4*, *Slc1a3*) and microglia (**h**, *Tmem119*). If no amplification was detected, the ΔCt value was set at 15 by default. * $P < 0.05$, ** $P < 0.01$, §§§/†††/$$$ $P < 0.001$, §§§§/††††/$$$$ $P < 0.0001$ and not significant (ns) $P > 0.05$ (n = 4) determined by one-way analysis of variance and followed by Dunnett’s multiple comparison test. *, †, § and $ indicate comparison to endothelial cells, pericytes, astrocytes and microglia, respectively.

Suppl Figure 2

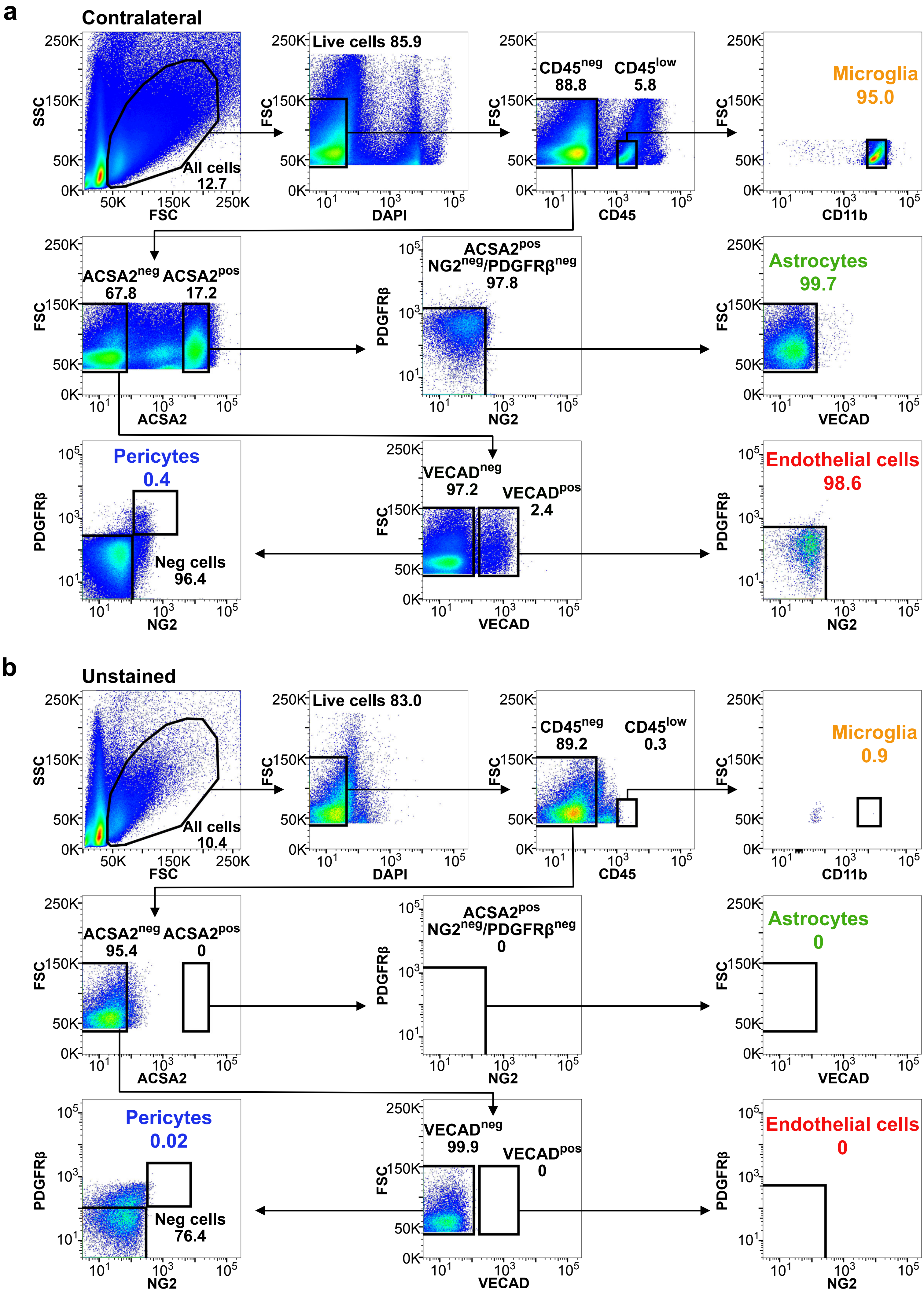

Supplementary Figure 2. Syngenic isolation of NVU cells from the contralateral hemisphere of animals subjected to tMCAO. (**a**) Gating strategy allowing the simultaneous separation and collection of endothelial cells, pericytes, astrocytes and microglia from the contralateral hemisphere after transient middle cerebral artery occlusion (tMCAO). First FSC/SSC plot shows 1,000,000 events. (**b**) FACS dot plots showing unstained controls (mixed ipsilateral and contralateral hemispheres cell suspension) for the selected surface antigens. First FSC/SSC plot shows 100,000 events. FSC, forward-scatter area; SSC, side-scatter area. Percentages refer to the proportion of cells in the previous parent gate.

Suppl Figure 3

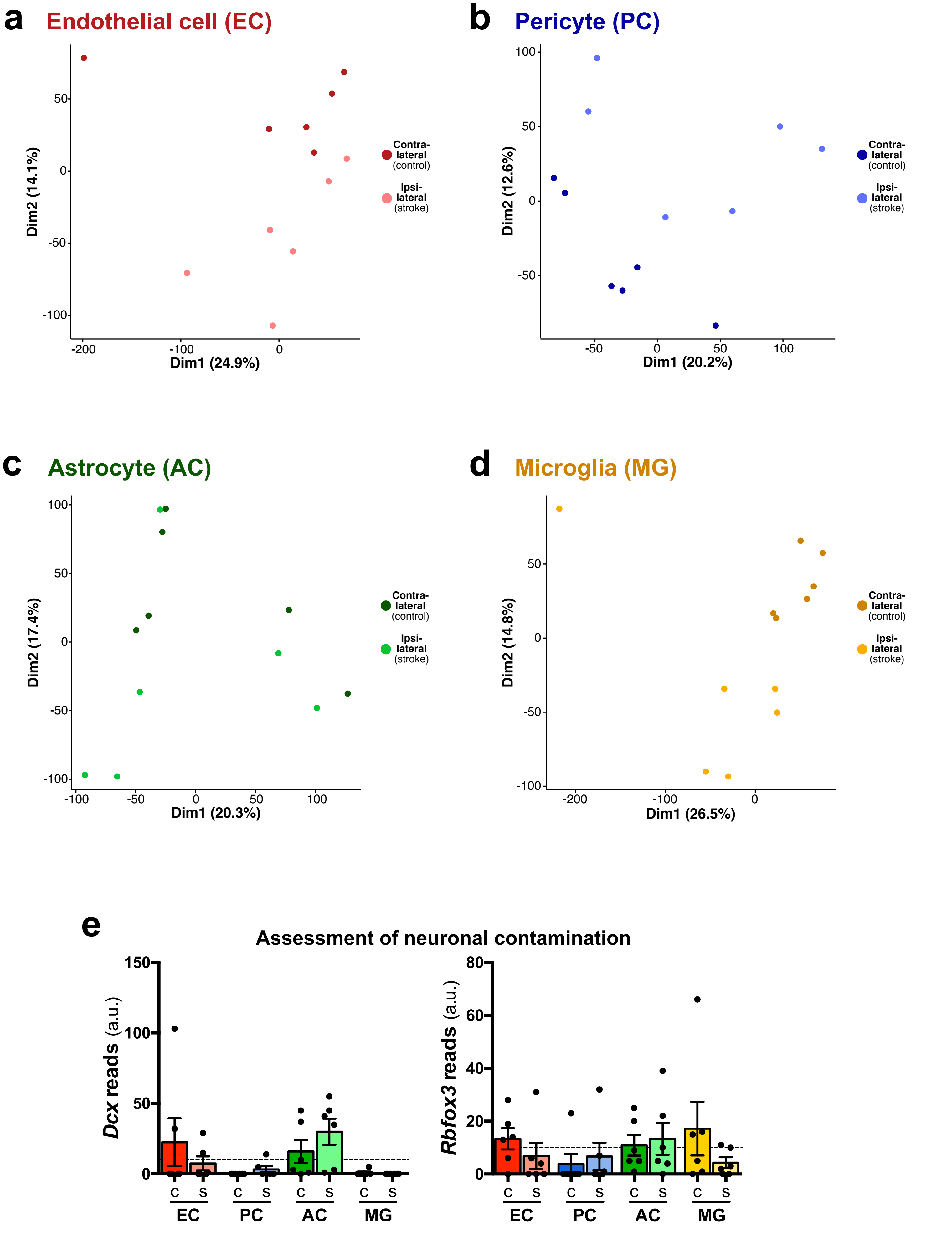

Supplementary Figure 3. Gene expression PCA plots of NVU cells isolated from murine stroke and contralateral hemisphere. (a-d) RNA-Sequencing principal component analysis (PCA) plot of NVU cell types isolated from the ischemic ipsilateral (stroke, light dots) and contralateral (control, dark dots) hemisphere (*n* = 6, 3-4 mice/preparation) including endothelial cells (**a**, red), pericytes (**b**, blue), astrocytes (**c**, green) and microglia (**d**, yellow). (**e**) Neuronal marker *Dcx* and *Rbfox3/NeuN* expression in endothelial cells, pericytes, astrocytes and microglia from the ischemic (stroke, s) and contralateral (c) hemispheres shows minimal neuronal contamination when isolating NVU cells using the EPAM-ia method. Dashed line represent the value of 10 reads, that is the threshold value to be considered as expressed.

Suppl Figure 4

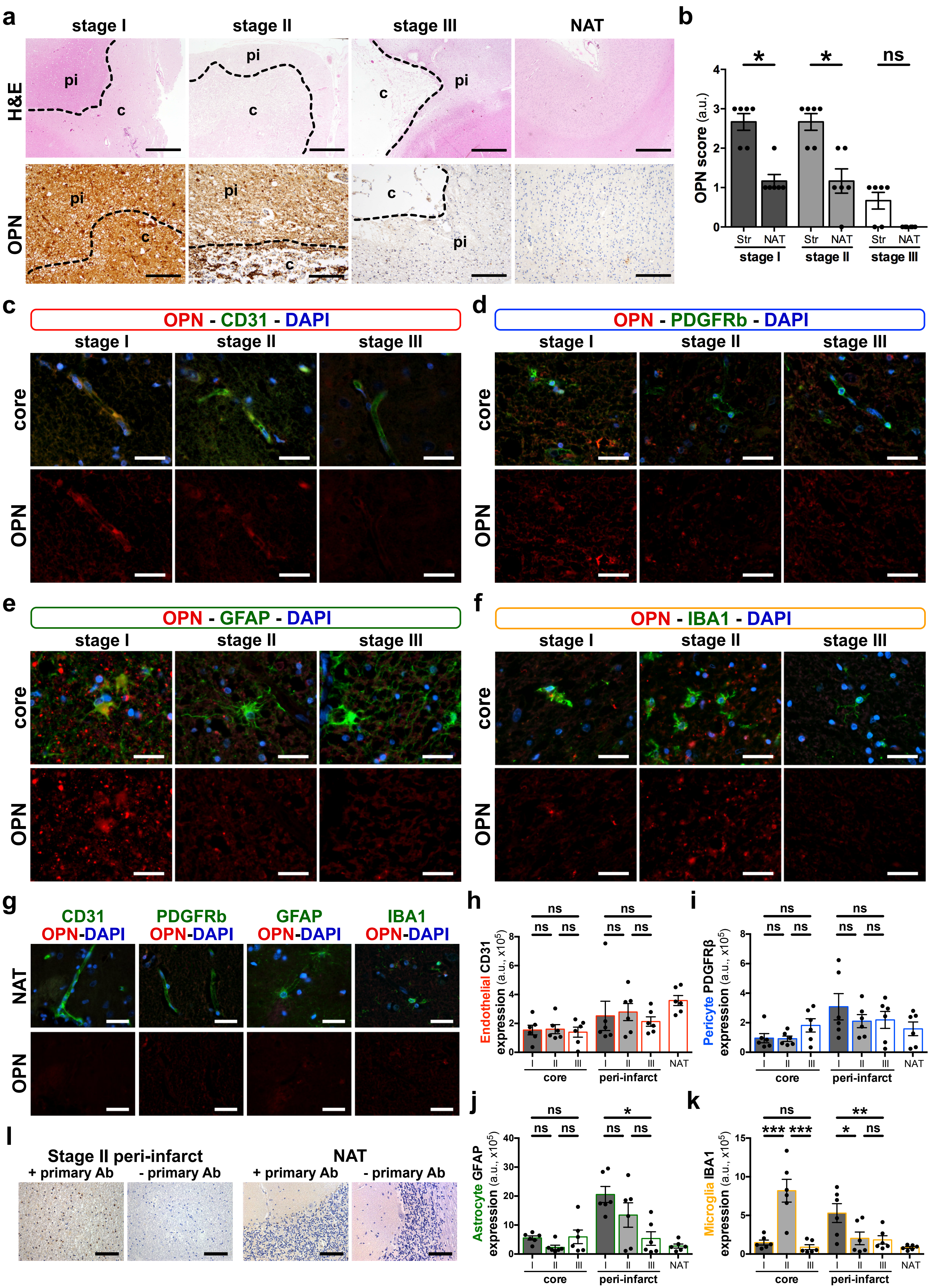

Supplementary Figure 4. Histopathological scoring of OPN levels and expression in normal and ischemic brain tissue of stroke patients. (**a**) Representative hematoxylin and eosin (H&E) staining (upper panel) and immunohistochemistry staining for osteopontin (OPN, brown; lower panel) of human stroke samples at different stages (stage I-III) identifying the peri-infarct region (pi) and infarct core (c) and normal appearing tissue (NAT). Dotted lines illustrate the border between the peri-infarct region and infarct core. (**b**) Neuropathological scoring of osteopontin levels (OPN, represented in **a,** lower panel) in the stroke area (Str), including the peri-infarct region and the infarct core, and NAT; n = 6 individual specimens for each stage, * $P < 0.05$ and not significant (ns) $P > 0.05$ by Wilcoxon test. (**c-g**) Representative images of immunofluorescence staining for osteopontin (OPN, red) and cell-specific markers (green) including CD31 for endothelial cells (**c, g**), PDGFRβ for pericytes (**d, g**), GFAP for astrocytes (**e, g**) and IBA1 for microglia/macrophages (**f, g**) in the infarct core (**c-f**) and the NAT (**g**) of human stroke samples. (**h-k**) Quantification of cell-specific markers including CD31 for endothelial cells (**h**), PDGFRβ for pericytes (**i**), GFAP for astrocytes (**j**) and IBA1 for microglia/macrophages (**k**) in the peri-infarct region, the infarct core, and the NAT of human stroke samples at stages I-III. n = 6 individual specimens for each stage, * P <0.05, ** P <0.01, *** P <0.001, and ns $P > 0.05$ by one-way analysis of variance and Tukey's multiple comparison test. (**l**) Representative images for anti-OPN primary antibody staining specificity in human stroke samples. Scale bars: 1000µm (**a**, upper panel), 200µm (**a**, lower panel), 20µm (**c-g**), and 100µm (**l**).

Suppl Figure 5

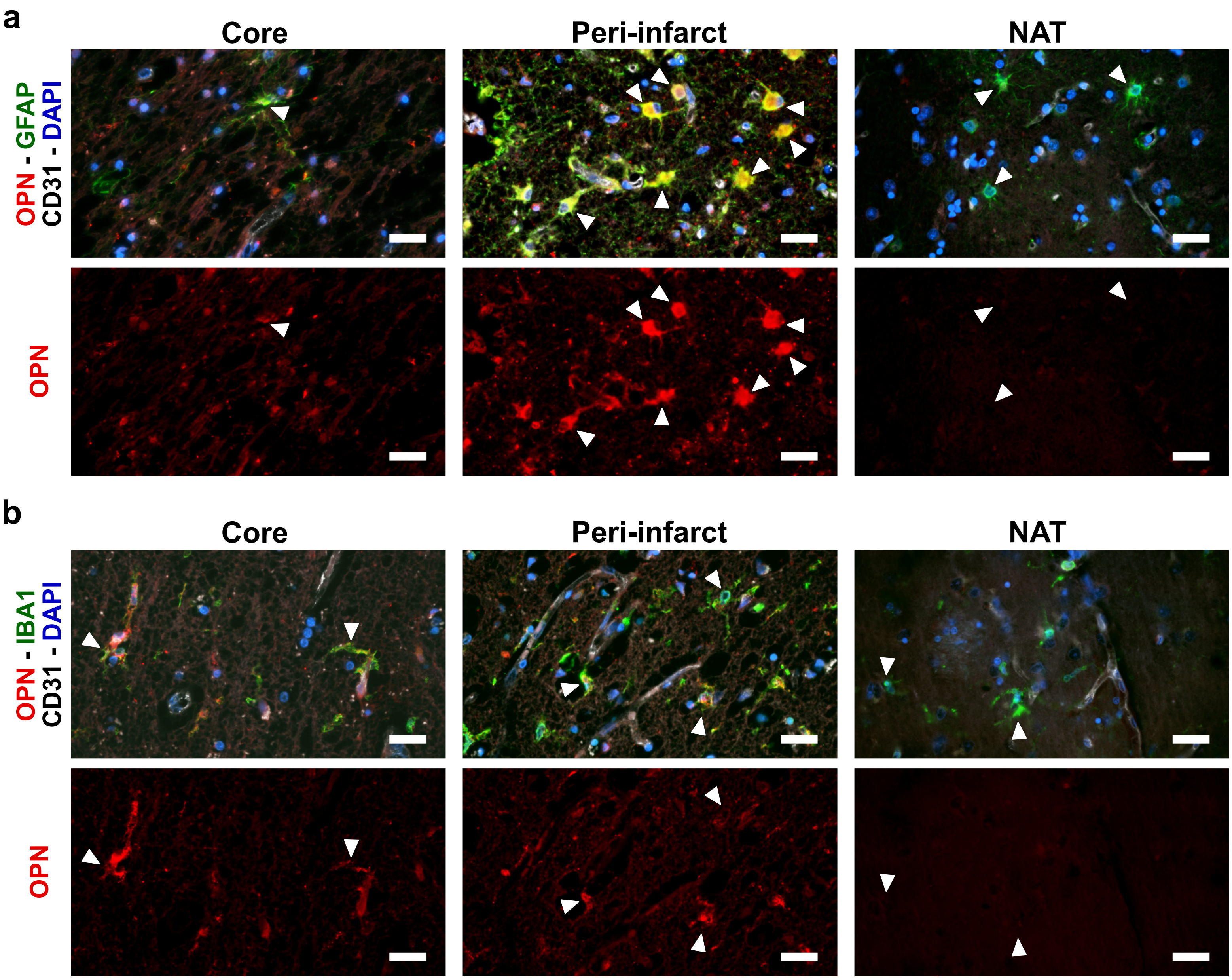

Supplementary Figure 5. Osteopontin expression in vessel-associated astrocytes and microglia/macrophages in normal and ischemic brain tissue of stroke patients. (**a**) Representative images of immunofluorescence staining for osteopontin (OPN, red) and astrocyte marker GFAP (green) together with vessel marker CD31 (white) in stage I human stroke tissue. (**b**) Representative images of immunofluorescence staining for osteopontin (OPN, red) and microglia/macrophages marker IBA1 (green) together with vessel marker CD31 (white) in stage I human stroke tissue. Arrowheads indicate vessel-associated astrocytes (**a**) and microglia/macrophages (**b**). Scale 20μm.

Suppl Figure 6

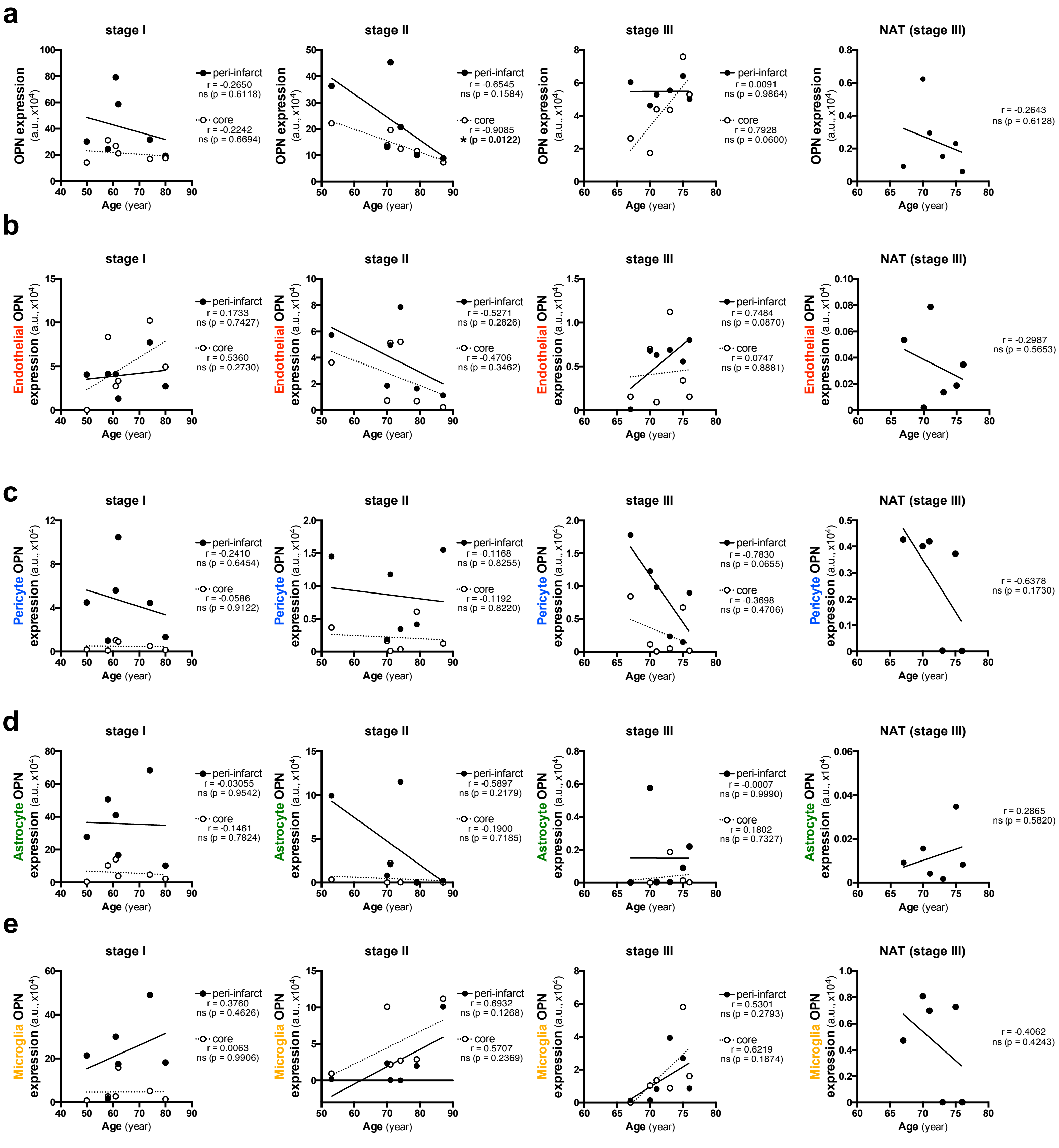

Supplementary Figure 6: Correlation analysis of OPN expression and age in human stroke samples. (**a**) Correlation analysis of total OPN expression (IHC-peroxidase) with age in peri-infarct (black dots) and core (white dots) human samples post ischemic tissue. (**b-e**) Correlation analysis of cell specific OPN expression (immunofluorescence) with age in peri-infarct (black dots) and core (white dots) endothelial cells (**b**), pericytes (**c**), astrocytes (**d**) and microglia/macrophages (**e**). n = 6 each group. P value was determined using Pearson's correlation test * $P < 0.05$, and not significant (ns) $P > 0.05$.

Suppl Figure 7

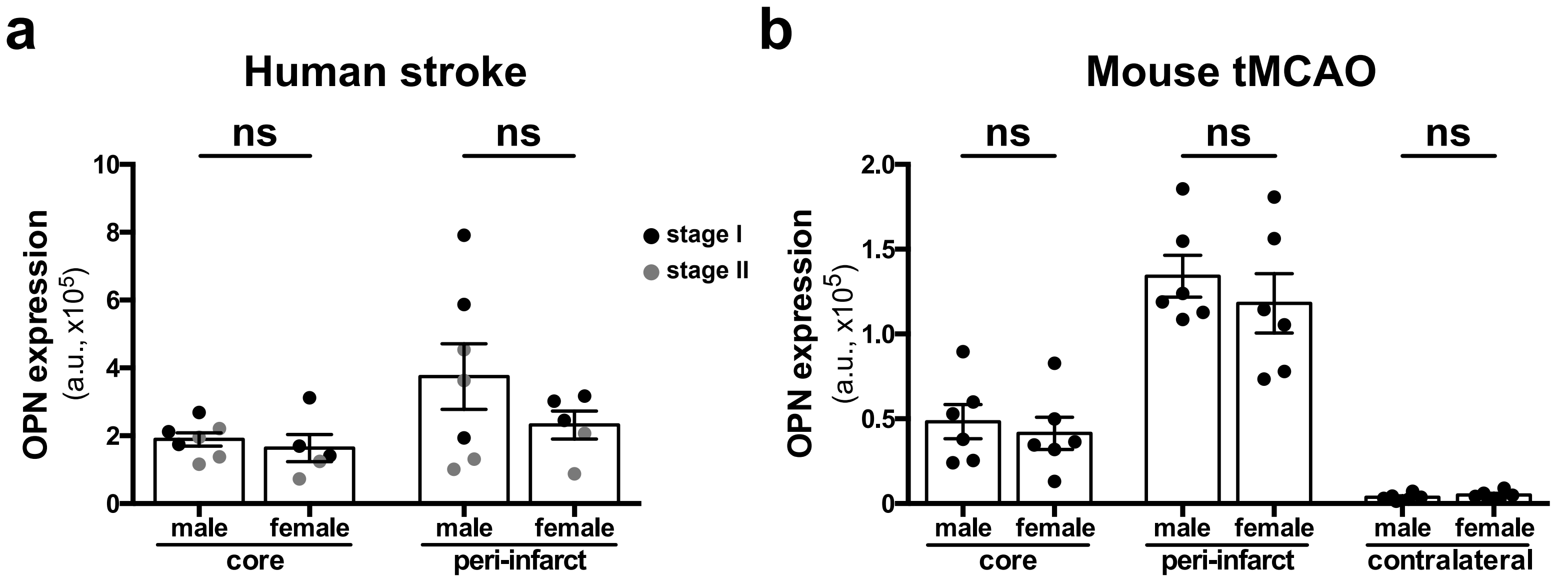

Supplementary Figure 7: Absence of gender-related influence on OPN expression in human and mouse ischemic tissues. (**a**) Quantification of OPN expression (IHC-peroxidase) in stage I (black dots) and II (grey dots) tissues in core and peri-infarct human male and female samples. n = 3 for both stage I males and females, n = 4 and 2 for stage II males and females, respectively. (**b**) Quantification of OPN expression (IHC-peroxidase) in males and females Ctrl IgG antibody-treated mice (n = 6 each group). ns $P > 0.05$ by two-tailed, unpaired t-test.

Suppl Figure 8

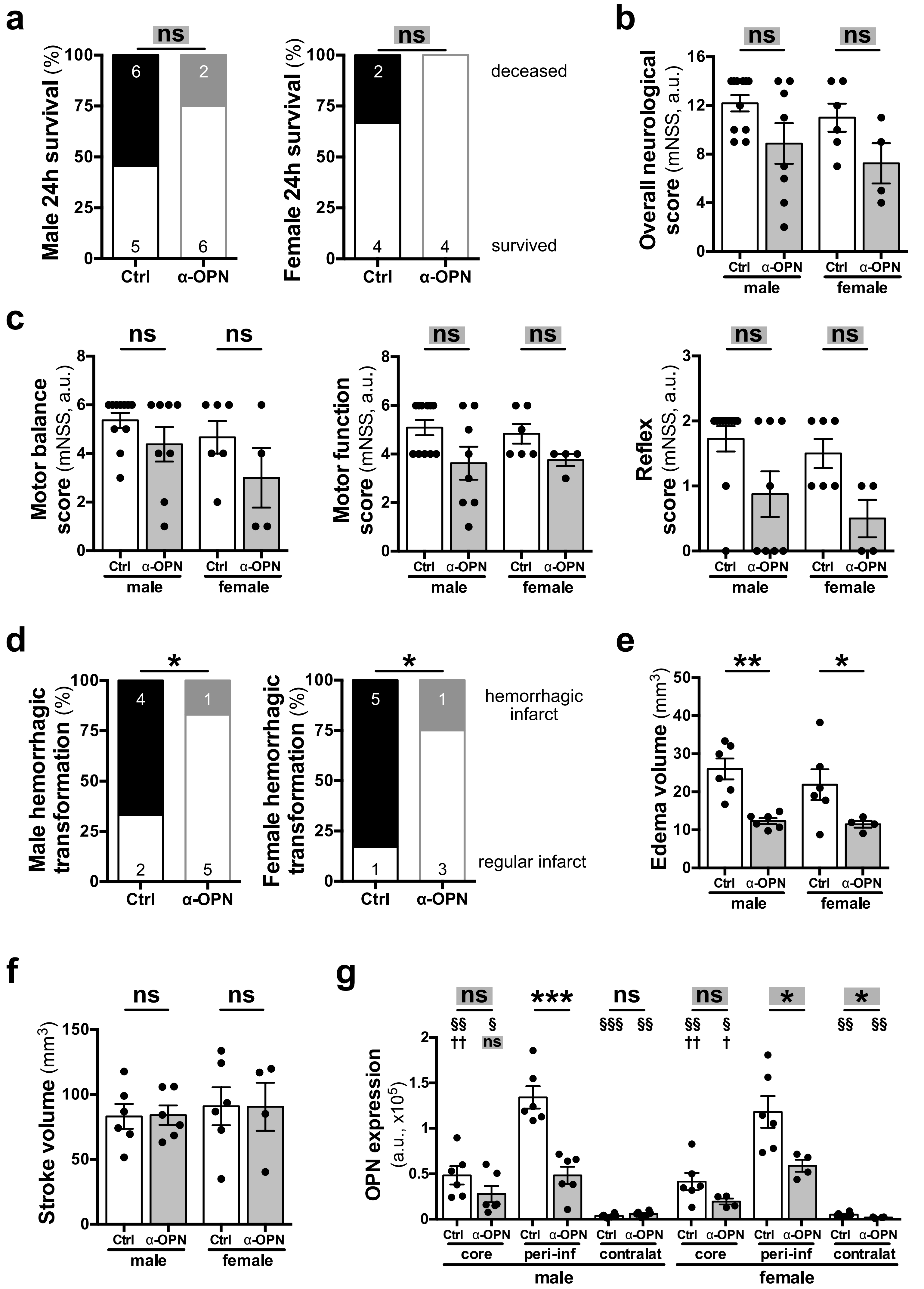

Supplementary Figure 8. Gender-related effects of anti-OPN antibody treatment on OPN expression and outcome in mice post ischemic stroke. Outcome parameters in control (Ctrl) IgG or anti (α)-OPN antibody treated male (Ctrl, $n = 11$; α-OPN, $n = 8$) and female (Ctrl, $n = 6$; α-OPN, $n = 4$) mice. For analysis of hemorrhagic transformation, edema and stroke volumes, and OPN expression, only mice that survived for 24 hours were included ($n = 6$ and 6 for Ctrl males and females, and $n = 6$ and 4 for α-OPN males and females, respectively). (**a**) 24 hours survival proportion with numbers in histograms indicating raw number of animals dead or surviving in each group. $n = 11$ and 8 for Ctrl IgG-treated males and females, and $n = 6$ and 4 for α-OPN antibody-treated males and females, respectively; not significant (ns) $P > 0.05$ by one-tailed Chi-square test. (**b**) Total mNSS including (**c**) motor balance, motor function and reflexes scores. $n = 11$ and 8 for Ctrl IgG-treated males and females, and $n = 6$ and 4 for α-OPN antibody-treated males and females, respectively; ns $P > 0.05$ by Mann Whitney test. (**d**) Hemorrhagic transformation frequency of stroke lesions. (**e**) Edema volume, (**f**) stroke volume, $n = 6$ for both Ctrl IgG treated males and females, and $n = 6$ and 4 for α-OPN antibody-treated males and females respectively; * $P < 0.05$ and ** $P < 0.01$ and ns $P > 0.05$ by one-tailed Chi-square test for (**d**) and by two-tailed, unpaired t-test, with Welch's correction when variances were significantly different based on F-test, for (**e**) and (**f**). (**g**) Quantification of OPN expression (arbitrary units, a.u.) in the infarct core, peri-infarct region and contralateral hemisphere (3 images/region/animal); $n = 6$ for both Ctrl IgG treated males and females, and $n = 6$ and 4 for α-OPN antibody-treated males and females, respectively; */§/† $p<0.05$, **/§§/†† $p<0.01$, ***/§§§ $p<0.001$ and ns $p>0.05$. * indicates two-tailed, unpaired t-test comparing the two treatment groups for the same region, § indicates two-tailed, paired t-test comparison of peri-infarct to contralateral or core regions within the same treatment group/animal, and † indicates two-tailed, paired t-test comparison of core to contralateral regions within the same treatment group/animal. Grey rectangles highlight the difference of significance observed in gender separated results from pooled results (Fig 5)

Suppl Figure 9

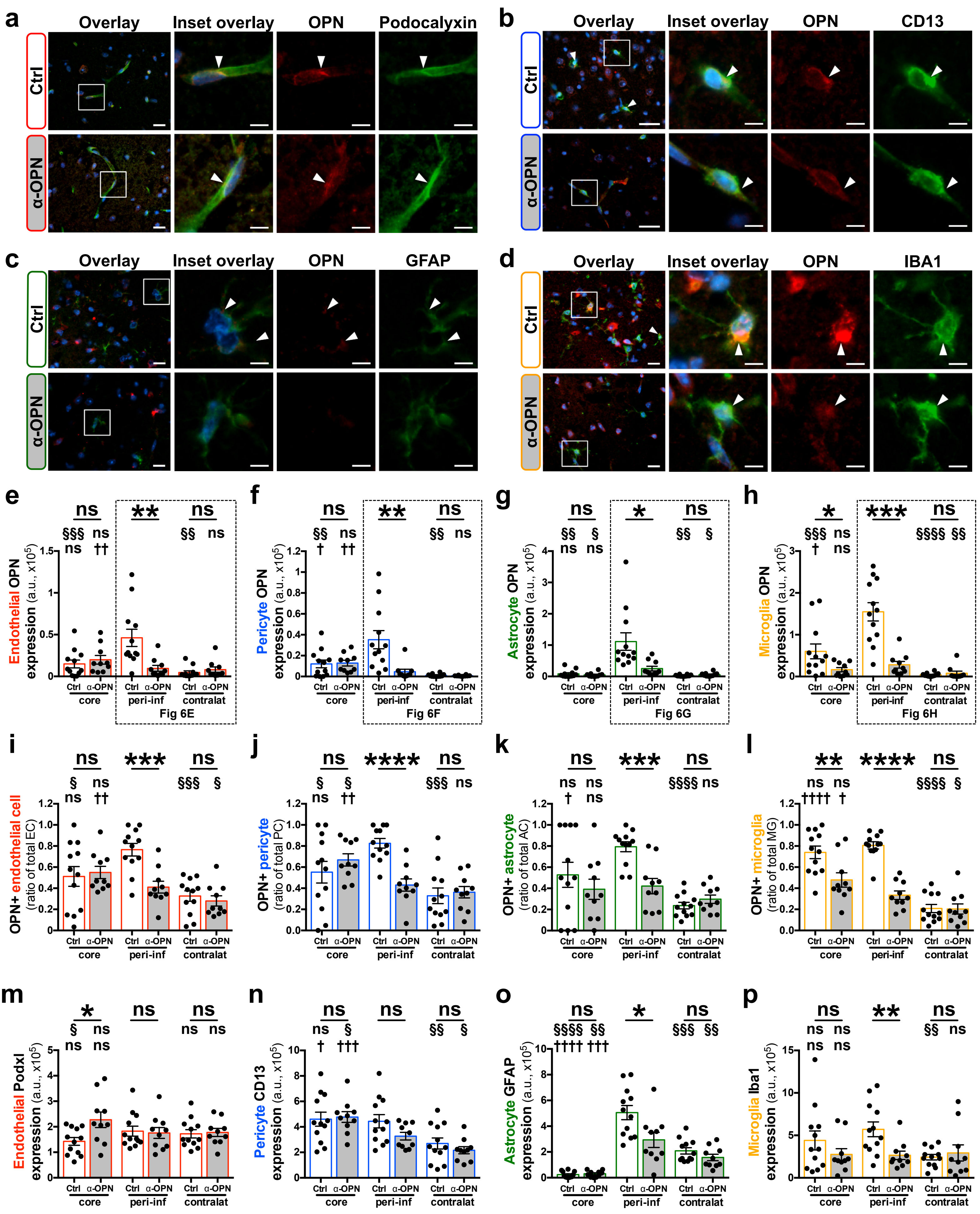

Supplementary Figure 9. OPN expression pattern of murine NVU cells in the infarct core, peri-infarct region and contralateral hemisphere after antibody treatment. (**a-d**) Representative images of immunofluorescence staining for osteopontin (OPN, red) and cell-specific markers (green) including podocalyxin for endothelial cells (**a**), CD13 for pericytes (**b**), GFAP for astrocytes (**c**) and IBA1 for microglia/macrophages (**d**) in the infarct core of Ctrl IgG and α-OPN antibody-treated mice 24 hours post ischemic stroke. White arrowheads indicate OPN expressing NVU cells. (**e-l**) Quantification of OPN expression in core, peri-infarct, and contralateral endothelial cells (**e**), pericytes (**f**), astrocytes (**g**) and microglia/macrophages (**h**) and ratio of OPN positive endothelial cells (**i**), pericytes (**j**), astrocytes (**k**) and microglia/macrophages (**l**) in Ctrl IgG and α-OPN antibody-treated mice. (**m-p**) Quantification (arbitrary units, a.u.) of podocalyxin (podxl) staining in endothelial cells (**m**) as well as CD13 in pericytes (**n**), GFAP in astrocytes (**o**) and IBA1 in microglia/macrophages (**p**) in the peri-infarct region and contralateral hemisphere of Ctrl IgG and α-OPN antibody-treated mice utilizing 3 images/region/animal, n = 12 and 10 for Ctrl and α-OPN, respectively. */§/† $P < 0.05$, **/§§/†† $P < 0.01$, ***/§§§/††† $P < 0.001$, ****/§§§§/†††† $P < 0.0001$ and not significant (ns) $P > 0.05$. * indicates two-tailed, unpaired t-test, with Welch's correction when variances were significantly different based on F-test, comparing the two treatment groups for the same region, § indicates two-tailed, paired t-test comparison of peri-infarct to contralateral or core regions within the same treatment group/animal, and † indicates two-tailed, paired t-test comparison of core to contralateral regions within the same treatment group/animal. Scale bars: 10μm (5μm in insets).

Suppl Figure 10

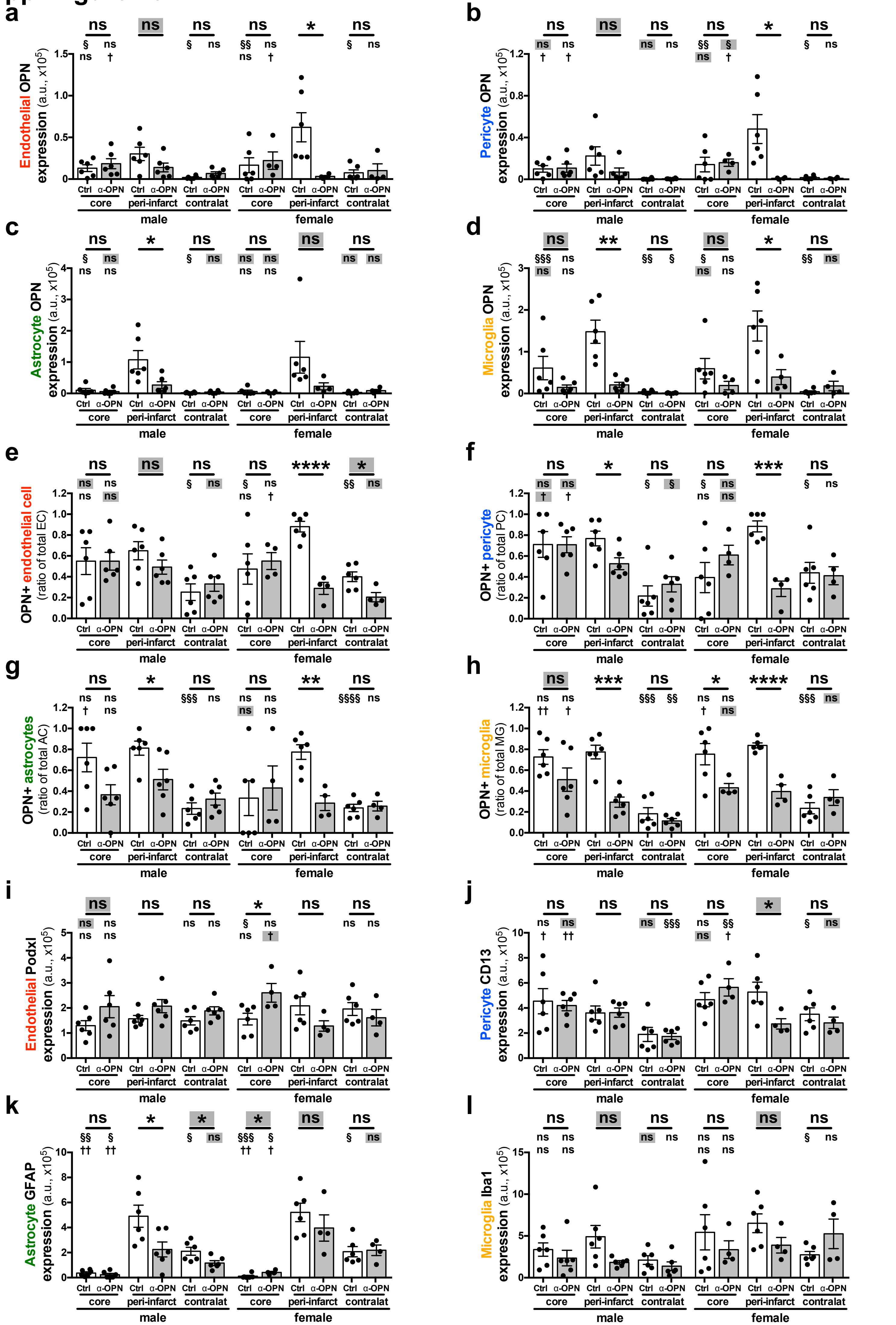

Supplementary Figure 10: Gender-related effects of anti-OPN antibody treatment on OPN expression pattern of murine NVU cells in the infarct core, peri-infarct region and contralateral hemisphere after antibody treatment. (**a-h**) Quantification of OPN expression in core, peri-infarct, and contralateral endothelial cells (**a**), pericytes (**b**), astrocytes (**c**) and microglia/macrophages (**d**) and ratio of OPN positive endothelial cells (**e**), pericytes (**f**), astrocytes (**g**) and microglia/macrophages (**h**) in males and females Ctrl IgG and α-OPN antibody-treated mice. (**i-l**) Quantification (arbitrary units, a.u.) of podocalyxin (Podxl) staining in endothelial cells (**i**) as well as CD13 in pericytes (**j**), GFAP in astrocytes (**k**) and IBA1 in microglia/macrophages (**l**) in core, peri-infarct region, and contralateral hemisphere of males and females Ctrl IgG and α-OPN antibody-treated mice. Three images per region and per animal were used, n = 6 for both Ctrl IgG treated males and females, and n = 6 and 4 for α-OPN antibody-treated males and females, respectively. */§/† $P < 0.05$, **/§§/†† $P < 0.01$, ***/§§§ $P < 0.001$, ****/§§§§ $P < 0.0001$ and not significant (ns) $P > 0.05$. * indicates two-tailed, unpaired t-test, with Welch's correction when variances were significantly different based on F-test, comparing the two treatment groups for the same region. § indicates two-tailed, paired t-test comparison of peri-infarct to contralateral or core regions within the same treatment group/animal, and † indicates two-tailed, paired t-test comparison of core to contralateral regions within the same treatment group/animal. Grey rectangles highlight the difference of significance observed in gender separated results from pooled results (Fig Suppl 9)

Suppl Figure 11

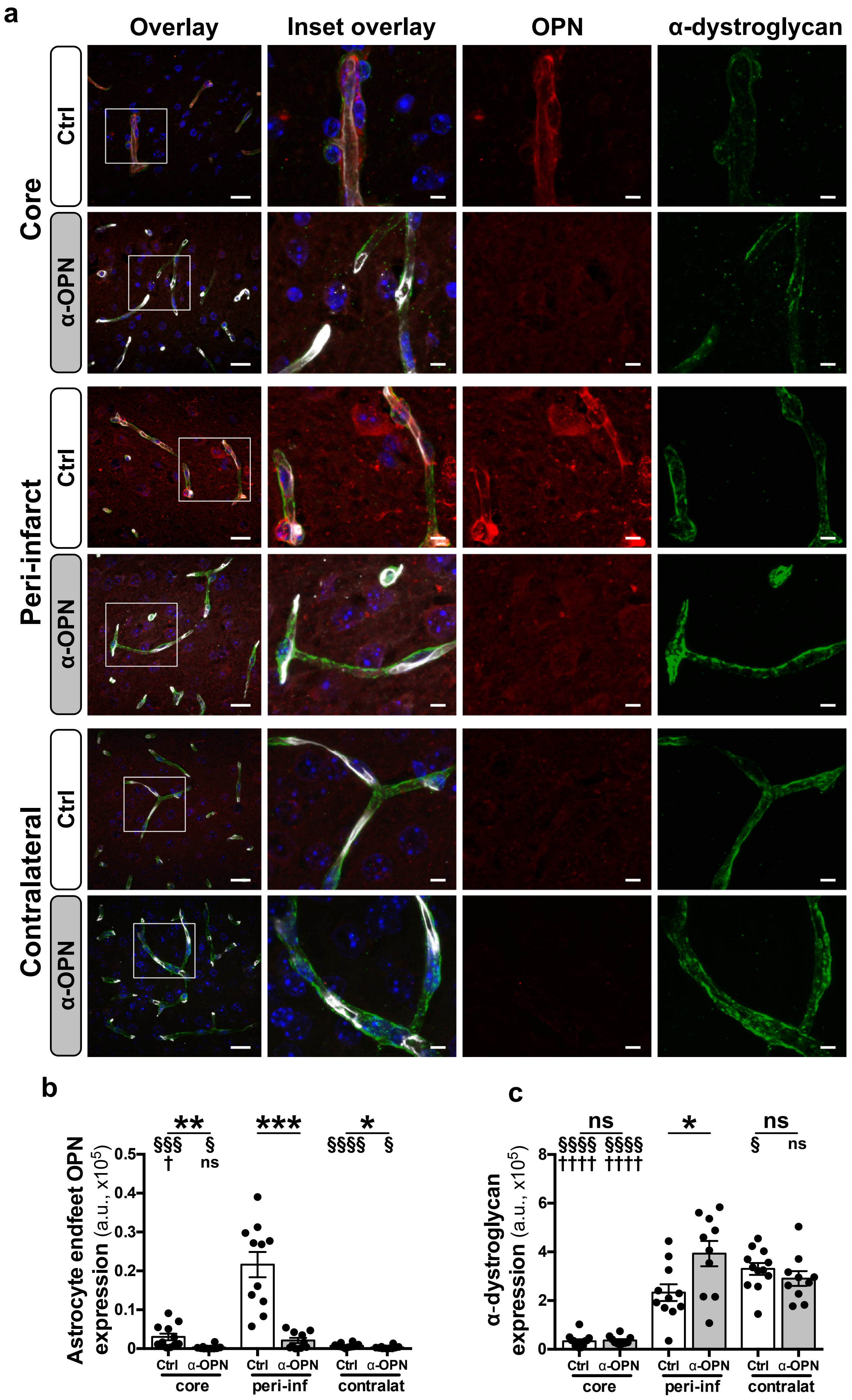

Supplementary Figure 11. Effect of the anti-OPN antibody therapy on astrocyte endfeet in ischemic mice. (**a**) Representative images of immunofluorescence staining for OPN (red) and astrocyte endfeet marker α-dystroglycan (green) in the ischemic core, peri-infarct and contralateral hemisphere of Ctrl IgG and α-OPN antibody-treated mice. Podocalyxin (white) was used as vessel marker as shown on overlay pictures (**b-c**) Quantification of OPN expression in astrocyte endfeet (**b**) and α-dystroglycan expression (**c**) in ischemic core, peri-infarct and contralateral hemisphere of Ctrl IgG and α-OPN antibody-treated mice. Quantifications were done utilizing three images/region/animal, n = 12 and n = 10 for Ctrl and α-OPN, respectively; */§/† $P < 0.05$, ** $P < 0.01$, ***/§§§ $P < 0.001$, §§§§/†††† $P < 0.0001$ and not significant (ns) $P > 0.05$. * indicates two-tailed, unpaired t-test, with Welch's correction when variances were significantly different based on F-test, comparing the two treatment groups for the same region. § indicates two-tailed, paired t-test comparison of peri-infarct to core or contralateral regions within the same treatment group/animal, and † indicates two-tailed paired t-test comparison of the infarct core and contralateral regions within the same treatment group/animal. Scale bars: 20μm and inset: 5μm.

Suppl Figure 12

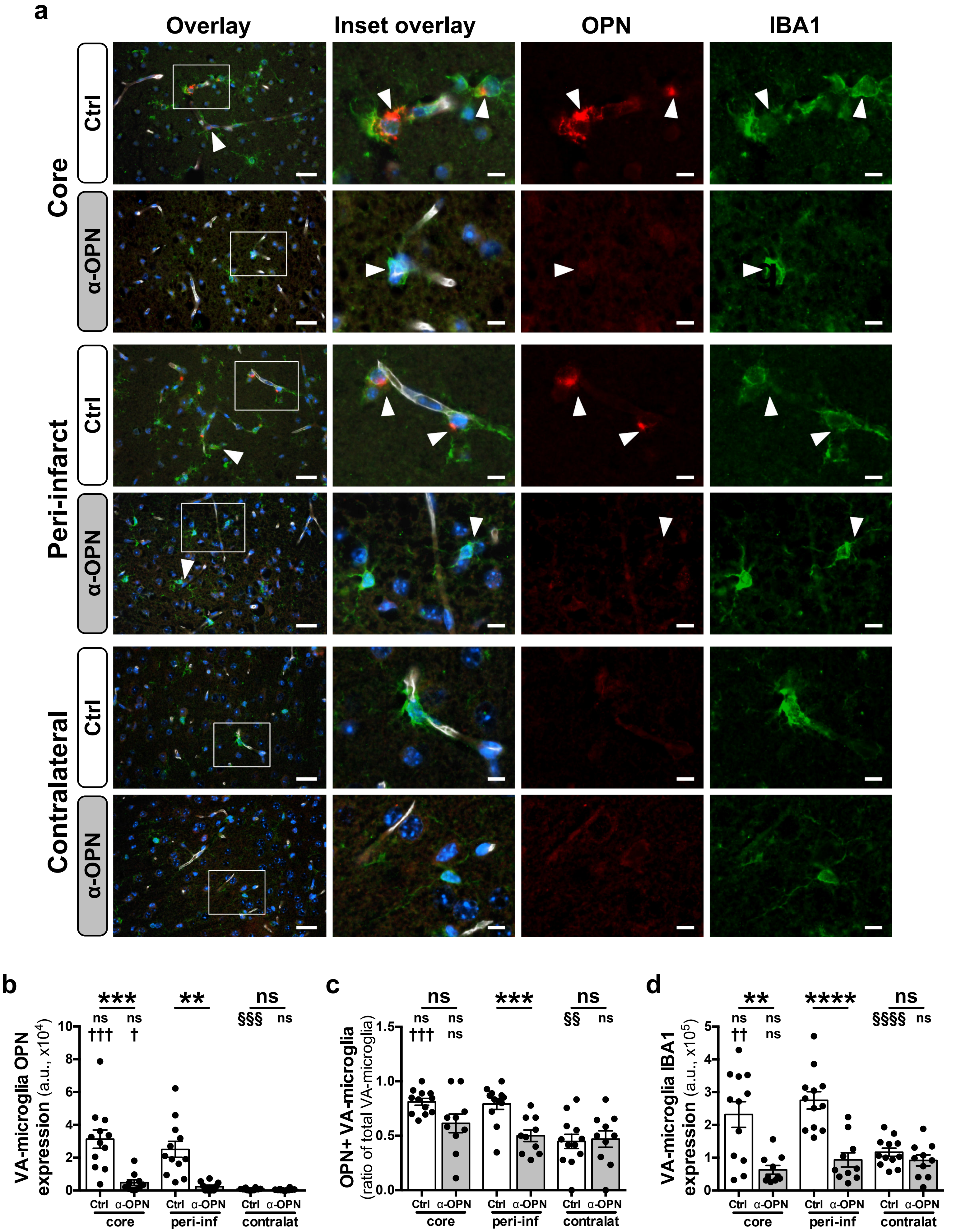

Supplementary Figure 12. Effect of the anti-OPN antibody therapy on vessel-associated microglia/macrophages in ischemic mice. (**a**) Representative images of immunofluorescence staining for OPN (red) and vessel-associated (VA) microglia/macrophages (IBA1, green) in the ischemic core, peri-infarct and contralateral hemisphere of Ctrl IgG and α-OPN antibody-treated mice. Podocalyxin (white) was used as vessel marker as shown on overlay pictures (**b-d**) Quantification of OPN expression (**b**), the ratio of vessel-associated microglia/macrophages positive for OPN (**c**), and IBA1 expression (**d**) in ischemic core, peri-infarct and contralateral hemisphere of Ctrl IgG and α-OPN antibody-treated mice. Quantifications were done utilizing three images/region/animal, $n = 12$ and $n = 10$ for Ctrl and α-OPN, respectively; † $P < 0.05$, **/§§/†† $P < 0.01$, ***/§§§/††† $P < 0.001$, ****/§§§§ $P < 0.0001$ and not significant (ns) $P > 0.05$. * indicates two-tailed, unpaired t-test, with Welch's correction when variances were significantly different based on F-test, comparing the two treatment groups for the same region. § indicates two-tailed, paired t-test comparison of peri-infarct to core or contralateral regions within the same treatment group/animal, and † indicates two-tailed paired t-test comparison of the infarct core and contralateral regions within the same treatment group/animal. Scale bars: 20μm and inset: 5μm.

Suppl Figure 13

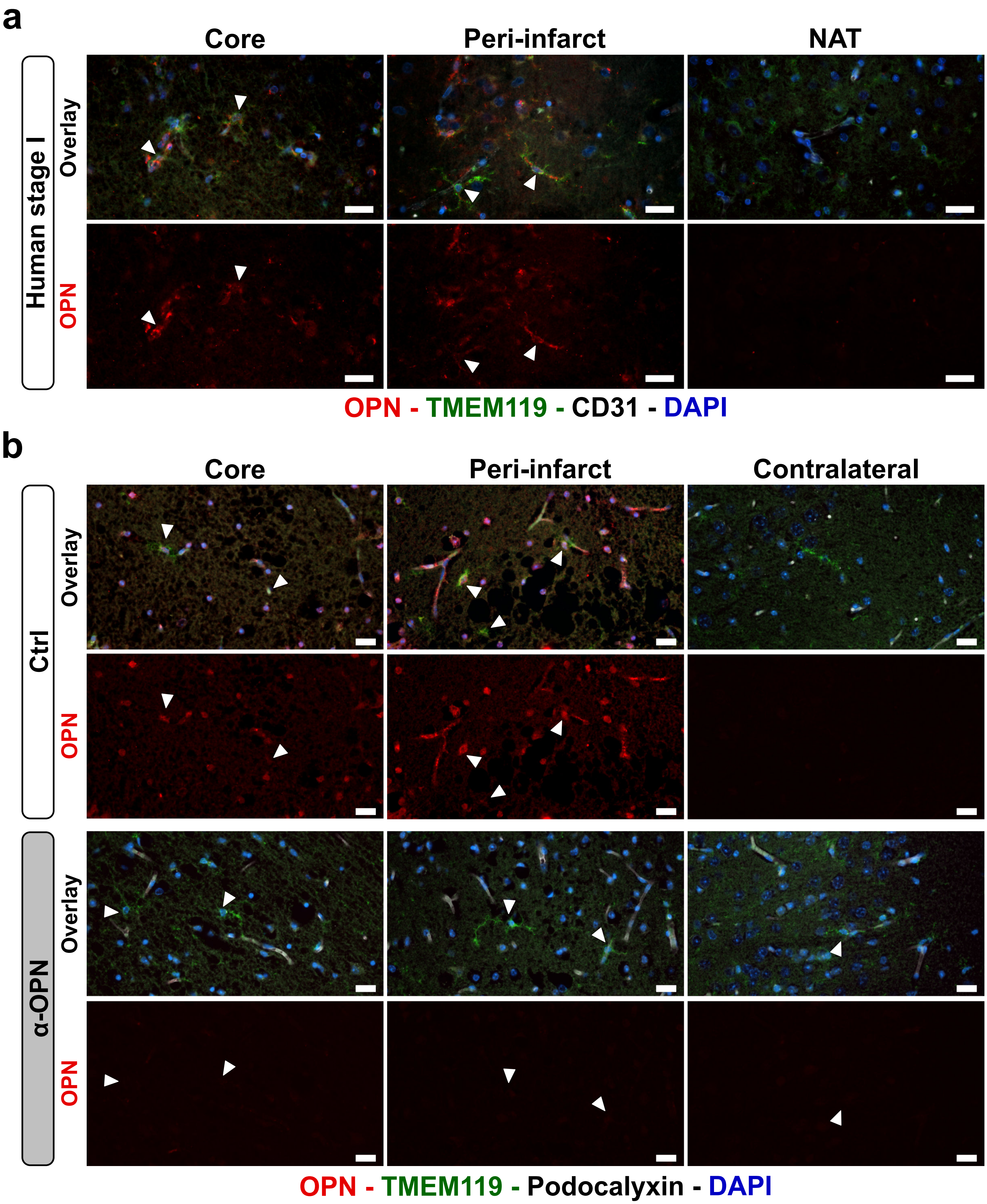

Supplementary Figure 13. Osteopontin expression in vessel-associated microglia in human and murine specimen post-acute ischemic stroke. (**a**) Representative images of immunofluorescence staining for osteopontin (OPN, red) and microglia marker TMEM119 (green) together with vessel marker CD31 (white) in stage I human stroke tissue and normal appearing tissue (NAT). (**b**) Representative images of immunofluorescence staining for osteopontin (OPN, red) and microglia marker TMEM119 (green) together with vessel marker Podocalyxin (white) in Ctrl IgG and α-OPN antibody-treated mice. Scale 20μm.

Suppl Figure 14

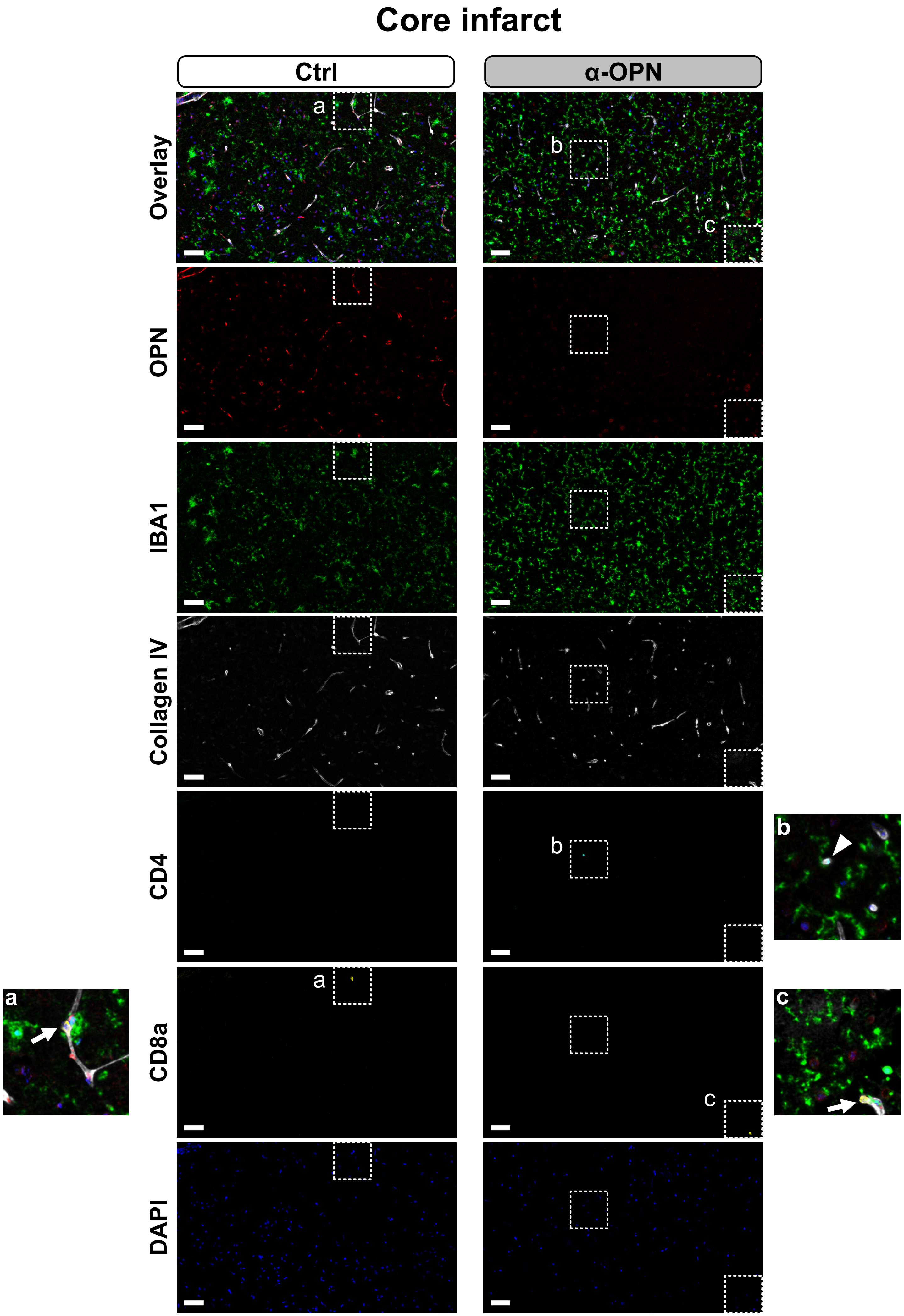

Supplementary Figure 14: Effect of the anti-OPN antibody therapy on immune cells infiltration in the infarct core post ischemic stroke in mice. Representative image (of n = 2) of immunofluorescence staining for osteopontin (OPN, red), microglia/macrophage marker IBA1 (green), vessel marker collagen IV (white) together with lymphocyte markers CD4 (cyan, arrowhead) and CD8a (yellow, arrow) in the infarct core of Ctrl IgG and α-OPN antibody-treated mice. DAPI was used to reveal nuclei. Scale 50μm.

Suppl Figure 15

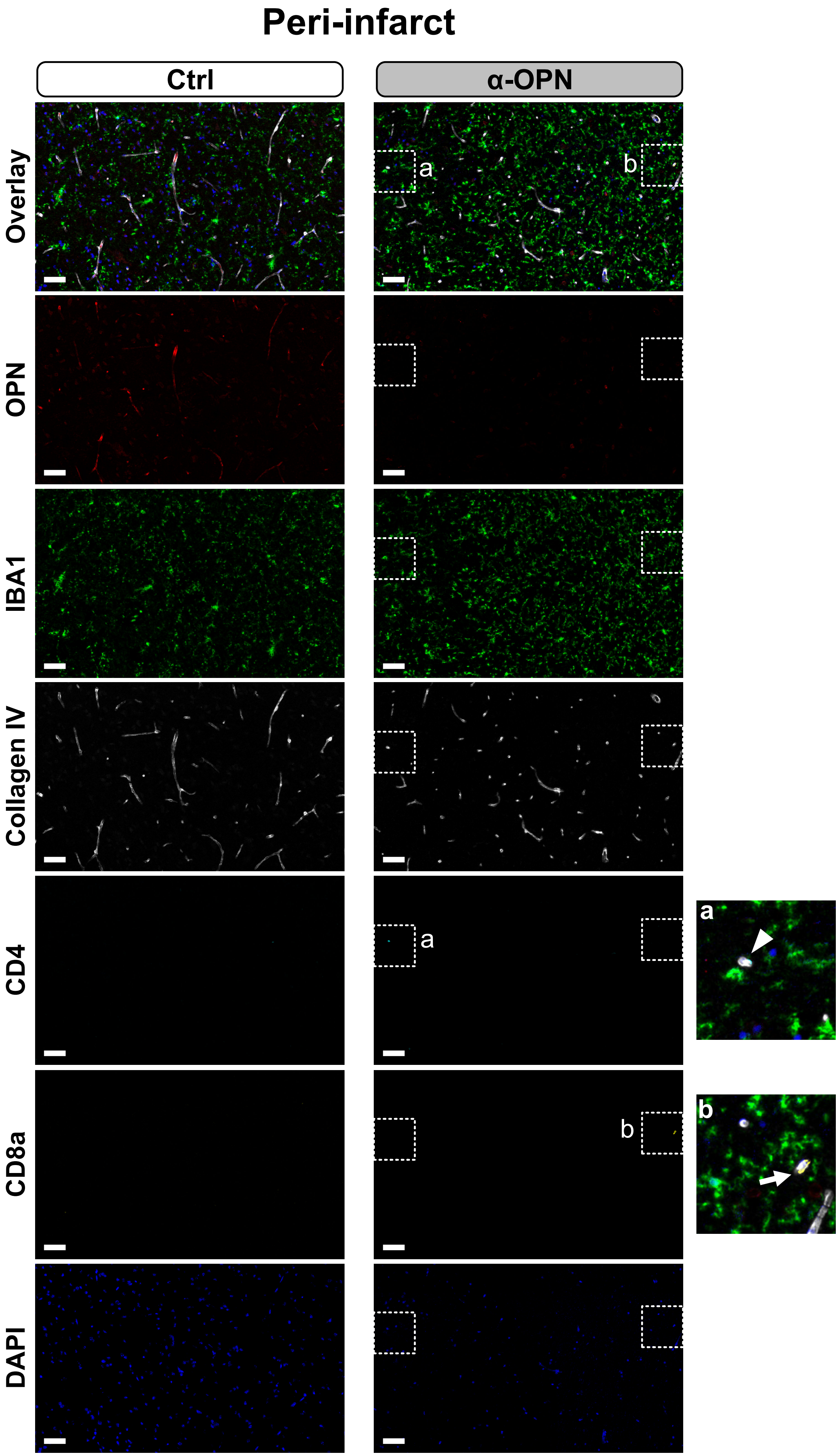

Supplementary Figure 15: Effect of the anti-OPN antibody therapy on immune cells infiltration in the peri-infarct region post ischemic stroke in mice. Representative image (of n = 2) of immunofluorescence staining for osteopontin (OPN, red), microglia/macrophage marker IBA1 (green), vessel marker collagen IV (white) together with lymphocyte markers CD4 (cyan, arrowhead) and CD8a (yellow, arrow) in the peri-infarct region of Ctrl IgG and α-OPN antibody-treated mice. DAPI was used to reveal nuclei. Scale 50µm.

Suppl Figure 16

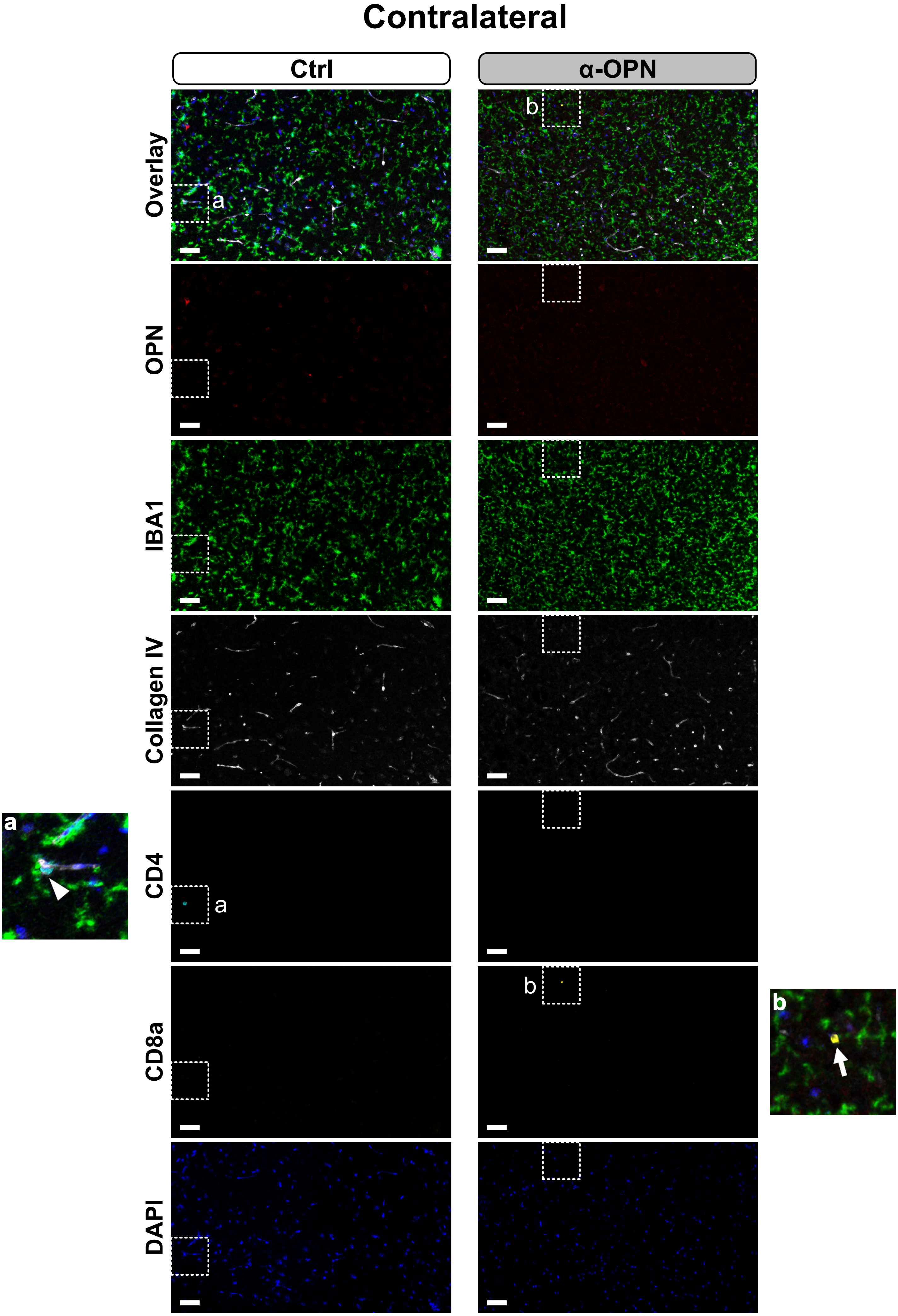

Supplementary Figure 16: Effect of the anti-OPN antibody therapy on immune cells infiltration in the contralateral hemisphere post ischemic stroke in mice. Representative image (of n = 2) of immunofluorescence staining for osteopontin (OPN, red), microglia/macrophage marker IBA1 (green), vessel marker collagen IV (white) together with lymphocyte markers CD4 (cyan, arrowhead) and CD8a (yellow, arrow) in the contralateral hemisphere of Ctrl IgG and α-OPN antibody-treated mice. DAPI was used to reveal nuclei. Scale 50μm.

Suppl Figure 17

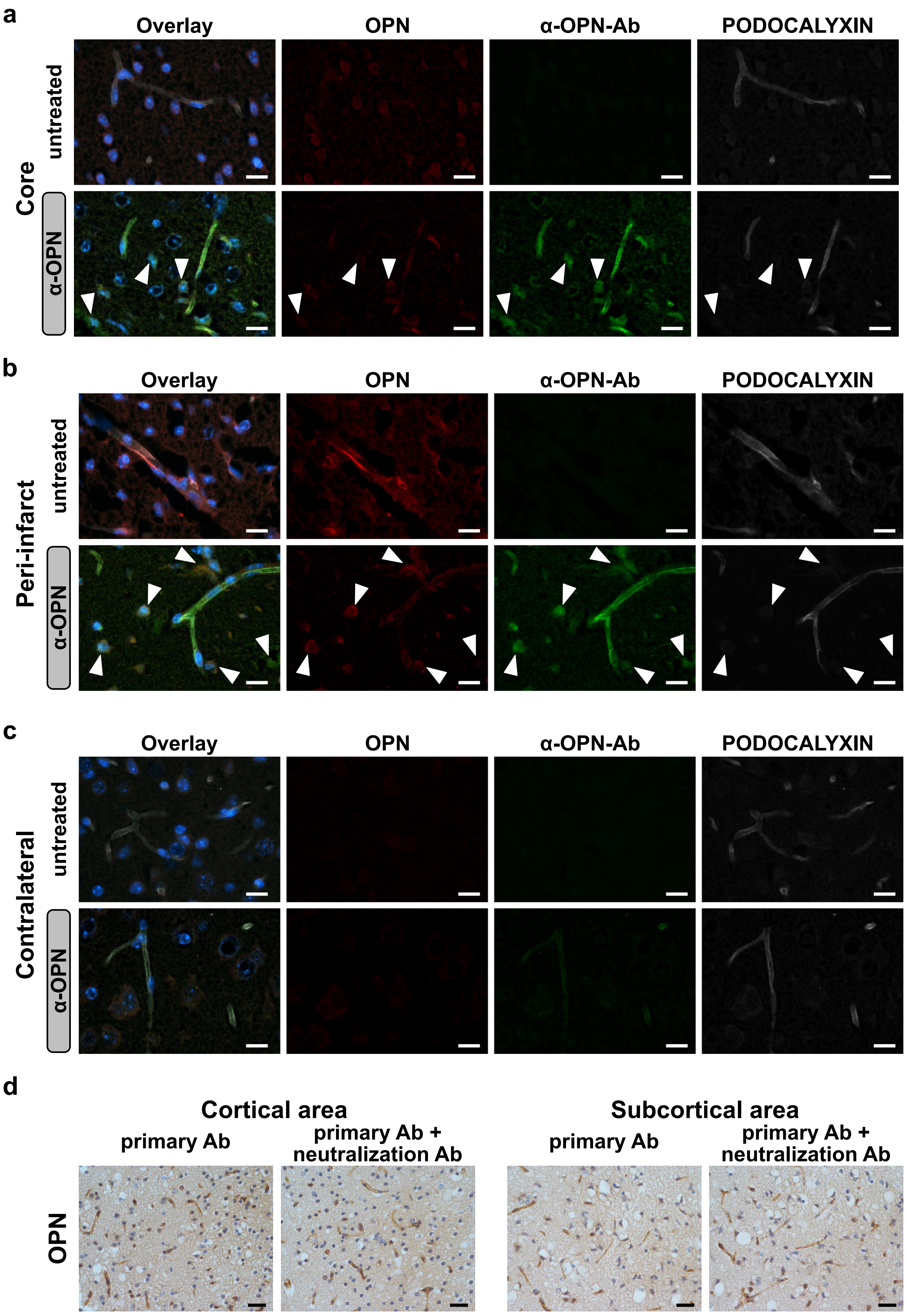

Supplementary Figure 17. CNS distribution of the therapeutic anti-OPN antibody in mice. Representative images of immunofluorescence staining for osteopontin (OPN, red), rabbit polyclonal antibody), anti-OPN antibody (α-OPN-Ab, green, therapeutic goat polyclonal antibody) and podocalyxin (white) in the infarct core (**a**), peri-infarct region (**b**) and contralateral hemisphere (**c**) of untreated mice and mice treated with the α-OPN antibody after ischemic stroke. White arrowheads indicate therapeutic α-OPN antibody detected in the brain parenchyma. (**d**) Representative images from serial sections of OPN peroxidase staining on untreated ischemic mice with primary antibody (rabbit) co-incubated with or without anti-OPN goat therapeutic antibody. The unchanged intensity between the two conditions suggests no apparent effect of the therapeutic neutralization antibody (goat) on the detection of osteopontin by the rabbit polyclonal antibody. Scale bars: 10μm (**a-c**) and 20μm (**d**).

Suppl Figure 18

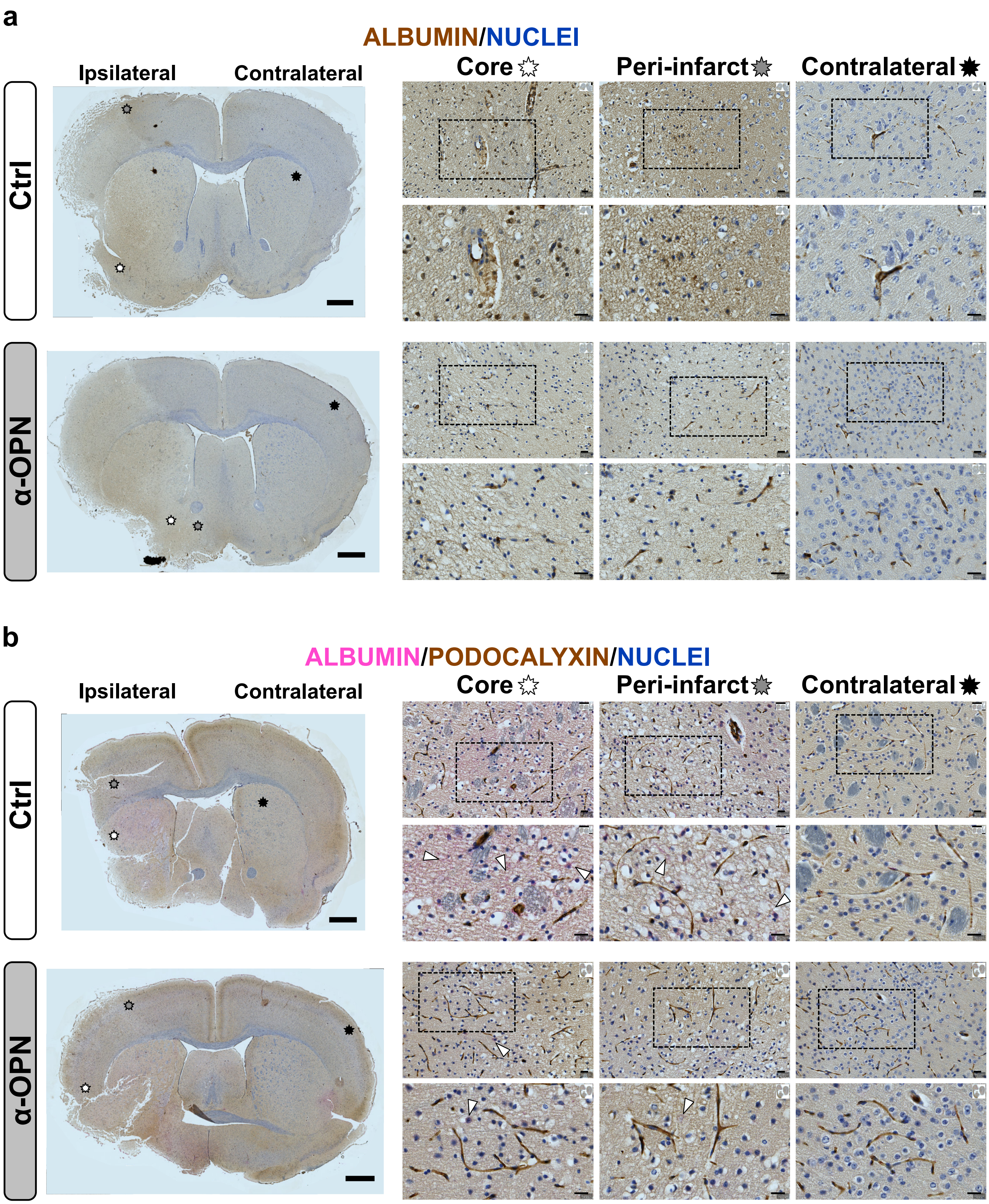

Supplementary Figure 18. Albumin staining for permeability assessment post anti-OPN treatment in ischemic mice. (**a**) Representative images of peroxidase staining for mouse albumin (brown) in Ctrl IgG and α-OPN antibody-treated mice 24 hours post occlusion. (**b**) Representative images of mouse albumin (pink) and vessels (Podocalyxin, brown) in Ctrl IgG and α-OPN antibody-treated mice. Nuclei are stained in blue. Images on part **b** are from a different set of mice compared to part **a** and Fig 8b. Scale 800μm for full brain section and 20μm for insets.

Suppl Figure 19

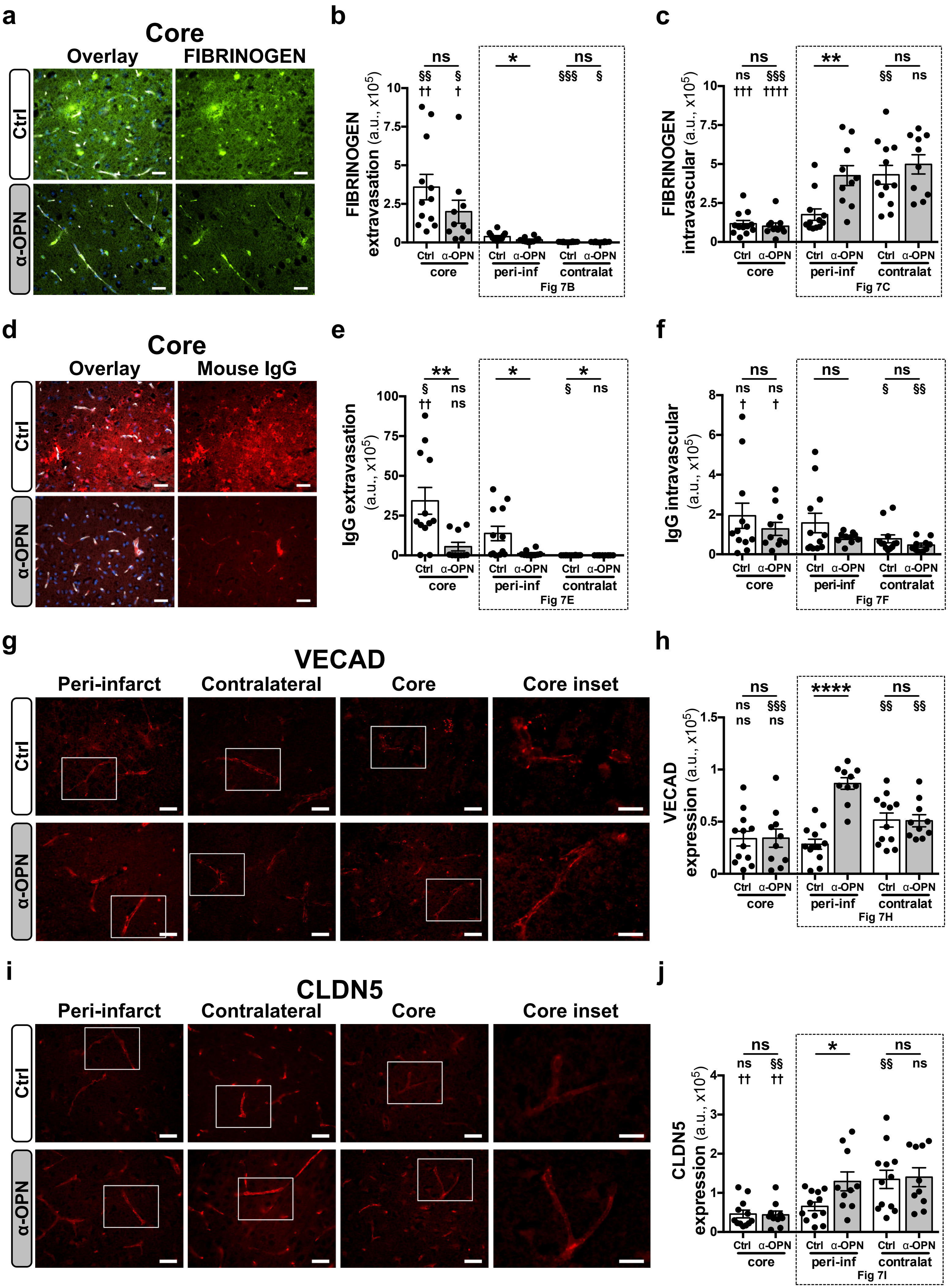

Supplementary Figure 19. Effect of the anti-OPN antibody therapy on BBB function in the infarct core in ischemic mice. (**a-c**) Representative images of fibrinogen immunofluorescence staining (green, **a**) and quantification of extravasated (**b**) and intravascular (**c**) fibrinogen in the core, peri-infarct region and contralateral hemisphere of Ctrl IgG and α-OPN antibody-treated mice. (**d-f**) Representative images of mouse immunoglobulin (IgG) immunofluorescence staining (red, **d**) and quantification of extravasated (**e**) and intravascular (**f**) IgG in the core, peri-infarct and contralateral hemisphere of Ctrl IgG and α-OPN antibody-treated mice. Podocalyxin (white) was used as vessel marker as shown in overlay pictures (**a, d**). (**g-j**) Representative images of EC adherens and tight junctions VECAD (**g**) and CLDN5 (**i**) stainings in the core of Ctrl IgG and α-OPN antibody-treated mice and corresponding quantifications (**h, j**). Quantifications were done utilizing three images/region/animal, $n = 12$ and $n = 10$ for Ctrl and α-OPN antibody, respectively. *§/† $P < 0.05$, **/§§/†† $P < 0.01$, §§§/††† $P < 0.001$, ****/†††† $P < 0.0001$ and not significant (ns) $P > 0.05$. * indicates two-tailed, unpaired t-test, with Welch's correction when variances were significantly different based on F-test, comparing the two treatment groups for the same region, § indicates two-tailed, paired t-test comparison of peri-infarct to core or contralateral regions within the same treatment group/animal, and † indicates two-tailed paired t-test comparison of the infarct core and contralateral regions within the same treatment group/animal. Scale bars: 20µm for (**a, d**); 10µm (**g**).

Suppl Figure 20

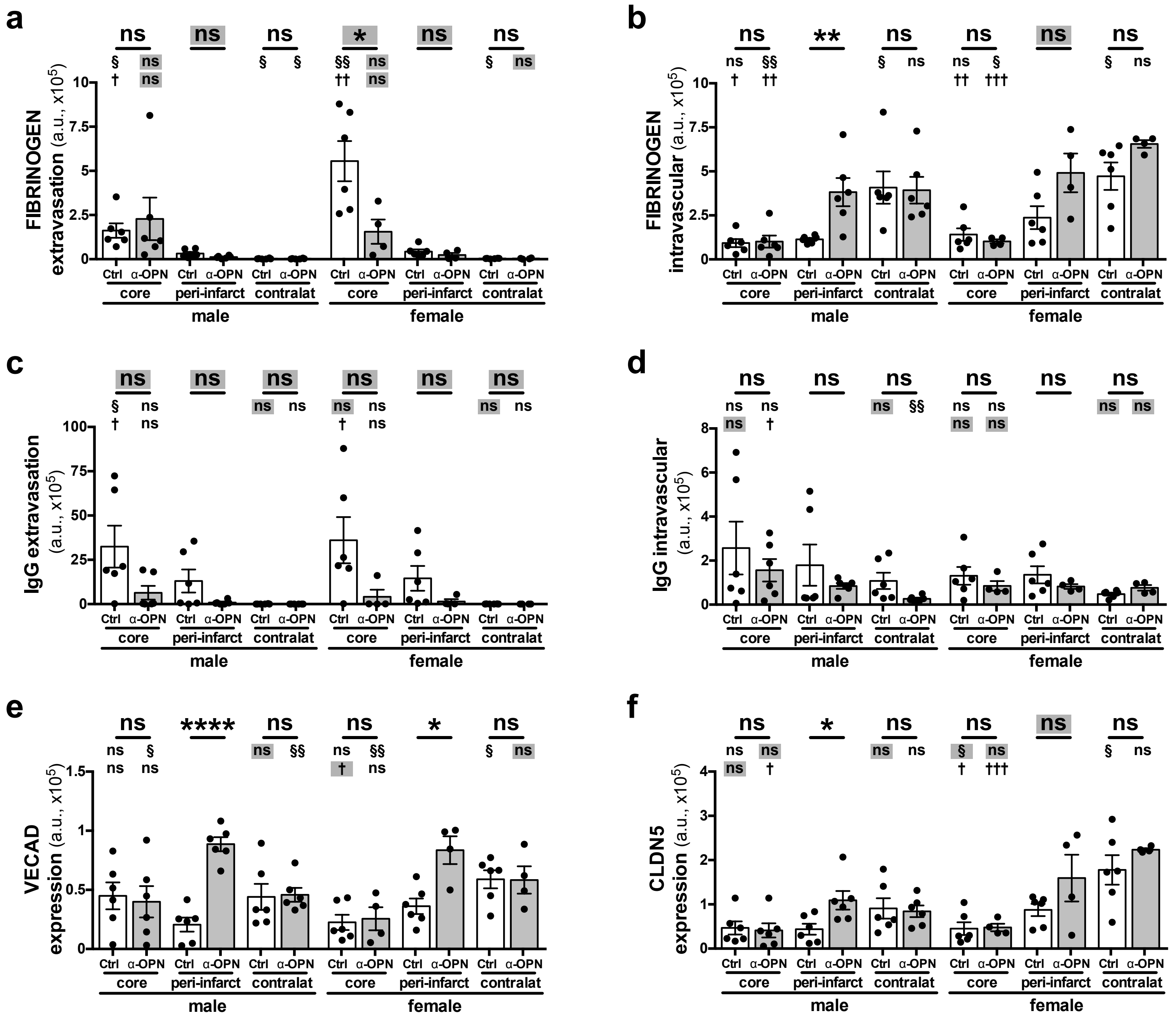

Supplementary Figure 20: Gender-related effects of anti-OPN antibody treatment on BBB function in ischemic mice. (**a-b**) Quantification of extravasated (**a**) and intravascular (**b**) fibrinogen in the core, peri-infarct region, and contralateral hemisphere of males and females Ctrl IgG and α-OPN antibody-treated mice. (**c-d**) Quantification of extravasated (**c**) and intravascular (**d**) mouse IgG in the core, peri-infarct and contralateral hemisphere of males and females Ctrl IgG and α-OPN antibody-treated mice. (**e-f**) Quantification of endothelial adherens and tight junctions VECAD (**e**) and CLDN5 (**f**) staining in the core of males and females Ctrl IgG and α-OPN antibody-treated mice. Three images per region and per animal were used, n = 6 for both Ctrl IgG treated males and females, and n = 6 and 4 for α-OPN antibody-treated males and females, respectively. */§/† P < 0.05, **/§§/†† P < 0.01, ††† P < 0.001, ****/ P < 0.0001 and not significant (ns) P > 0.05. * indicates two-tailed, unpaired t-test, with Welch's correction when variances were significantly different based on F-test, comparing the two treatment groups for the same region. § indicates two-tailed, paired t-test comparison of peri-infarct to contralateral or core regions within the same treatment group/animal, and † indicates two-tailed, paired t-test comparison of core to contralateral regions within the same treatment group/animal. Grey rectangles highlight the difference of significance observed in gender separated results from pooled results (Fig Suppl 19)

Suppl Figure 21

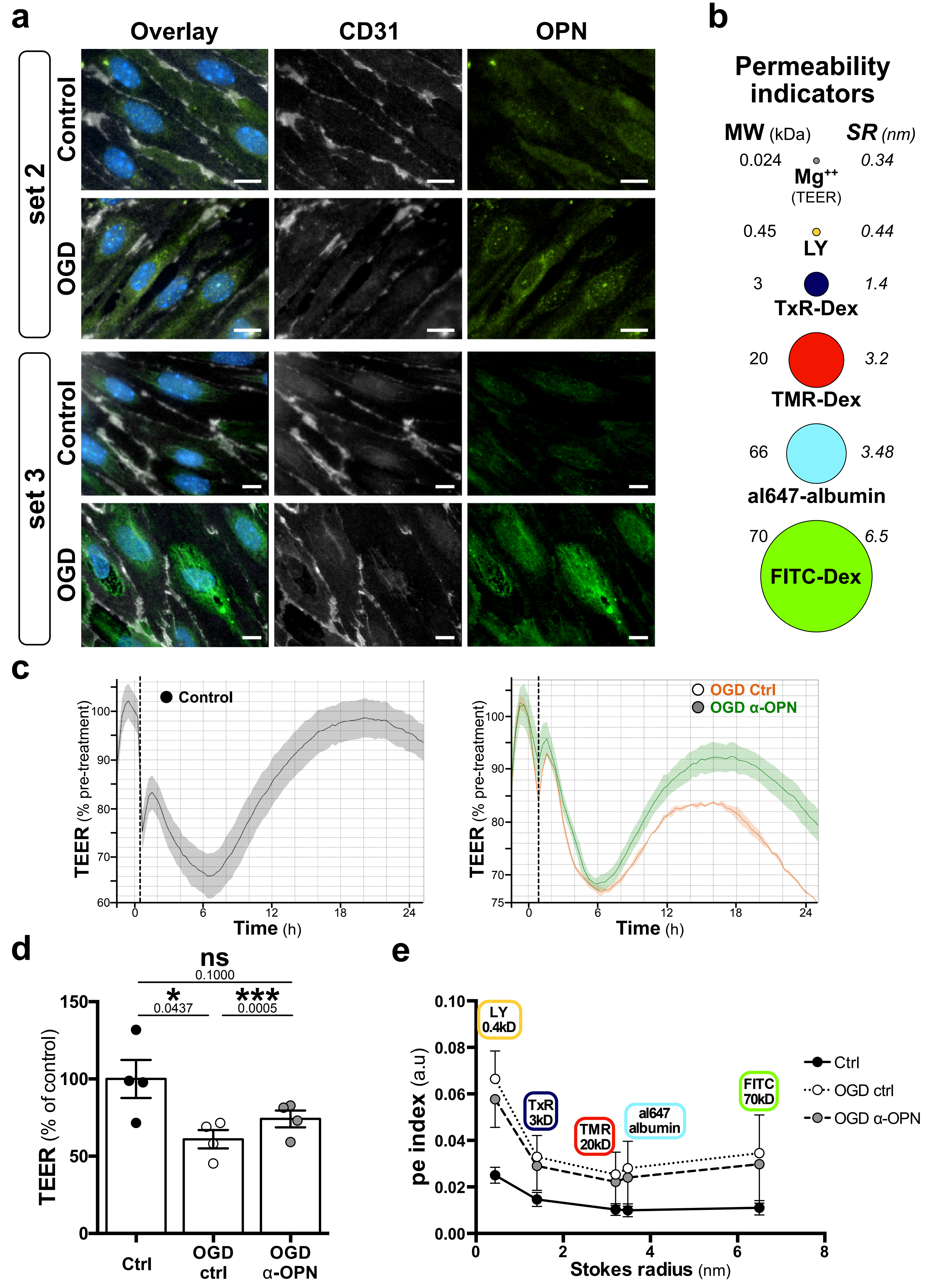

Supplementary Figure 21. OPN expression and permeability assessment post OGD *in vitro*. (**a**) Representative images of osteopontin (OPN, green) in endothelial monolayer 24 hours post oxygen-glucose deprivation (OGD) treatment. CD31 (white) was used as endothelial marker and DAPI (blue) to reveal nuclei. n = 2 independent experiments from Fig 8g. Scale bars: 10µm (**b**) Schematic representing the permeability indicators used *in vitro* along with their molecular weight (MW) and their Stokes radius (SR). The size of the circle is based on the Stokes radius; Dex = dextran. (**c**) Representative graph for continuous TEER values of the MBMEC monolayer in control normoxic conditions (treated with isotype control) and for OGD conditions – isotype control or anti-OPN antibody treated, normalized to pre-treatment TEER values. (**d**) 24 hours TEER values of MBMEC treated with isotype control or anti-OPN antibody during OGD normalized to the control; n = 4 independent experiment. (**e**) Raw permeability (pe) index values of several molecular weight fluorescent tracers through MBMEC monolayer subjected to OGD and treated with isotype control or anti-OPN antibody. n = 4 independent experiments. * $P < 0.05$; *** $P < 0.001$ and ns $P > 0.05$ by two-tailed, paired t-test.
